# Comparison of coded‐wire tagging with parentage‐based tagging and genetic stock identification in a large‐scale coho salmon fisheries application in British Columbia, Canada

**DOI:** 10.1111/eva.12711

**Published:** 2018-10-11

**Authors:** Terry D. Beacham, Colin Wallace, Kim Jonsen, Brenda McIntosh, John R. Candy, David Willis, Cheryl Lynch, Jean‐Sébastien Moore, Louis Bernatchez, Ruth E. Withler

**Affiliations:** ^1^ Fisheries and Oceans Canada Pacific Biological Station Nanaimo BC Canada; ^2^ Fisheries and Oceans Canada Regional Headquarters Vancouver BC Canada; ^3^ Département de Biologie, Institut de Biologie Intégrative et des Systèmes (IBIS) Université Laval Québec QC Canada

**Keywords:** coded‐wire tags, Coho salmon, fishery management, genetic stock identification, genotyping by sequencing, parentage‐based tagging

## Abstract

Wild Pacific salmon, including Coho salmon *Onchorynchus kisutch*, have been supplemented with hatchery propagation for over 50 years in support of increased ocean harvest and conservation of threatened populations. In Canada, the Wild Salmon Policy for Pacific salmon was established with the goal of maintaining and restoring healthy and diverse Pacific salmon populations, making conservation of wild salmon and their habitats the highest priority for resource management decision‐making. A new approach to the assessment and management of wild coho salmon, and the associated hatchery production and fishery management is needed. Implementation of parentage‐based tagging (PBT) may overcome problems associated with coded‐wire tag‐based (CWT) assessment and management of coho salmon fisheries, providing at a minimum information equivalent to that derived from the CWT program. PBT and genetic stock identification (GSI) were used to identify coho salmon sampled in fisheries (8,006 individuals) and escapements (1,692 individuals) in British Columbia to specific conservation units (CU), populations, and broodyears. Individuals were genotyped at 304 single nucleotide polymorphisms (SNPs) via direct sequencing of amplicons. Very high accuracy of assignment to population (100%) via PBT for 543 jack (age 2) assigned to correct age and collection location and 265 coded‐wire tag (CWT, age 3) coho salmon assigned to correct age and release location was observed, with a 40,774—individual, 267—population baseline available for assignment. Coho salmon from un‐CWTed enhanced populations contributed 65% of the catch in southern recreational fisheries in 2017. Application of a PBT‐GSI system of identification to individuals in 2017 fisheries and escapements provided high‐resolution estimates of stock composition, catch, and exploitation rate by CU or population, providing an alternate and more effective method in the assessment and management of Canadian‐origin coho salmon relative to CWTs, and an opportunity for a genetic‐based system to replace the current CWT system for coho salmon assessment.

## 1 INTRODUCTION

Wild Pacific salmon, including Coho salmon *Onchorynchus kisutch*, have been supplemented with hatchery propagation for over half a century in support of increased ocean harvest and conservation of threatened populations. The potential negative effects of hatchery production and the associated exploitation pressure that may be applied to natural populations have long been recognized and have gained prominence in recent years (Araki, Berejikian, Ford, & Blouin, 2008; Hilborn, 1992; Jones, Cornwell, Bottom, Stein, & Anlauf‐Dunn, 2018; McClure et al., 2008), leading to calls for increased responsibility in management of hatchery production and of the mixed‐stock fisheries it supports (Flagg, 2015; HSRG, 2014). In Canada, the Wild Salmon Policy (WSP) for Pacific salmon was established with the goal of maintaining and restoring healthy and diverse Pacific salmon populations, making conservation of wild salmon and their habitats the highest priority for resource management decision‐making (Fisheries and Oceans Canada 2005). Fisheries and hatchery supplementation (termed enhancement in Canada) are to be managed in such a way as to ensure that wild populations are safeguarded and harvest benefits are sustainable. Wild salmon populations are identified and maintained in conservation units (CUs) that reflect their geographic, ecological, and genetic diversity.

Coho salmon are caught in commercial, recreational, and First Nations fisheries in British Columbia, and determination of the impact of these fisheries is of fundamental importance to status assessment for wild populations of conservation concern and management of large‐scale hatchery production. Current and historical assessment of fisheries impacts has been conducted with the application of coded‐wire tags (CWTs; Jefferts, Bergmann, & Fiscus, 1963). CWTs are applied to juvenile fish prior to their hatchery release and recovered from adult fish heads collected from fisheries, hatchery broodstocks, and in‐river escapement sampling. Once recovered, the tags are decoded to determine the hatchery origin and age of the individual fish. Originally, only coho salmon marked with a CWT also received an adipose fin clip prior to hatchery release, with the externally visible clip mark allowing CWT‐marked fish to be identified visually and sampled from fisheries or river collections.

Since the late 1990s, all coho salmon released from many hatcheries in southern British Columbia (BC), Washington, and Oregon have received an adipose fin clip (termed mass marking) in order to facilitate mark‐selective fisheries intended to harvest hatchery salmon only, with most clipped individuals carrying no CWT. This approach has resulted in reduced exploitation of naturally spawned coho salmon, especially in sport fisheries, but the presence of many adipose‐clipped salmon without a CWT has impaired the efficiency of CWT recovery. In spite of implementation of an electronic tag detection system to pre‐screen a portion of the commercial catch to identify salmon with a CWT, the processing of many heads without a CWT from voluntary recreational recoveries and the increasing costs of CWT application and recovery have caused degradation of the information obtained from the current Canadian coho salmon assessment program.

For coho salmon, fisheries impacts are evaluated through the Fishery Regulation Assessment Model (FRAM; PFMC, 2008). The FRAM is a discrete‐time‐step, age‐structured computer model used to predict the impacts of a variety of proposed fishery regulations for a single management year in implementation of provisions of the Pacific Salmon Treaty (PST) related to coho salmon. Canadian and American assessment staff use the bilaterally agreed upon FRAM to estimate fishery impacts. Prior to each fishing season, both countries incorporate stock‐specific conservation constraints identified in the coho salmon chapter of the PST into FRAM evaluation of planned fisheries. Application of the FRAM requires use of data derived from fishery and escapement recoveries of CWTs and terminal area run size estimates for return years from a base period (1986–1992). The current expected catch of a specific stock is estimated as the product of (the expected abundance of the stock in a fishery) * (the base period exploitation rate) * (a correction factor) that relates the expected catch or effort in a particular year relative to that observed in the base period. Stock distribution and migration is assumed constant over time and is represented by the average distribution of CWT recoveries during the base period. However, differences between the abundance, distribution, and migration pattern of stocks during the base period and the year being evaluated will decrease the accuracy of the estimated stock‐specific exploitation rates for the fishery under evaluation. Significant deficiencies in FRAM model predictions for Chinook salmon fishery assessment due to the use of incomplete and outdated baseline data have recently been demonstrated (Moran, Dazey, LaVoy, & Young, 2018).

For coho salmon, utility of the CWT system has been eroded by the extensive release of adipose fin‐clipped individuals without CWTs and by the sharply declining number of CWTs recovered from recent fishery sampling. Through direct query of the Regional Mark Information System (RMPC, 2018), it was determined that there were 2,664 CWTs (average of 888 CWTs annually) recovered from commercial and recreational fisheries in BC between 2014 and 2016, of which 1,172 (391 annually) CWTs were from coho salmon of Canadian origin. In comparison, the average annual number of CWTs recovered from coho salmon during the seven‐year FRAM base period was 27,119 CWTs, of which 15,436 CWTs were from coho salmon of Canadian origin. Thus, the average annual number of CWTs recovered from BC fisheries during 2014–2016 was 3.3%, and those of Canadian origin was 2.5%, of the respective annual numbers for the base period, largely due to a substantial reduction in catch but also coincident with reduction in the number of CWTs applied. Since estimation of fishery impacts with the FRAM is dependent on CWT recovery information, the greatly decreased number of CWT recoveries in recent years will have increased the variance of the estimated stock‐specific catch and exploitation rates in fisheries (Hinrichsen et al., 2016; Reisenbichler & Hartmann, 1980).

A new, cost‐effective approach to the assessment and management of wild coho salmon, and the associated hatchery production and fishery management is needed. Anderson and Garza (2006) noted that parentage‐based tagging (PBT) provides equivalent information (hatchery of release, age of individual) for hatchery fish as do CWTs; implementation of PBT thus may overcome problems associated with CWT‐based assessment and management of coho salmon fisheries in BC. Additionally, PBT provides a means of improved hatchery broodstock management, as well as assessment of hatchery‐wild interactions in salmonids. Unlike CWT‐based management, PBT‐informed hatchery and fishery management would benefit from the complete adipose‐clipping of hatchery‐produced salmon. A significant advantage of the combination of mass marking and PBT implementation is the capability to identify visually, sample, and if desired, remove hatchery fish of local and stray origin in threatened wild populations. Moreover, PBT entails genotyping the entire hatchery broodstock and enables the identification of all hatchery progeny by parentage assignment (Anderson, 2012; Wang, 2016), thus enabling a “mark rate” of virtually 100% of hatchery fish. Steele et al. (2013) demonstrated the equivalency of CWT and PBT in an initial evaluation of population and age assignment in steelhead trout (*Oncorhynchus mykiss*) of the Snake River basin in the Columbia River drainage. Hess et al. (2016) expanded the approach by using both PBT and genetic stock identification (GSI) to investigate run timing of steelhead trout in the upper Columbia River drainage. These applications confirmed the capability of a combined PBT‐GSI technology to provide equivalent or better identification of fish as the CWT method, but were limited in geographic scale. In this study, we examine whether the existing PBT and GSI system of identification for Canadian‐origin coho salmon (Beacham et al., 2017) can provide the information required for improved assessment and management of coho salmon mixed‐stock fisheries in British Columbia, covering a much broader geographic range of coho populations from Alaska to Oregon.

Although proper management of hatchery production and associated fishery exploitation requires properly defined objectives and monitoring (Flagg, 2015), it is not currently possible to evaluate each Canadian enhancement project separately (Tompkins, Hamilton, Bateman, & Irvine, 2016). For coho salmon, CWTs are not applied to releases from some of the largest hatcheries in southern BC (Chilliwack River, Capilano River, Chehalis River, Conuma River, Nitinat River, and Tenderfoot Creek) due to funding limitations, and thus, their specific contributions to highly mixed‐stock ocean fisheries are unknown. In fact, CWTs are applied only to coho salmon juveniles released from six Fisheries and Oceans Canada large production hatcheries: three on the east coast of Vancouver Island (Quinsam River, Puntledge River, and Big Qualicum River), one on the west coast of Vancouver Island (Robertson Creek), one in the lower Fraser River drainage (Inch Creek), and one in the Thompson River (a major tributary of the Fraser River) drainage (Spius Creek, where the Salmon River and Eagle River juveniles may be reared). CWTs are also applied at smaller facilities (Seymour River near Vancouver, Toboggan Creek and Zymacord River in the Skeena River drainage in northern BC) and some naturally spawned index populations in northern BC.

The 43 coho salmon CUs originally identified by Holtby and Ciruna (2007) have been modified to a current number of 44 CUs. Price, Rosenberger, Taylor, and Stanford (2014), Price, English, Rosenberger, MacDuffee, and Reynolds (2017) suggested that any suitable assessment technique must provide individual resolution for all CUs to meet the conservation requirements of Canada's WSP. There can be possible inconsistencies between CUs and existing fishery management units (MUs) (Irvine & Fraser, 2008), with differences between CUs and MUs challenging managers who are responsible for both assessing biological status of CUs and fishery objectives for MUs (Holt & Irvine, 2013). Given the limited distribution of coho salmon populations marked with CWTs in BC, it is clear that CWTs cannot provide the CU resolution recommended by Price et al. (2014), Price et al. (2017) for wild population assessment, nor the MU resolution noted by Holt and Irvine (2013) for hatchery and fishery assessment and management.

The current study is an evaluation of the application of the PBT‐GSI methodology outlined by Beacham et al. (2017) to coho salmon fisheries in BC to determine whether the genetic technologies can be used to provide more information on fishery contributions by hatchery and CU than is available from CWTs. Commercial and recreational coho salmon fisheries, and river escapements for selected populations, were sampled for both CWTs and genotypes. We evaluated the population‐level resolution obtained from CWTs and the genetic methodology by CU for all 2017 and some 2016 fisheries in which coho salmon were caught, catch estimation by CU for the fisheries sampled, and stock‐specific exploitation rate for selected populations of coho salmon in BC. Genotyping by sequencing methodology was used to genotype coho salmon at 304 single nucleotide polymorphisms (SNPs) in 304 amplicons. Complete broodstock genotyping for PBT analysis was conducted in 2014 for 20 hatchery‐enhanced populations that included genotyping 6,061 individuals (96.4% genotyping success rate), and a stock identification baseline comprising some 267 populations ranging from southeast Alaska to Oregon was employed for GSI. A comparison of the population‐specific contributions to mixed‐stock fisheries, catch, and exploitation rates estimated with CWTs and PBT‐GSI technologies was made. We conclude that a genetic approach can emulate and improve upon the results available from the current CWT program for assessment and management of coho salmon enhancement and fisheries in BC, and provide critical information to improve wild coho salmon assessment and conservation.

## 2 METHODS

### 2.1 Fishery sample collection

**Figure 1:**
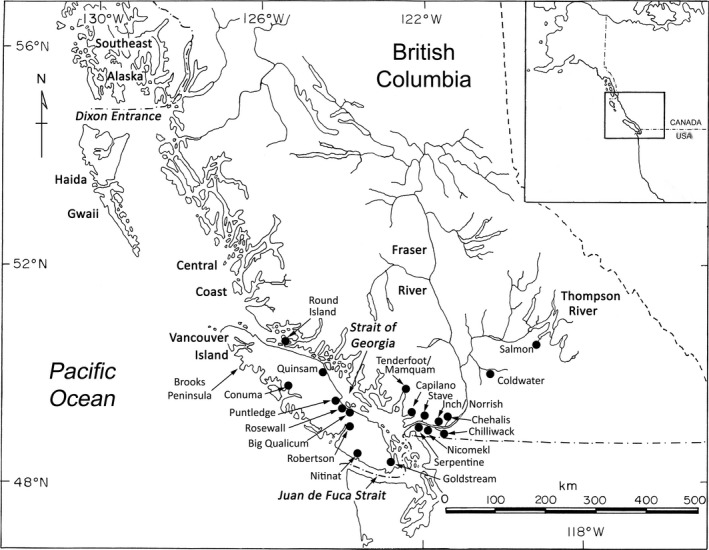
Map indicating geographic locations for fishery sampling and 20 populations for which parentage‐based tagging was applied in estimation of stock composition

A total of 8,006 individuals were genotyped from fishery samples collected in 2016 and 2017. In 2016, samples were collected from adipose fin‐clipped and unclipped coho salmon landed in the recreational fishery in the Strait of Georgia, Juan de Fuca Strait, and along the west coast of Vancouver Island. The only exceptions were Area 21 and Area 121, where only clipped individuals were sampled, and Areas 20‐1 to 20‐4 in August, where only clipped individuals were sampled. Samples were pooled for analysis as outlined in Table 1, with the locations of statistical areas outlined by https://www.pac.dfo-mpo.gc.ca/fm-gp/maps-cartes/areas-secteurs/index-eng.html. Additional coho salmon samples (both clipped and unclipped) were obtained from a troll test fishery operating near Brooks Peninsula off the northwest coast of Vancouver Island, a gillnet test fishery targeting sockeye salmon (*Oncorhynchus nerka*) near Round Island in northern Johnstone Strait, and a commercial demonstration freezer troll fishery in the central coast of BC (Figure 1).

**Table 1:** Reporting region and DFO statistical areas within regions for amalgamation of catch samples. Locations of statistical areas are outlined on https://www.pac.dfo-mpo.gc.ca/fm-gp/maps-cartes/areas-secteurs/index-eng.html

Fishery	Reporting region	Statistical area
Recreational	Johnstone Strait (JST)	A11 to A13
	Strait of Georgia‐north (SOG‐n)	A14 to A16
	Strait of Georgia‐south (SOG‐s)	A17 to A18, A19‐7 to A19‐12, A28, A29
	Strait of Georgia/Juan de Fuca Strait (SOG/JDF)	A19‐1 to A19‐6, A20‐5
	West coast Vancouver Island/Juan de Fuca Strait (WCVI/JDF)	A20 (except A20‐5)
	Southwest Vancouver Island (SWVI 21/121)	A21, A121
	Southwest Vancouver Island‐inshore (SWVI‐inshore)	A23, A24
	Southwest Vancouver Island‐offshore (SWVI‐offshore)	A123, A124
	Northwest Vancouver Island‐inshore (NWVI‐inshore)	A25 to A27
	Northwest Vancouver Island‐offshore (NWVI‐offshore)	A125 to A127
Test	Brooks Peninsula	A27, A127, A126
	Round Island	A12
Troll	Central Coast	A6, A7, A8

In 2017, for the northern (Area F) freezer troll fishery, selected freezer boats (28% of fleet) were required to keep heads of all coho salmon caught, with the mark type (adipose fin clipped or not) unknown for an individual head. Upon landing, the heads were counted and checked electronically for CWTs and randomly sampled to a maximum of 50 heads per delivery. If a CWT was detected, the head was sent to a central CWT head recovery laboratory in Vancouver, BC, where the DNA sample was subsequently taken. If a CWT was successfully recovered and decoded from an individual head from commercial, recreational, or First Nations fisheries, and a genotype was successfully obtained for the individual sample, then the genotypes of all of these individuals were pooled into a single mixed‐stock sample of known origin and known age in order to evaluate accuracy of stock compositions by CU and population. Field DNA samples were taken only from individuals with no CWTs detected. In the northern ice boat troll fishery, the clip status of the fish in the catch was known. Samples of clipped coho salmon were obtained from this fishery as an ancillary aspect of standard Fisheries and Oceans Canada contract catch sampling for CWTs. Only clipped individuals were examined through this program, with similar sampling protocols as outlined in the freezer boat sampling. In particular, only clipped fish not containing a CWT were sampled in the field through this program, with heads containing a CWT sent to the head recovery laboratory in Vancouver. Clipped fish constituted 1.6% of the catch in the fishery, largely due to the wild origin of the catch, so it was important to obtain samples from unclipped individuals as well. Samples of unclipped individuals were obtained by sampling landings for a maximum of 25 individuals per vessel, with a maximum of 100 individuals sampled per day. Samples from the Central Coast freezer troll demonstration fishery were obtained via a similar protocol as outlined for the northern freezer troll sampling. The origin of the samples from the recreational fishery in BC included voluntary head recoveries of adipose fin‐clipped coho salmon from recreational fisheries in BC, as well as some creel sampling of recreational catches. Samples from the recreational fishery were derived from clipped individuals, but they may not have been marked with a CWT when delivered to the CWT head recovery laboratory. Thus, samples in 2017 were obtained from all individuals that would be routinely processed under the CWT recovery program for coho salmon in BC. In addition, samples were collected from a recreational fishery (clipped and unclipped individuals) from Haida Gwaii near Langara Island, a recreational fishery near Rivers Inlet (Area 8, clipped and unclipped individuals) in the central coast, the previously noted ice boat northern troll fishery, and direct creel sampling (clipped and unclipped individuals) of recreational fisheries in southern BC.

Monthly catch composition estimates were determined for clipped individuals from various fisheries in BC. Seasonal catch composition estimates were determined by recording the number of individual genotypes available from the fishery sampling in each month and weighting individual monthly samples by the recorded catch in the month. Individuals were randomly chosen for months in which the number of available genotypes was in excess of the number required for the seasonal sample. For example, if 60% of the annual catch was recorded in one month and 200 genotypes were available from the fishery, then all individuals sampled would be included in the seasonal sample, but contributions of individuals from other months were scaled relative to the monthly contribution to the seasonal catch. If the catch in one other month was 20% of the annual catch, and 150 samples were available, then 200 * (20%/60%) = 67 genotypes were randomly chosen from the available 150 genotypes for inclusion into the seasonal sample.

In the northern BC troll fishery, samplers electronically checked clipped individuals for the presence of a CWT, and heads from those individuals containing a CWT were subsequently sent to the central laboratory for CWT recovery and tissue sampling for subsequent genotyping. Tissue samples from clipped individuals with no CWTs were directly provided for genotyping. In order to estimate monthly and seasonal stock composition of clipped individuals in this fishery, we pooled the genotypes obtained from direct fishery sampling of clipped individuals with those subsequently sent to the central laboratory for CWT recovery in order to provide an appropriate pool of clipped individuals to estimate monthly and seasonal stock compositions of clipped individuals in the fishery. In each sample analyzed over all BC fisheries, the number of individuals identified via PBT relative to the number of genotypes in the sample was tabulated, and summarized over sample, fishery, and season.

### 2.2 Escapement sample collection

In 2016, adipose fin‐clipped jacks (age 2 years) were sampled from eight populations, with 573 individuals collected and genotyped successfully. Jacks were defined on the basis of body size during sample collection, with jacks visibly smaller than age 3‐year spawners. Populations sampled were as follows: Robertson Creek, Quinsam River, Puntledge River, Big Qualicum River, Goldstream River, Chilliwack River, Stave River, and Inch Creek. The objective of the sampling was to obtain a sample of known origin and known age based upon known collection location in order to evaluate accuracy of assignments of the jacks to both CU and population, under the assumption of no straying among populations for the individuals sampled.

In 2017, escapements (non‐broodstock hatchery and river returns, clipped individuals only) were sampled where sampling was feasible. Escapement sampling from 13 populations was conducted, and 1,692 individuals were genotyped. Escapement samples were not available from all 20 populations for which it was possible to assign individuals in the non‐broodstock escapements via PBT. The objective of the sampling was to evaluate straying rates among populations, and for those hatcheries (Chilliwack River) where parental genotypes were assigned to specific release groups, provide information on the relative rates of return of different release groups. As relative rates of return for the release groups were considered ancillary to the main focus of the study, no results from these returns were included in the current study.

### 2.3 Exploitation rate

We estimated exploitation rate of adult coho salmon in BC fisheries via both CWTs and genetics. Exploitation rate for a population was defined as adult catch/(adult catch‐escapement). For CWTs, the observed number of CWTs was corrected by “no‐pin” tag loss rates and was expanded by the population's tag‐specific marking rate summed over tag codes and expanded again by the sampling rate for the fishery in order to estimate catch of hatchery‐origin individuals. The observed number of CWTs in escapement sampling was expanded in a similar manner in order to estimate the hatchery contribution to the escapement. For genetics, the seasonal kept catch of adipose fin‐clipped individuals in a fishery was multiplied by a seasonal stock composition estimate in order to estimate population‐specific catch. The abundance of hatchery‐origin escapement was calculated as the estimated escapement multiplied by the proportion of adipose fin‐clipped adults observed in the escapement. If part of the juvenile production was not adipose fin clipped upon release from the hatchery, then the hatchery contributions to both catch and escapement were underestimated by this method.

### 2.4 Genetic stock identification baseline

The initial baseline was outlined by Beacham et al. (2017) and consisted of 20,242 individuals from 117 populations, with the distribution of populations ranging from southeast Alaska to Puget Sound in Washington State. The baseline has been subsequently expanded to include 40,774 individuals from 267 populations, ranging from southeast Alaska to Oregon. The primary expansion of the baseline included a survey of additional populations in southern BC, coastal Washington, the Columbia River drainage, and Oregon. The full baseline is outlined in Supporting Information Table S1, with the populations from BC arranged by CU, and with United States of America (US) populations arranged by geographic (reporting) region. Beacham et al. (2017) had previously demonstrated a strong regional population structure and reported that, overall 117 populations, average self‐assignment accuracy to population (weighted by population sample size) was 85.5% for individuals with an assignment probability <0.50 excluded and 92.8% for individuals with an assignment probability <0.85 excluded. High levels of accuracy for GSI assignment of individuals to specific populations, when combined with assignments via PBT, provide a powerful technique for assignment of individuals of unknown origin to some populations.

### 2.5 Library preparation and genotyping

The detailed procedure for library preparation and genotyping was outlined by Beacham et al. (2017). Summarized briefly, DNA was extracted from coho salmon tissue samples via a Chelex extraction and the DNA concentration normalized to 40 ng/μl with a Tecan LiHa robot. A panel of primers designed to amplify specific segments of DNA that contained SNPs of interest was applied to the extracted DNA, with primer sequences for each amplicon outlined by Beacham et al. (2017; Supporting Information Table S2). The initial multiplex PCR amplification of 304 target amplicons was conducted with a cocktail of 2μl of normalized DNA extract, 5μl of 2X Ion Agriseq primer pool, 2μl of Ion Agriseq HiFi mix, and 1μl of ddH_2_O. Thermal cycling was conducted in 96‐well PCR plates (one individual per well) with the following conditions for PCR: 99°C – 2 min; 17 cycles (99°C – 15 s, 60°C – 4 min); 10°C hold.

Following the initial PCR, a second step employing a thermal cycler was conducted that partially digested the primers on the amplicons, and the reaction conducted with the following conditions: 50°C – 10 min; 55°C – 10 min, 60°C – 20 min, 10°C hold. A third and final step employing a thermal cycler was initiated to ligate the barcodes (768 individual codes) to the amplicons and was conducted with the following conditions: 22°C – 30 min; 70°C – 10 min, 10°C hold. Libraries were purified by addition of 22.5 μl of Agencourt® AMPure® XP magnetic beads to each library, the plate was placed on a magnetic rack, supernatant discarded, and the beads washed twice in 70% ethanol. The purified libraries were then eluted with 25 μl of low TE, and 20 μl of the supernatant transferred to a fresh 96‐well tray. Next, each of the 768 prepared libraries was pooled into a single tube for processing on the Ion Chef® (Thermo Fisher Scientific). Two tubes of pooled libraries were processed consecutively on the Ion Chef, and thus, 1,536 individuals were processed on a single run of the Ion Chef. One tube of the pooled libraries was loaded on to each P1® chip v3, and thus, amplicons from 768 individuals were distributed on each P1 chip, with 1,536 individuals processed between two chips. The chips were then loaded on to the Ion Torrent Proton sequencer. After the sequencing run was completed, comparisons with the reference genome of the rainbow trout (*O. mykiss*) (Berthelot et al., 2014), supplemented with the sequences containing the observed coho salmon SNPs, were conducted with Proton software Variant Caller®, and SNP genotypes at the sites specified by the hotspot file within target regions were called by Variant Caller. The hotspot file contained 304 SNP sites, with one SNP scored at each amplicon. Genotypes at all available SNPs for an individual were assembled to provide a multi‐locus individual genotype. The species identification SNP *OkiOts_120255‐113* (Starks, Clemento, & Garza, 2016) and sex identification SNP *Ots_SEXY3‐1* were omitted from subsequent parentage and GSI analyses, leaving 302 SNPs.

### 2.6 Identification of individuals

As noted previously, PBT and GSI were used concurrently to estimate fishery stock composition. Initially, PBT was used, and the analysis was conducted where the genotypes of individuals to be identified were matched to the genotypes of prospective parents (COLONY, Jones & Wang, 2010; Wang, 2016). If all individuals in a hatchery broodstock are sampled and subsequently genotyped, then all offspring from the broodstock are genetically marked. The genotypes of individuals of unknown origin are statistically compared with the genotypes of potential parents, and if a match is made, the offspring are assigned to the parents, and thus, the origin and age of the individuals are determined. Parentage assignment software was utilized to assign offspring to parents, as COLONY can produce assignments when the genotype of one of the parents is missing, either due to a missing parental sample, or failure to produce a parental genotype from an existing sample. Given that PBT assignments for 20 potential populations were evaluated for each fishery sample, COLONY was run with all broodstock sampled during 2014 input as a single unit for analysis of fishery samples, with no differentiation among populations. Although the COLONY assumption of a single population in the parent pool was violated, analysis of known‐origin samples indicated that very high levels of accuracy were achieved in assignments when pooling of potential parents in contributing populations was conducted. Two‐parent assignments were accepted only when both assigned parents originated from the same population, otherwise the individual was passed to genetic stock identification (GSI) for potential assignment. Two‐parent and single‐parent assignments were accepted only when the probability of correct assignment was ≥0.85 for the parent pair, otherwise the individual was passed to potential assignment by GSI. Additionally, for single‐parent assignments to be accepted, the PBT assignment to population had to be part of the CU assigned to the individual via GSI. Individuals for which no prospective parents were identified in the broodstock were passed to GSI for potential assignment. Polygamous mating was assumed for the COLONY analysis. Simple pairwise comparisons between offspring and potential parents were conducted. The baseline for individuals sampled in the 2016 escapements (jacks) and 2017 fisheries included all broodstocks sampled in 2014, as these individuals are predominately three years of age (Sandercock, 1991). Jacks in the 2017 escapement were identified via body size and subsequent assignment to parents in the 2015 hatchery broodstocks. Individuals with more than 120 missing genotypes were eliminated from further analyses. An estimated genotyping error rate of 1% was used for COLONY assignments. Previously, Beacham et al. (2017) had reported that an average genotyping error rate of 1.07% (1,220 discrepancies in 114,105 comparisons) or an allele error rate of 0.53% (1,220 discrepancies in 228,210 comparisons) was observed over the 302 SNPs scored. The parent pair output file was the basic file used in subsequent analyses.

The second method of individual identification is GSI, in which the genetic profiles of whole populations potentially contributing to a mixed‐stock sample are used to estimate the origin of each individual in the sample (RUBIAS; Moran & Anderson, 2018). This analysis was restricted to those individuals unassigned via COLONY. For each sample, individuals not assigned by COLONY were then assigned with RUBIAS. The population posterior means file was the basic file used for subsequent analyses, and this file contained the probability of assignment of the individual to each of the 267 populations in the baseline. Stock composition was estimated through the combination of files generated with both COLONY and RUBIAS. Individuals assigned via COLONY were assigned a probability of 1.00 of originating from the identified population, with a 0.00 probability assigned to all other populations in the baseline. This level of assignment accuracy via PBT was observed previously for coho salmon with the panel of SNPs employed in the current study (Beacham et al., 2017). These data were then combined with the probability of assignment for those individuals unassigned via COLONY to each of the populations in the baseline via RUBIAS. After an initial burn‐in of 25,000 iterations, the last 1,000 iterations from the Monte Carlo Markov chain from RUBIAS were used to estimate the origin of individuals and stock composition, with the mean allocation to each population in the baseline determined. Standard deviations of estimated stock compositions were also determined from the last 1,000 iterations from the Monte Carlo Markov chain. Stock composition by CU or reporting group was determined by summation of allocations to all populations in the baseline that belonged to the CU or reporting group under consideration.

## 3 RESULTS

### 3.1 Estimation of stock composition for known‐origin samples

Genotypes were available from 573 jacks sampled in 2016 across three CUs and eight populations in southern BC. Estimated stock composition by CU and reporting group for this combined sample was accurate, with an error utilizing only GSI of ≤0.4% by CU for three CUs present in the sample (Table 2). With both PBT and GSI utilized, the average error declined to ≤0.2% by CU for three CUs present in the sample (Table 2). The average error for 45 CUs or reporting groups absent in the sample was 0.0% by both GSI and PBT‐GSI. By population, 543 (94.8%) of the jacks were assigned via PBT with 100% accuracy with respect to population of origin and age (Table 3). Estimated stock composition for 20 populations where it was possible to use both PBT and GSI in estimation of stock composition was accurate. For the eight populations present in the sample of jacks, the average error utilizing GSI only was 0.5% per population, declining to 0.2% utilizing both PBT and GSI, under the assumption that the collection location was an accurate reflection of jack origin. There was an average 0.0% error for the 12 populations with no representation in the sample with utilization of either GSI or PBT‐GSI (Table 4). The largest error utilizing both PBT and GSI (underestimation by 0.6% of actual value) was observed in the Goldstream River population, which also displayed the lowest percentage (70.4%) of jacks identified via PBT (Table 3). PBT when combined with GSI produced more accurate estimates of CU‐specific and population‐specific stock composition than available with only GSI.

**Table 2:** Accuracy of regional (United States) and conservation unit (Canada) estimated stock composition (%) for a mixed‐stock sample of 573 known‐origin coho salmon jacks sampled in 2016 and 620 coded‐wire tagged individuals sampled in 2017 fisheries in BC. Standard deviation of the estimate is in parentheses

Region/conservation unit	2016 jacks			2017 CWT		
	True	GSI	PBT‐GSI	True	GSI	PBT‐GSI
Southeast Alaska	0.0	0.0 (0.0)	0.0 (0.0)	9.0	6.8 (1.1)	6.8 (1.1)
Alsek River	0.0	0.0 (0.0)	0.0 (0.0)	0.0	0.0 (0.0)	0.0 (0.0)
Lower Stikine	0.0	0.0 (0.0)	0.0 (0.0)	0.0	0.4 (0.3)	0.4 (0.3)
Lower Nass	0.0	0.0 (0.0)	0.0 (0.0)	6.3	6.4 (1.0)	6.2 (1.0)
Upper Nass	0.0	0.0 (0.0)	0.0 (0.0)	0.0	0.4 (0.3)	0.4 (0.4)
Portland Sound‐Observatory Inlet‐Portland Canal	0.0	0.0 (0.0)	0.0 (0.0)	0.0	0.0 (0.0)	0.0 (0.0)
Skeena Estuary	0.0	0.0 (0.0)	0.0 (0.0)	0.0	0.0 (0.0)	0.0 (0.0)
Lower Skeena	0.0	0.0 (0.0)	0.0 (0.0)	1.6	1.2 (0.5)	1.2 (0.5)
Middle Skeena	0.0	0.0 (0.0)	0.0 (0.0)	6.9	3.9 (0.8)	3.9 (0.9)
Upper Skeena	0.0	0.0 (0.0)	0.0 (0.0)	0.6	4.0 (1.0)	4.0 (0.9)
Haida Gwaii–Graham Island Lowlands	0.0	0.0 (0.0)	0.0 (0.0)	0.0	0.0 (0.0)	0.0 (0.0)
Haida Gwaii‐East	0.0	0.0 (0.0)	0.0 (0.0)	0.0	0.0 (0.0)	0.0 (0.0)
Haida Gwaii‐West	0.0	0.0 (0.0)	0.0 (0.0)	0.0	0.0 (0.0)	0.0 (0.0)
Northern Coastal Streams	0.0	0.0 (0.0)	0.0 (0.0)	0.0	0.0 (0.0)	0.0 (0.1)
Hecate Strait Mainland	0.0	0.0 (0.0)	0.0 (0.0)	0.0	0.7 (0.5)	0.6 (0.4)
Mussel–Kynoch	0.0	0.0 (0.0)	0.0 (0.0)	0.0	0.0 (0.0)	0.0 (0.0)
Douglas Channel–Kitimat Arm	0.0	0.0 (0.1)	0.0 (0.1)	0.0	0.0 (0.1)	0.0 (0.1)
Bella Coola–Dean Rivers	0.0	0.0 (0.0)	0.0 (0.0)	0.0	0.0 (0.0)	0.0 (0.0)
Rivers Inlet	0.0	0.0 (0.1)	0.0 (0.0)	0.0	0.0 (0.0)	0.0 (0.0)
Smith Inlet	0.0	0.0 (0.0)	0.0 (0.0)	0.0	0.0 (0.0)	0.0 (0.0)
Southern Coastal Streams–Queen Charlotte Strait–Johnstone Strait–Southern Fjords	0.0	0.0 (0.1)	0.0 (0.1)	0.0	0.0 (0.2)	0.0 (0.2)
Homathko–Klinaklini Rivers	0.0	0.0 (0.0)	0.0 (0.0)	0.0	0.0 (0.0)	0.0 (0.0)
Georgia Strait Mainland	0.0	0.0 (0.0)	0.0 (0.0)	0.0	0.0 (0.0)	0.0 (0.0)
Howe Sound–Burrard Inlet	0.0	0.0 (0.0)	0.0 (0.0)	0.6	0.0 (0.1)	0.0 (0.0)
East Vancouver Island–Georgia Strait	35.1	34.5 (2.0)	34.8 (2.0)	13.1	13.4 (1.4)	14.0 (1.4)
East Vancouver Island–Johnstone Strait–Southern Fjords	0.0	0.0 (0.0)	0.0 (0.0)	0.0	0.0 (0.1)	0.0 (0.0)
Nahwitti Lowland	0.0	0.0 (0.1)	0.0 (0.0)	1.8	1.7 (0.6)	1.7 (0.6)
West Vancouver Island	11.5	11.5 (1.4)	11.5 (1.3)	13.5	13.4 (1.4)	13.5 (1.4)
Clayoquot	0.0	0.0 (0.0)	0.0 (0.0)	0.0	0.2 (0.3)	0.2 (0.2)
Juan de Fuca–Pachena	0.0	0.0 (0.1)	0.0 (0.1)	0.0	0.0 (0.0)	0.0 (0.0)
Lower Fraser	53.4	53.9 (2.1)	53.6 (2.1)	18.5	18.7 (1.5)	18.8 (1.5)
Lillooet	0.0	0.0 (0.0)	0.0 (0.0)	0.0	0.3 (0.2)	0.2 (0.1)
Fraser Canyon	0.0	0.0 (0.0)	0.0 (0.0)	0.0	0.0 (0.0)	0.0 (0.0)
Interior Fraser	0.0	0.0 (0.0)	0.0 (0.0)	0.0	0.0 (0.0)	0.0 (0.0)
Lower Thompson	0.0	0.0 (0.0)	0.0 (0.0)	0.8	0.7 (0.3)	0.8 (0.4)
North Thompson	0.0	0.0 (0.0)	0.0 (0.1)	0.0	0.0 (0.0)	0.0 (0.0)
South Thompson	0.0	0.0 (0.0)	0.0 (0.0)	0.5	0.6 (0.3)	0.5 (0.3)
Boundary Bay	0.0	0.0 (0.0)	0.0 (0.0)	0.0	0.0 (0.0)	0.0 (0.0)
Nooksack River	0.0	0.1 (0.2)	0.1 (0.2)	2.6	0.9 (0.9)	1.0 (0.8)
Skagit River	0.0	0.0 (0.1)	0.0 (0.1)	0.5	2.3 (0.8)	2.2 (0.8)
Northern Puget Sound	0.0	0.0 (0.0)	0.0 (0.0)	3.9	5.8 (1.2)	5.5 (1.3)
Mid‐Puget Sound	0.0	0.0 (0.0)	0.0 (0.0)	5.2	6.2 (1.1)	6.3 (1.1)
Southern Puget Sound	0.0	0.0 (0.0)	0.0 (0.0)	1.0	0.9 (1.0)	0.6 (0.8)
Juan de Fuca Strait	0.0	0.0 (0.0)	0.0 (0.0)	0.0	0.2 (0.3)	0.1 (0.3)
Hood Canal	0.0	0.0 (0.0)	0.0 (0.0)	4.4	1.6 (0.5)	1.6 (0.6)
Coastal Washington	0.0	0.0 (0.0)	0.0 (0.0)	6.0	5.9 (1.0)	5.9 (1.0)
Columbia River	0.0	0.0 (0.0)	0.0 (0.0)	3.2	3.2 (0.7)	3.2 (0.7)
Oregon	0.0	0.0 (0.0)	0.0 (0.0)	0.0	0.0 (0.1)	0.0 (0.0)

**Table 3:** Percentage of adipose fin‐clipped jacks (age 2 years) sampled in 2016 and assigned via parentage analysis to parents in 2014 broodstock populations. Population of sampling was assumed to be the population of origin of the jacks for evaluation of accuracy of assignment

Population	Genotyped	Assigned	% assignment	% accuracy of population assignment
Robertson	66	66	100.0	100.0
Quinsam	69	68	98.6	100.0
Puntledge	21	19	90.5	100.0
Big Qualicum	84	84	100.0	100.0
Goldstream	27	19	70.4	100.0
Chilliwack	195	183	93.8	100.0
Inch	91	85	93.4	100.0
Stave	20	19	95.0	100.0
Total	573	543	94.8	100.0

**Table 4:** Estimated population‐specific stock composition of the 2016 jack sample (*N* = 573) and 2017 CWT sample (*N* = 620) for 20 populations with estimates derived from GSI only and combined PBT and GSI results. Standard deviation of the estimates is in parentheses

Population	2016 jacks			2017 CWT		
	True	GSI	PBT‐GSI	True	GSI	PBT‐GSI
Quinsam	12.0	11.7 (1.4)	11.9 (1.4)	7.4	7.0 (1.1)	7.2 (1.1)
Puntledge	3.7	3.8 (1.1)	3.7 (1.0)	0.3	0.0 (0.1)	0.3 (0.2)
Big Qualicum	14.7	14.8 (1.6)	15.0 (1.7)	5.3	5.9 (1.1)	6.2 (1.1)
Robertson	11.5	11.5 (1.4)	11.5 (1.3)	13.5	13.4 (1.4)	13.5 (1.4)
Inch	15.5	7.2 (1.2)	15.2 (1.2)	18.5	6.2 (1.2)	17.6 (1.2)
Norrish	0.2	7.2 (1.3)	0.6 (1.3)	0.0	10.7 (1.4)	0.3 (1.4)
Coldwater	0.0	0.0 (0.0)	0.0 (0.0)	0.8	0.7 (0.3)	0.8 (0.4)
Salmon	0.0	0.0 (0.0)	0.0 (0.0)	0.5	0.4 (0.3)	0.5 (0.3)
Chilliwack	34.0	34.2 (2.0)	34.1 (2.0)	0.0	0.0 (0.0)	0.0 (0.0)
Stave	3.5	4.2 (0.9)	3.6 (0.9)	0.0	0.0 (0.0)	0.0 (0.0)
Goldstream	4.7	3.5 (0.9)	4.1 (0.9)	0.0	0.0 (0.0)	0.0 (0.0)
Capilano	0.0	0.0 (0.0)	0.0 (0.0)	0.0	0.0 (0.1)	0.0 (0.0)
Nitinat	0.0	0.0 (0.1)	0.0 (0.1)	0.0	0.0 (0.0)	0.0 (0.0)
Conuma	0.0	0.0 (0.0)	0.0 (0.0)	0.0	0.0 (0.0)	0.0 (0.0)
Rosewall	0.0	0.0 (0.0)	0.0 (0.0)	0.0	0.3 (0.3)	0.2 (0.3)
Tenderfoot	0.0	0.0 (0.0)	0.0 (0.0)	0.0	0.0 (0.0)	0.0 (0.0)
Mamquam	0.0	0.0 (0.0)	0.0 (0.0)	0.0	0.0 (0.0)	0.0 (0.0)
Chehalis	0.0	0.0 (0.0)	0.0 (0.0)	0.0	0.0 (0.0)	0.2 (0.0)
Nicomekl	0.0	0.0 (0.0)	0.0 (0.0)	0.0	0.0 (0.0)	0.0 (0.0)
Serpentine	0.0	0.0 (0.0)	0.0 (0.0)	0.0	0.0 (0.0)	0.0 (0.0)

Of the 3,054 adipose fin‐clipped coho salmon samples received for potential genotyping from a central laboratory where 2017 fishery samples were processed for potential CWT recovery, genotypes were obtained for 2,533 (82.9%) of the individuals processed. There were 750 CWTs recovered from 3,054 heads or snouts examined, and genotypes were obtained from 622 (82.9%) of the individuals that carried a CWT. Genotypes of coho salmon without CWTs but sent from the CWT head recovery laboratory were obtained from 1,911 of 2,304 individuals (82.9%). Release information associated with the CWT was available for 620 of the 622 CWTs associated with genotyped individuals, and these 620 individuals constituted a sample of known origin. There were individuals from 20 regions or CUs present in this sample, and for these 20 regions or CUs, the average error of estimation utilizing only GSI was 0.9% per region or CU and 0.9% for both PBT and GSI combined (Table 2). CWTs were recovered originating from seven populations where it was possible to incorporate both GSI and PBT in estimation of stock composition. For these seven populations, the average error in estimated stock compositions per population was 2.0% with GSI and 0.3% with PBT‐GSI (Table 4). There was an average 0.0% error for the 13 populations with no representation in the sample with utilization of either GSI or PBT‐GSI (Table 4). Similar to the sample of jacks, PBT when combined with GSI produced more accurate estimates of population‐specific stock composition than available with only GSI.

### 3.2 Evaluation of 2016 fishery sampling

As broodstock sampling at selected hatcheries in southern BC did not commence until 2014, it was not possible to obtain PBT assignments for any fishery samples collected in 2016. However, GSI analysis of individuals derived from recreational fishery samples in the Strait of Georgia, Juan de Fuca Strait, and off the west coast of Vancouver Island in southern BC indicated that there was virtually no contribution from regions or CUs north of Vancouver Island (Supporting Information Table S2). The only exception was in the northwest coast of Vancouver Island, both inshore and offshore, where up to 12% of the sample was identified as originating from northern and central BC CUs. US‐origin coho salmon were estimated to have contributed approximately 70% of summer recreational samples derived from offshore fisheries along the west coast of Vancouver Island, as well as in the entrance to Juan de Fuca Strait (A21, A121). In more inshore areas along the west coast of Vancouver Island, coho salmon from local CUs were the dominant contributors to the fishery. For example, the west coast of Vancouver Island CU contributed approximately 80% of the 39‐individual August sample from the inshore southwestern Vancouver Island region. In the Strait of Georgia, local CUs were again the main contributors to the recreational fishery during the summer, with the Howe Sound–Burrard Inlet CU contributing up to 40% in some samples, the east coast of Vancouver Island CU up to approximately 30%, and the lower Fraser River CU up to approximately 40%. While the lower Fraser River CU was an important contributor to the recreational fishery in the Strait of Georgia and Juan de Fuca Strait, its contribution to fisheries off the west coast of Vancouver Island was limited. The Nooksack River region in northern Washington State was also identified as a consistent contributor to recreational fishery samples in the Strait of Georgia, in some cases contributing up to 20% of the sample analyzed (Supporting Information Table S2).

Stock compositions were available from two test fisheries, one a troll test fishery near Brooks Peninsula by northwestern Vancouver Island and the other a gillnet test fishery in upper Johnstone Strait. In the Brooks Peninsula fishery, Vancouver Island CUs comprised 40%–60% of the samples, with a minor contribution of Fraser River CUs (<10%). The five interior Fraser River CUs of conservation concern comprised 0.0%–3.3% of the samples, whereas the more abundant lower Fraser River CUs comprised only 0.0%–5.5% of the samples. Individuals from Fraser River CUs were not detected during July in Area 127, the offshore portion of northern northwestern Vancouver Island, but they were detected in Area 126, the more southerly offshore portion of northwestern Vancouver Island. In the Johnstone Strait (Round Island) test fishery, individuals from Fraser River CUs were scarce, but the five interior Fraser River CUs comprised 3.0%–3.5% of the samples, whereas the lower Fraser River CUs comprised only 0.0%–6.5% of the samples. The major contributors to the samples originated from the east coast of Vancouver Island (24%–45%) and Howe Sound–Burrard Inlet CUs (9%–11%), as well as coastal Washington (8%–15%) (Supporting Information Table S2).

The final fishery sampled was a demonstration commercial troll fishery in central coastal BC (A6 and A7). Contributions from local CUs were higher in samples from more inshore areas (A7‐7, August 2, 12% southern CUs and regions), with contributions from southern CUs higher in the more seaward areas (A6‐9, September 8, 62% southern CUs and regions). Like the west coast of Vancouver Island, migrating stocks were more likely to be found in more seaward locations.

### 3.3 Evaluation of 2017 fishery sampling

One source of fishery samples was direct fishery sampling of individuals (both adipose fin‐clipped and unclipped) where the focus was not associated with potential CWT recovery. In this case, sampling of unclipped individuals from the northern (Area F) troll fishery indicated that initially (July 10–12) northern and central BC CUs contributed 23% to the catch, southern BC CUs 53%, and southern U.S. regions 23% (Supporting Information Table S3). Stock composition changed markedly in samples collected July 13–18, with Alaska contributing 12%, northern and central BC CUs contributing 72%, and southern BC CUs contributing 16%, with no southern U.S. contribution. Samples collected during July 22–28 revealed a dominant contribution from northern and central BC CUs (96%), and CUs from this region contributed by far the largest portion of the catch in subsequent samples, with southern BC CUs and southern U.S. regions largely absent (Supporting Information Table S3). The average contribution to the weekly samples from July 22 onwards was 17% southeast Alaska, 16% Haida Gwaii–Graham Island Lowlands CU, 15% Douglas Channel–Kitimat Arm CU, 12% Hecate Strait Mainland CU, 8% Northern Coastal Streams CU, and 7% Lower Nass CU. Not unexpectedly, sampling of only clipped individuals from the fishery indicated very substantial differences in stock composition between clipped and unclipped individuals. The average contribution to the weekly samples of clipped individuals from July 19 onwards was 32% coastal Washington, 11% mid‐Puget Sound, 10% west Vancouver Island CU, and 5% for each of northern Puget Sound and Juan de Fuca Strait (Supporting Information Table S4). However, the observed clip rate in the fishery was 1.6%, so the catch was dominated by unclipped individuals. The September 2–8 sample from the Haida Gwaii recreational fishery near Langara displayed a stock composition of 42% Haida Gwaii–Graham Island Lowlands CU, 15% Hecate Strait Mainland CU, 10% Lower Skeena CU, and 8% Northern Coastal Streams CU, similar to that observed in the troll fishery, with the local CU (Haida Gwaii–Graham Island Lowlands) in higher proportions as would be expected in a more inshore fishery (Supporting Information Table S3).

Direct sampling of the central coast troll fishery revealed that, on average, the Douglas Channel–Kitimat Arm CU was the largest contributor to the fishery (24%), followed by the Northern Coastal Streams CU (19%), Bella Coola–Dean Rivers (18%), Southern Coastal Streams–Queen Charlotte Strait–Johnstone Strait–Southern Fjords CU (10%) (Supporting Information Table S3), with all CUs proximal to the location of the fishery. Samples from the recreational central coast fishery indicated that the Bella Coola–Dean Rivers CU was the dominant contributor to the fishery (32%), followed by the Rivers Inlet CU (19%), Southern Coastal Streams–Queen Charlotte Strait–Johnstone Strait–Southern Fjords CU (15%), and Douglas Channel–Kitimat Arm CU (7%) (Supporting Information Table S3). Given the location of the fishery, dominant contributions from these CUs were to be expected.

As noted previously, samples were also available from virtually all individuals sampled from commercial, recreational, and First Nations fisheries that would be routinely processed for CWT recovery in a year by a central laboratory. Individual heads from commercial fisheries sent to the central laboratory had been previously screened electronically for the presence of a CWT. Voluntary head samples from adipose fin‐clipped individuals from recreational fisheries were sent to the central laboratory, but many of these individuals did not contain a CWT, as there was no prior screening of these individuals. Stock composition of clipped individuals in the northern troll fishery has been previously outlined above. Stock composition in the northern recreational fishery indicated a wide geographic distribution of contributions, ranging from coastal Washington (16%), the west coast of Vancouver Island CU (10%), the upper Skeena CU (9%), southeast Alaska region (8%), mid‐Puget Sound (7%), and east coast of Vancouver Island–Strait of Georgia CU (6%) (Supporting Information Table S4). Stock composition of clipped individuals in the central coast recreational fishery was dominated by the Howe Sound–Burrard Inlet CU (27%) and east Vancouver Island–Strait of Georgia CU (25%) (Supporting Information Table S4), but given the low observed clip rate in the fishery (0.14%), there was very little catch that could be identified as hatchery origin.

Direct sampling of recreational fisheries in southern BC, which included both clipped and unclipped individuals in the sample, indicated that there could be very substantial differences in stock composition for fishery samples taken in the same month and general location between direct fishery samples and those derived from samples of clipped individuals delivered to the central processing laboratory for potential CWT recovery. For example, in the July sample from the Johnstone Strait creel survey (*N* = 204, 15% clip rate), two CUs (Southern Coastal Streams–Queen Charlotte Strait–Johnstone Strait–Southern Fjords, Homathko–Klinaklini Rivers) comprised 31% of the sample (Supporting Information Table S5). In the August sample (*N* = 62, 18% clip rate), these two CUs comprised 52% of the sample. Samples delivered to the central processing laboratory for the same time periods both displayed no contribution of these two CUs to the sample. As no adipose fin clipping occurs for populations in these CUs, contributions of these CUs to fisheries would be unknown if only clipped individuals were sampled from the fisheries. Similarly, in the WCVI recreational fishery, substantial differences in stock composition with respect to the US component were observed between direct fishery sampling and samples of clipped individuals delivered to the central processing laboratory for potential CWT recovery. For example, in the June direct sample (*N* = 116, 13% clip rate), US populations were estimated to have comprised 9.7% of the sample, whereas in the clipped‐only sample (*N* = 23), the US populations comprised 69.8% of the sample. Similarly in July, direct sampling of the creel catch (*N* = 253, 18% clip rate) indicated that US populations comprised 13.8% of the sample, but were estimated to comprise 64.3% of the clipped sample (*N* = 205) submitted for potential CWT recovery. In August, direct sampling of the creel catch (*N* = 178, 19% clip rate) indicated that US populations comprised 18.6% of the sample, but were estimated to comprise 68.5% of the clipped sample (*N* = 205) submitted for potential CWT recovery (Supporting Information Table S5). Clearly, sampling only adipose fin‐clipped individuals from a fishery cannot provide reliable estimates of stock composition from the entire fishery.

The large majority of samples from recreational fisheries that was processed by the central laboratory for potential CWT recovery was derived from fisheries in southern British Columbia. In the Johnstone Strait fishery, the east of Vancouver Island–Strait of Georgia CU comprised 31% of the seasonal sample, followed by the Lower Fraser CU (17%), Howe Sound–Burrard Inlet CU (17%), and Washington regions Skagit River (8%) and northern Puget Sound (8%) (Supporting Information Table S4). In the Strait of Georgia fishery, the Howe Sound–Burrard Inlet CU (36%), the lower Fraser River CU (32%), and the east coast of Vancouver Island–Strait of Georgia CU (12%) were the main contributors to the catch, with lesser contributions from northern Puget Sound (7%) and Skagit River (6%) (Supporting Information Table S4). In the Juan de Fuca Strait fishery, the lower Fraser River CU (25%) was the dominant contributor, with the Howe Sound–Burrard Inlet CU (9%) and east Vancouver Island–Strait of Georgia CU (8%) also contributing to the fishery. Contributions from Washington regions were also important, among them northern Puget Sound (17%), mid‐Puget Sound (11%), and south Puget Sound (10%). Given the location of the fishery, a large observed contribution (52%) of Washington‐origin coho salmon would be expected (Supporting Information Table S4). In the west coast of Vancouver Island fishery, the recreational catch was dominated by U.S.‐origin individuals, with 66% of the seasonal estimate derived from U.S. regions, primarily those in Puget Sound and coastal Washington, with some contribution from the Columbia River (5%) (Supporting Information Table S4). Not unexpectedly, the main Canadian contributor to the fishery was the west coast of Vancouver Island CU (9%), along with the east Vancouver Island–Strait of Georgia CU (7%) and lower Fraser CU (7%).

Samples were also available from freshwater fisheries, primarily in the Fraser River drainage. A sample from the mainstem Fraser River recreational fishery (*n* = 154, PBT assignments = 146) indicated a stock composition of 56% for Inch Creek/Norrish Creek, 23% Chilliwack River, 9% Chehalis River, and 9% Stave River. A sample from Nicomen Slough (*n* = 57, PBT assignments = 55), where both Norrish Creek and Inch Creek drain and the creek mouths <1 km from each other displayed a stock composition of 53% Inch Creek and 46% Norrish Creek origin. A sample from the Chilliwack River drainage (*n* = 82, PBT assignments = 79) displayed a stock composition of 100% Chilliwack River origin. Outside of the Fraser River drainage, three individuals (no CWTs) from the Capilano River fishery were identified as Capilano River in origin via PBT, and 10 individuals from the Squamish River fishery were identified as Tenderfoot Creek (part of Squamish River drainage) in origin via PBT. On Vancouver Island, two individuals (one with a CWT) from the Big Qualicum River fishery were identified as Big Qualicum River in origin via PBT, 13 individuals (six with CWTs) from the Quinsam River drainage were identified as Quinsam River in origin via PBT, and eight individuals (no CWTs) from the Somass River fishery were identified as Robertson Creek (part of Somass River drainage) in origin via PBT.

### 3.4 Comparison of CWT and PBT individual identification

As genotypes were available from 82.9% of the samples provided by the central laboratory that processed samples for CWT recovery, it was possible to make a direct comparison between the quantity of information provided by recovery of CWTs and identification of individuals via PBT. For the seven populations where CWTs were applied and the 2014 broodstock genotyped, there were 352 CWTs recovered from individuals in these seven populations sampled in Canadian fisheries. Of these individuals, 335 were sampled for potential genotyping (individuals sampled in test fisheries were not included in the samples to be genotyped), and genotypes were obtained from 86.0% (288/335) of the initial individuals processed (Table 5). PBT assignments were made for 92.0% (265/288) of the genotyped individuals, and PBT assignments were 100% accurate with respect to population of origin and age in comparison with CWTs. There were 285 additional PBT assignments made for these seven populations, which were individuals that had been adipose fin clipped but were not tagged with a CWT. For the sample provided, 335 CWTs from the seven populations were recovered, and 500 PBT assignments were made, with 49% more individual identifications through PBT than with CWTs. In addition, 680 PBT assignments were made for 13 populations where no CWTs were applied, with 367% (1,230 PBT vs. 335 CWT) more individual assignments made for the same base sample (Table 5).

**Table 5:** Total number of coded‐wire tags (CWTs) observed in 2017 Canadian fishery samples for populations where PBT could be applied, number of individuals with CWTs submitted for subsequent genetic analysis, number of individuals containing a CWT subsequently successfully genotyped, number of genotyped individuals with CWTs assigned via PBT, number of untagged individuals assigned via PBT, total number of individuals assigned via PBT, the percentage of genotyped individuals with CWTs from specific populations assigned via PBT and the subsequent accuracy (%) of assignment of coded‐wire tagged individuals for populations where the individuals were genotyped in the fishery samples and in which the 2014 broodstock was genotyped

Population	Total CWTs	Submitted CWTs	CWT individuals genotyped	PBT assignments			% CWT assigned	% accuracy of assignment
				With CWTs	No CWTs	Sum of PBT		
Quinsam	62	62	46	41	47	88	89.1	100.0
Puntledge	3	3	2	2	1	3	100.0	100.0
Big Qualicum	40	39	33	31	40	71	93.9	100.0
Robertson	117	97	84	81	186	267	96.4	100.0
Inch	117	117	115	102	6	108	88.7	100.0
Coldwater	9	6	5	5	3	8	100.0	100.0
Salmon	4	3	3	3	2	5	100.0	100.0
CWT total	352	335	288	265	285	550	92.0	100.0
Nitinat	0	0	0	0	23	23		
Conuma	0	0	0	0	10	10		
Rosewall	0	0	0	0	4	4		
Goldstream	0	0	0	0	7	7		
Tenderfoot	0	0	0	0	41	41		
Mamquam	0	0	0	0	13	13		
Capilano	0	0	0	0	135	135		
Chilliwack	0	0	0	0	253	253		
Norrish	0	0	0	0	93	93		
Chehalis	0	0	0	0	57	57		
Stave	0	0	0	0	27	27		
Nicomekl	0	0	0	0	6	6		
Serpentine	0	0	0	0	11	11		
Total			288	264	965	1,230		

### 3.5 Application of PBT to Canadian fishery samples

PBT was applied to identification of 1,230 individuals in a number of fisheries in BC from which CWTs could potentially be recovered, with the intent of combining PBT and GSI to provide high‐resolution estimates of stock composition in the samples from these fisheries (Table 6). There were also 269 additional individuals identified via PBT, primarily from direct sampling of the creel catch in recreational fisheries in southern BC, where both adipose fin‐clipped and unclipped individuals were included in the samples (Table 7). The Robertson Creek and Quinsam River populations were the most wide‐ranging populations observed, with individuals from these populations observed in northern commercial troll and recreational fisheries through to the Juan de Fuca Strait recreational fishery at the south end of Vancouver Island. In contrast, there were some major production populations, such as Capilano River and Chilliwack River, where marine fishery recoveries were mainly limited to Johnstone Strait, the Strait of Georgia, Juan de Fuca Strait, and the west coast of Vancouver Island. Identification of individuals from the Coldwater River and Salmon River, two populations of conservation concern and currently marked with CWTs, was restricted primarily to local fisheries in the Strait of Georgia and Juan de Fuca Strait. Populations from the Fraser River drainage displayed a more restricted geographic distribution in fisheries than did those from Vancouver Island. Contributions to fisheries were observed for every single population where the broodstock had been genotyped. PBT provided the first known occurrence of identification of individuals originating from the 13 non‐CWT populations in Canadian fisheries.

**Table 6:** PBT assignments by population for fisheries in BC during 2017 for samples sent to a central laboratory for potential CWT recovery. Fisheries were (a) northern troll, (b) northern sport, (c) central troll, (d) central sport, (e) Johnstone Strait sport, (f) Strait of Georgia sport, (g) Juan de Fuca Strait sport, (h) west coast Vancouver Island sport and troll, (i) Barkley Sound and Alberni Inlet sport, (j) freshwater sport, (k) all fisheries

Population	Fishery										
	1	2	3	4	5	6	7	8	9	10	11
Quinsam	4	1		6	51	4	6	2	1	13	88
Puntledge			1		2						3
Big Qualicum	1	3	2		25	21	12	5		2	71
Robertson	17	15	2		13	1	1	33	177	8	267
Inch	1				4	8	15	3		77	108
Norrish					7	18	6	3		59	93
Coldwater						2	5	1			8
Salmon						1	4				5
Nitinat		4					4	13	2		23
Conuma					2			6	2		10
Rosewall					1	3					4
Goldstream		1			2		2	2			7
Tenderfoot				3	10	8	8	1		10	41[Link]
Mamquam			1	2	3	3	4				13
Capilano				2	29	71	21	8	1	3	135
Chilliwack				1	24	39	54	14		121	253
Chehalis					9	10	18	6		14	57
Stave					4	3	5		1	14	27
Nicomekl							6				6
Serpentine					1	3	5	2			11
All populations	23	24	6	14	187	195	176	99	184	321	1,230[Link]

Includes one individual of unknown marine catch region.

**Table 7:** PBT assignments by population for fisheries in southern BC during 2017 by direct sampling. Fisheries were (a) Johnstone Strait test gillnet (b) Johnstone Strait sport, (c) Strait of Georgia sport, (d) Juan de Fuca Strait sport, (e) west coast Vancouver Island sport, (f) all fisheries

Population	Fishery					
	1	2	3	4	5	6
Quinsam	6	12	8	3	2	31
Puntledge			1			1
Big Qualicum	2	2	12	2		18
Robertson	1	2			6	9
Inch		1	9	3		13
Norrish		1	12	3		16
Coldwater		1	1			2
Salmon				1		1
Nitinat				1		1
Conuma					4	4
Rosewall			3			3
Goldstream		1	2	1	1	5
Tenderfoot	1	1	5	1		8
Mamquam	1		3			4
Capilano	1	6	54	4	2	67
Chilliwack	1	1	50	13	1	66
Chehalis			4	9		13
Stave	1		3	1		5
Nicomekl				1		1
Serpentine			1			1
All populations	14	28	168	43	16	269

### 3.6 Estimation of catch of hatchery‐origin populations

Assessment of the impact of fisheries on specific CUs or populations within CUs via genetics requires that accurate, high‐resolution estimates of stock composition of the catch are available. We obtained these estimates of stock composition with respect to CU (Supporting Information Tables S2–S5) and populations within CUs (Supporting Information Table S6) to estimate the population‐specific catch of adipose fin‐clipped individuals for the fisheries outlined in Table 8. For each fishery, the clipped catch that was kept for individual populations was estimated via seasonal stock composition estimates of the clipped catch (Supporting Information Table S6). Hatchery‐origin contributions to the kept catch for the genotyped populations were estimated to be the largest in the Strait of Georgia recreational fishery, with the Capilano River and Chilliwack River populations comprising 53% of the recreational catch (Table 8). Hatchery‐origin contributions to the kept catch were the largest for recreational fisheries in Johnstone Strait, the Strait of Georgia, Juan de Fuca Strait, and the west coast of Vancouver Island.

**Table 8:** Catch of hatchery‐origin coho salmon by population for fisheries in BC during 2017 with catch derived from PBT‐GSI for 10 fisheries in BC. Hatchery‐origin catch was estimated as (total catch) * (observed adipose fin clip rate in the catch). Population‐specific hatchery‐origin catch was estimated as (hatchery‐origin catch) * (population‐specific stock composition). Fisheries were (a) northern troll, (b) northern sport, (c) central troll, (d) central sport, (e) Johnstone Strait sport, (f) Strait of Georgia sport, (g) Juan de Fuca Strait sport, (h) west coast Vancouver Island sport and troll, (i) Barkley Sound–Alberni Inlet sport, (j) freshwater sport, (k) all fisheries. *N* is the number of individuals included in the seasonal sample for estimated stock composition of the catch

Population	1	2	3	4	5	6	7	8	9	10	11
Catch	339,623	35,100	6,448	18,180	7,833	7,636	8,121	21,083	5,823		
Clip rate (%)	1.64	1.64[Link]	0.14[Link]	0.14	40.04	83.93	83.58	71.02	21.12		
Hatchery‐origin catch	5,570	576	9	27	3,136	6,409	6,788	14,974	1,230		
*N*	768	85	9	27	192	171	374	517	213		
Quinsam	56	6	0	6	558	122	102	75	6	204	1,135
Puntledge	0	13	1	0	22	0	0	45	0	0	81
Big Qualicum	50	9	2	0	339	596	292	360	0	31	1,679
Robertson	596	59	2	0	144	25	14	1,078	1,069	123	3,110
Inch	6	0	0	0	38	192	238	90	0	924	1,488
Norrish	0	0	0	0	75	481	122	120	1	768	1,567
Coldwater	0	0	0	0	0	45	82	30	0	0	157
Salmon	0	0	0	0	0	26	61	0	0	0	87
Nitinat	156	24	0	2	0	0	61	465	15	0	723
Conuma	22	0	0	0	28	0	0	300	49	0	399
Rosewall	11	0	0	0	9	71	0	60	0	0	151
Goldstream	150	7	0	0	28	0	82	479	0	0	746
Tenderfoot	22	1	1	4	145	333	163	30	0	153	852
Mamquam	23	0	0	2	28	71	68	0	4	0	196
Capilano	78	0	0	2	345	1,904	407	300	7	46	3,089
Chilliwack	22	0	0	1	245	923	883	435	0	2,998	5,507
Chehalis	0	0	0	0	107	282	313	180	0	42	924
Stave	11	0	0	0	57	141	102	60	10	42	423
Nicomekl	0	0	0	0	6	0	102	0	0	0	108
Serpentine	6	0	0	0	10	83	81	60	0	0	240
All PBT populations	1,209	119	6	17	2,184	5,295	3,173	4,167	1,161	5,329	22,662

Adipose fin clip rate was assumed to be equivalent to that observed in the troll fishery.

Adipose fin clip rate was assumed to be equivalent to that observed in the recreational fishery.

### 3.7 Assessment of escapement

Non‐broodstock escapement samples were available from 13 populations, and PBT assignments were made for 90.4% (1,530/1,692) of the individuals genotyped. Stray rates were estimated at 0.7% (10/1,530) (Table 9). Strays were usually observed between geographically proximate populations, such as between Rosewall Creek and Puntledge River on the east coast of Vancouver Island, and between Inch Creek and Norrish Creek, both draining into Nicomen Slough as part of the Fraser River drainage. The largest stray distance observed was for four individuals estimated to have strayed from the Chehalis River to the Stave River, a distance of approximately 40 km in the Fraser River drainage.

**Table 9:** Number of adipose fin‐clipped jacks and adults successfully genotyped from 2017 non‐broodstock escapement samples, the number of individuals of each life stage subsequently assigned via PBT to either the 2014 broodstock (adults) or 2015 broodstock (jacks), number of individuals straying into escapement identified via PBT, and estimated stock composition (%) of escapement sample (jacks and adults combined). Standard deviation of stock composition is in parentheses

Population	Life stage	Number genotyped	Assigned via PBT	Strays via PBT	% stock composition
Robertson	Jack	10	9	0	100.0 (1.0)
	Adult	90	90	0	
Conuma	Adult	14	8	0	93.4 (9.3)
Big Qualicum	Jack‐Adult	100	100	0	100.0 (1.0)
Puntledge	Adult(clipped)	4	4	1[Link]	97.3 (3.4)
	Jack‐Adult(unclipped)	42	26	0	
Quinsam	Jack‐Adult	196	191	0	98.3 (1.5)
Capilano	Adult	85	68	0	100.0 (1.8)
Chilliwack	Jack	100	58	0	99.9 (0.8)
	Adult	200	183	0	
Inch	Jack	557	525	3[Link]	99.9 (0.3)
	Adult	95	90	1[Link]	
Norrish	Adult	6	4	1[Link]	100.0 (12.2)
Stave	Adult	111	98	4[Link]	95.9 (2.1)
Chehalis	Adult	75	69	0	100.0 (1.3)
Coldwater	Adult	6	6	0	100.0 (12.5)
Nicomekl	Adult	1	1	0	100.0 (1.0)
All populations		1,692	1,530	10	99.3

Rosewall Creek origin.

Norrish Creek origin.

Inch Creek origin.

Chehalis River origin.

### 3.8 Estimation of exploitation rate

Estimation of exploitation rates (ER) for populations was conducted with CWTs when available, and with genetics if escapement estimates were available. For the Quinsam River population, the ER estimated via CWTs (31%) and genetics (28%) were similar (Table 10). Larger contributions of hatchery‐origin catch and escapement estimated via CWTs than genetics were likely attributable to a portion of the production that was marked with CWTs but were not adipose fin clipped upon release. For the Puntledge River population, where only 12.8% of the juvenile production was clipped, the ER estimated via genetics was higher (32%) than via CWTs (10%) (Table 10). Catch estimates for this population via CWTs were derived from the expansion of three recovered CWTs, so each recovered CWT was equivalent to a 3% ER, with sampling variability and CWT loss during sampling potentially accounting for much of the observed difference for this population. For the Big Qualicum River population, both CWTs and genetics provided an estimate of ER of 22%. A discrepancy in ER was observed for the Robertson Creek population, with a rate of 44% from CWTs compared with 27% from genetics (Table 10). Differences were apparent in both the estimated catch and hatchery component of the escapement at Robertson Creek. At Inch Creek in the lower Fraser River, the ER estimated via CWTs was 41% and 35% via genetics (Table 10). Like the Quinsam River population, a portion of the production was marked with CWTs but was not adipose fin clipped upon release, possibly accounting for part of the difference in estimated ERs. ERs for the Salmon River and Coldwater River populations estimated via CWTs were about 6% less than those estimated via genetics, which was largely attributable to higher estimated fishery catches of these populations via genetics than was obtained via CWTs (Table 10). Both of these populations originate from CUs of conservation concern, and the ERs for these populations were among the lowest observed ERs of the seven index populations examined.

**Table 10:** Observed number of CWTs from 2017 Canadian fishery sampling, estimated 2017 catch in Canadian fisheries of hatchery‐origin adult coho salmon via CWTs, ER (%) via CWTs of adult coho salmon in Canadian fisheries, estimated 2017 escapement of hatchery‐origin adult coho salmon, observed assignments via PBT from 2017 fishery sampling, estimated catch via PBT‐GSI, ER of clipped adult coho salmon, total adult 2017 escapement, adipose fin clip rate observed in escapement, and clipped hatchery component of adult escapement for selected coho salmon populations. N/A is not applicable as CWTs were not applied to the population

Population	Coded‐wire tags				PBT‐GSI			Adipose clips		
	Catch			Escapement	Catch			Escapement		
	Obs. CWTs (adults)	Estimated catch (adults)	ER	Hatchery‐origin	Obs. PBT	Estimated catch	ER	Total adults	Clip rate	Hatchery‐origin clipped
Quinsam	62	1,323	31	3,010	88	1,135	28	3,480	82.3[Link]	2,864
Puntledge	3	142	10[Link]	1,330	3	81	32	2,397	7.1[Link]	170
Big Qualicum	40	1,365	22	4,834	71	1,679	22	6,933	84.9	5,886
Robertson (swim‐ins)[Link]	117	4,030	44	5,206	267	3,110	27	9,511	88.0	8,370
Inch	117	1,879	41	2,781	108	1,488	35	2,779	99.7	2,771
Coldwater	9	97	16	518	8	157	23	2,178	24.1	525
Salmon	4	51	10	453	5	87	16	676	65.4	442
Nitinat (swim‐ins)[Link]	N/A				23	723	33[Link]	1,497	96.1	1,439
Conuma	N/A				10	399	20[Link]	3,910	41.9	1,638
Rosewall	N/A				4	151	65[Link]	80	100.0	80
Goldstream	N/A				7	746	96	99	33.3	33
Tenderfoot	N/A				41	852	55[Link]	925	74.7	691
Capilano	N/A				135	3,089	21[Link]	12,244	96.9	11,864
Chilliwack (swim‐ins)	N/A				253	5,507	28[Link]	14,492	97.7	14,159
Chehalis (swim‐ins)	N/A				57	924	72[Link]	587	61.5	361

CWT hatchery‐origin includes unclipped tagged which do not get counted in clipped hatchery‐origin for Quinsam River.

In 2014 broodyear, 12.8% of Puntledge fry release was clipped and tagged, and the remainder was released unclipped. In 2017 escapement sampling, a CWT loss rate of 31% was observed due to sampling for otoliths and CWTs from the same individual.

Total escapement to the Stamp River plus Robertson Creek was 21,175 adults. 9,511 adults is the hatchery return, which is all that was sampled.

Total escapement to the Nitinat River was 4,883 adults. Clip rate is from swim‐ins so total adults is just swim‐ins, same as Robertson Creek.

Minor components of the escapement were not enumerated; however, the exploitation rate can be considered reasonably accurate due to the very high percentage of total return that were enumerated in the hatchery swim‐in count at Nitinat River, Capilano River, and Chilliwack River.

Significant components of the escapement were not enumerated; consequently, exploitation rate was overestimated by a large but unknown percentage.

Analogues to ERs were also determined for eight populations where no CWTs were applied. They ranged from 20% for the Conuma River population enhanced in a large hatchery on the west coast of Vancouver Island to 96% for the Goldstream River population, a population at the southern end of Vancouver Island where production is supplemented by a small volunteer‐staffed hatchery (Table 10). Estimated ERs of populations from the larger production hatcheries, like Conuma River (20%), Capilano River (21%), and Chilliwack River (28%) estimated entirely via genetics were similar to those of other large production hatcheries such as Big Qualicum River (22%), Robertson Creek (27%), and Quinsam River (28%), where ER was estimated via CWTs or genetics.

## 4 DISCUSSION

Canadian commercial and recreational fisheries for coho salmon have been severely restricted since the late 1990s, but comprehensive evaluation of the benefits derived from reduced exploitation and mass marking of hatchery production has not been possible. Large untagged hatchery populations in southern BC and lightly monitored wild populations of central and northern BC have been invisible to the CWT‐based management system. The successful application of current genetic technologies in this study, allowing accurate identification of coho salmon sampled from mixed‐stock fisheries, has enabled the first Canadian assessment of fishery impacts that is sufficiently informative for conservation‐based management as envisaged in the WSP. Coho salmon harvested in Canadian commercial and recreational fisheries were identified to Canadian CUs and American geographic regions from southeast Alaska to Oregon, confirming the utility of a PBT‐GSI approach for conservation‐based assessment of hatchery enhancement and mixed‐stock harvest on a wide geographic scale.

### 4.1 Accuracy of estimation of stock composition

One key difference between the CWT method and PBT‐GSI method as applied to salmon assessment relates to the inability of the CWT approach to provide estimates of stock composition of the catch. CWT recoveries are used to estimate total contributions from tagged populations through “expansions” to account for the CWT marking rate and proportion of the catch sampled. However, no estimation of the catch contributions from untagged populations is possible, precluding the estimation of stock composition for the entire fishery sample that includes fish from tagged and untagged populations. In contrast, the main function of a PBT‐GSI approach is to estimate the stock composition of the catch. Population‐specific catch estimates can be determined by multiplying the known catch by the population‐specific proportion estimated via PBT‐GSI. If the proportion of adipose fin‐clipped individuals in the catch is estimated, and separate estimates of stock composition for clipped and unclipped individuals available, then population‐specific estimates of catch of both clipped and unclipped portions of the catch can be determined. If the proportion of clipped individuals in the catch is unknown, then population‐specific estimates of the clipped (hatchery) and unclipped (natural) portions of the catch can still be determined if the proportion of clipped individuals in the population escapement is known, with the assumption that the same proportion of clipped individuals observed in the population escapement would be observed in the estimated population catch. Both CWTs and PBT‐GSI can be used to estimate the catch of the hatchery component of a population (likely even a wild index population) for later application in estimation of fishery exploitation rate, but only the PBT‐GSI approach can provide reliable estimates of stock composition of the unmarked populations in the fishery sample.

GSI provided the foundation for stock composition analysis in the study, and it has been demonstrated to provide reliable estimates of coho salmon stock composition for CUs or regional groups, with an average error of ≤1.0% observed in known‐origin samples. Estimates for CUs or regions that displayed higher error rates could likely be improved by increasing the number of populations in the baseline used to represent the CU or region. Reliable estimation of single‐population contributions to fishery samples was enhanced by the addition of PBT, with the average error rate observed with GSI declining by over 50% relative to that observed with GSI‐PBT. In particular, for the seven populations that each comprised <5% of the known‐origin samples, the average error with GSI was 1.36%, while that with GSI‐PBT was 0.16%. Accurate stock composition estimates of rare populations via GSI have been traditionally difficult (Reynolds & Templin, 2004; Winans et al., 2001), but if PBT can be applied in conjunction with GSI for these rare populations, then accurate estimates of stock compositions can be obtained even for these rare populations when they occur in the mixture sample. For example, in the 2017 CWT sample, three populations each comprised ≤1.0% of the sample, but the maximum error for the three populations when both a GSI and PBT approach was followed was 0.1%. This level of resolution for rare populations in samples from mixed‐stock salmon fisheries will likely be of importance in management of specific fisheries.

If 100% of a hatchery broodstock is successfully genotyped, there is an expectation that 100% of the offspring from the broodstock should be identifiable via PBT. In actual practice for the two known‐origin samples, assignment rates of 91.6% (CWT sample) and 94.8% (jack sample) were achieved. Failure to assign some individuals may be a result of incomplete sampling of the hatchery broodstock, failure to genotype successfully all samples that were provided, or genotyping error rates in either some of the broodstock individuals or offspring that precluded assignment to the correct parents. The origin of individuals not assigned was subsequently estimated via GSI, and the high levels of population‐specific accuracy (≥99.7%) for those populations where it was possible to implement both PBT and GSI for population‐specific stock composition indicated that observed assignment rates >91% via PBT were acceptable for high‐resolution stock identification analysis. Genotypes were obtained successfully from 82.9% (2,533 genotypes from 3,054 samples) of the samples provided by the central laboratory employed for potential recovery of CWTs from the samples processed from commercial, recreational, and First Nations fisheries. Genotyping success improved during the course of sample delivery, with initial tissue samples large and genotyping success of them reduced, but subsequently smaller tissue samples were affixed to the Whatman sheets, presumably drying quicker and preserving DNA quality, thus resulting in higher genotyping success. Also, the status of initial tissue quality for the samples was uncertain, as some samples delivered to the central laboratory for CWT recovery were deemed to be unsuitable for DNA sampling. For tissue samples directly obtained from the fishery, genotyping success was high for both commercial (northern troll fishery marked individuals, 98.9%, 815 genotypes from 824 samples) and recreational fisheries (central coast recreational fishery, 99.3%, 542 genotypes from 546 samples).

### 4.2 Exploitation rate

ER for a population is catch/(catch + escapement), and estimation of fishery exploitation rates is one of the key outcomes of assessments. For the CWT program, sampling of the fishery and escapement enables estimation of the number of specific tag codes observed in the two sets of samples. The number of observed tags is expanded by the marking rate to account for the associated hatchery releases not tagged, and again by the sampling rate in both the fishery and escapement in order to account for hatchery‐origin individuals in the unsampled portion of either the fishery or escapement samples. It is thus mandatory to sample both fishery and escapements in order to recover CWTs. When mass marking of hatchery fish occurs, the missing adipose fin can be used as an indication that the individual may contain a CWT, but the large majority of the clipped individuals will not contain a CWT.

In contrast, genetic‐based assessment benefits from mass marking of hatchery production, particularly with regard to escapement sampling. The proportion of hatchery‐origin fish in the escapement can be determined visually as the proportion of individuals missing the adipose fin, without any further sampling required. The broodstock and non‐broodstock escapement sampling in the current project generally indicated very low rates of straying among sampled populations (except between Inch and Norrish creeks), and thus if the escapement abundance is known or estimated, the hatchery portion of the escapement for a population can be estimated via the observed clip rate. No genotyping of non‐broodstock escapement is required in order to estimate the hatchery component of the escapement. However, if survival of different release groups is required to be evaluated, escapement sampling is required to assign individuals to parents and therefore release group.

Some differences in estimated ERs for the seven index populations were observed between those estimated via CWTs and those estimated via genetics. As noted previously, some portion of the juvenile production from some hatcheries was released with CWTs but were unclipped, resulting in an underestimation of the catch of hatchery‐origin fish if only the adipose fin clip was used to identify hatchery‐origin fish in the catch and escapement. The unclipped but tagged with CWTs portion of the release would be accounted for through the expansions of observed CWTs, and this likely accounted for the greater abundance of hatchery‐produced coho salmon in either catch or escapement estimated via CWTs for Quinsam River, Puntledge River, and Inch Creek. There were cases in which the hatchery component of the escapement was larger when estimated with adipose fin clip rate than with expanded CWT estimates for Big Qualicum River and Robertson Creek populations. In theory, some clipped hatchery production may not be associated with a CWT and thus would not be included in expansions of observed CWTs, and in practice, CWTs may be missed during escapement sampling, thereby underestimating the hatchery component. Estimated exploitation rates for the two Thompson River index populations (Salmon River and Coldwater River) representing CUs of conservation concern were about 6% higher estimated via genetics than with CWTs. The estimated hatchery component of the escapement was very similar between the two methods, with the difference in ER largely a result of higher fishery catch estimates via genetics than with CWTs. The observed number of CWTs recovered from fisheries for both populations was quite limited, six CWTs for Coldwater River population if the three CWTs recovered from a lower Fraser River test fishery were excluded, and four CWTs for the Salmon River. Interestingly, although most production from these two hatcheries is thought to be marked with CWTs, 38% (3/8) of the Coldwater River individuals identified via PBT in fishery samples were not associated with a CWT recovery, and 40% (2/5) of the Salmon River individuals in fishery samples were not associated with a CWT recovery. The CWT tag loss rate for 2014 broodyear juveniles during a retention check was about 10%, which was similar to the tag loss rate observed in the 2017 escapement sampling (clipped individual but no associated CWT; Coldwater 10% [6/61], Salmon 12% [6/51]). However, CWT tag loss can occur up to eight months after tag application for these populations (D. Willis, DFO, pers. comm.), and it may be that the higher fishery catches determined via genetics may reflect continuing tag loss for these populations. The limited number of CWTs recovered from fisheries for these two populations may result in, after expansions for marking rate and fishery sampling, a higher level of uncertainty of the catch abundance relative to that available from genetics, whereby catch is estimated as clipped fishery abundance * stock composition. Estimated population‐specific stock compositions of known‐origin samples via genetics for these two populations were highly accurate (Table 4), and as long as estimated catch abundance of clipped coho salmon in fisheries was reliable, accurate fishery catch estimates should be available via genetics.

Additional index populations for ER estimation can be developed via genetics if the hatchery broodstock is genotyped, the juvenile production is adipose fin clipped at release, fisheries are surveyed for stock composition, and escapements are sampled for the proportion of clipped individuals. Currently, three CWT index populations are located within a single CU (east Vancouver Island–Georgia Strait), and a more diverse geographic distribution of index populations may be desirable. We have outlined provisional results for eight additional populations, but it must be recognized that the ERs may be maximum exploitation rates in some cases, as there was no comprehensive sampling conducted on non‐broodstock escapement. Under these circumstances, the presence of hatchery‐produced individuals that returned to the river rather than the enhancement facility was unaccounted for in the estimation of enhanced (hatchery‐produced) abundance, and the exploitation rate would be overestimated. To use these locations as index systems, non‐broodstock escapement surveys would need to be conducted in order to provide a comprehensive assessment of hatchery‐origin escapement. Use of PBT‐GSI technology provides a simple means to develop additional index populations where some hatchery production occurs by the simple adoption of broodstock genotyping, juvenile mass marking, fishery sampling, and estimation of the escapement and its clip rate.

The estimated ER of 96% for the Goldstream River population is of note. Goldstream River‐origin individuals were identified via PBT in four fisheries (Tables 6 and 7) and five fisheries via PBT and GSI (Table 8). Estimated stock composition of the clipped portion of the northern troll fishery was 2.7% (*SD *= 0.6%), yet no individuals were identified via PBT. This fishery was estimated to have contributed about 20% of the estimated catch of the population (Table 8), and we assumed that a reliable estimate of catch was obtained. The estimated hatchery abundance of the escapement was the lowest of any of the populations surveyed (Table 10), lower than typically estimated for this population. Drainage escapement estimates for the prior three years were 2014: 675, 2015: 208, 2016: 1,838 (P. McCully, Goldstream hatchery, pers. comm.), or an average of 907 individuals per year. In 2017, fewer than 100 individuals were estimated in the escapement, the hatchery failed to obtain adequate broodstock, and given the low broodstock numbers, no broodstock sampling was conducted. The high ER value was concordant with the poor escapement observed in 2017, and although may be overestimated to some degree, likely reflected the regime experienced by the population.

In essence, for coho salmon in southern BC in which mass marking is applied and which display low stray rates among populations, estimation of fishery exploitation rates requires only genotyping of individuals in fishery samples and hatchery broodstocks. For wild populations, where there is a fence or weir in operation, small clips could be taken from potential spawners at the fence to provide the basis for genetic identification of their offspring in fishery samples (Ford, Pearsons, & Murdoch, 2015). Some portion of the smolts could be captured and adipose fin‐clipped, and subsequent sampling of fin‐clipped individuals in fishery sampling would allow for estimation of fishery catch for the wild population. The identification of fin‐clipped individuals in the escapement would be possible based on previous juvenile marking. The estimated escapement abundance and observed marking rate in the escapement would allow determination of the estimated fin‐clipped component of the escapement. With estimates of clipped catch of the wild population in fisheries and abundance of the clipped component in the escapement, exploitation rate of the wild population could be determined.

### 4.3 Conservation unit management

The current CWT system of assessment for coho salmon provides little information for management and assessment of wild populations, especially in central and northern BC where the majority of coho salmon is commercially harvested. The observed adipose fin clip rate in 2017 fisheries in the region was very low (0.14%–1.64%), and only a portion of the clipped individuals contained CWTs. In excess of 98% of the catch was unclipped, rendering the value of the CWT program to be of very limited value for management of directed fisheries (P. Katinic, DFO, pers. comm.). The PBT‐GSI approach to fishery assessment enables catch by CU to be determined for any fishery in the province and a means to implement the conservation/harvest balance that could be achieved by managing a combination of mixed‐stock ocean fisheries and potential in‐river fisheries targeting only healthy CUs (Price et al., 2017), providing substantial improvement to both CU status assessment as required by the WSP (DFO 2005) and MU fishery management. The use of PBT to identify members of hatchery or wild indicator populations and GSI to identify remaining individuals in the catch identifies the previously unknown components of the harvest when assessed with CWTs.

### 4.4 Hatchery management

A genetic method of assessment enables hatchery broodstock management and assessment of hatchery production with either harvest augmentation or conservation goals. The dramatic decline in coho salmon abundance that occurred in British Columbia during the 1990s spurred the implementation of mass marking of hatchery‐produced fish to enable mark‐selective recreational fisheries in which only hatchery‐produced fish were harvested. High levels of hatchery production were suspected to be a contributing factor to the poor survival of wild coho salmon, leading to an increased awareness of the need to manage hatchery production and assess hatchery‐wild interactions (Beamish et al., 2010). In coho salmon, mass marking enables hatchery managers to ensure the inclusion of naturally produced fish in the broodstock if desired, and removal of hatchery‐produced fish at fences or weirs in the natural environment to control the relative influences of the natural and hatchery environment on hatchery‐supplemented populations in which gene flow between the two spawning environments takes place (Mobrand et al, 2005). Moreover, mass marking combined with parentage analysis enables assessment of the reproductive success of hatchery‐produced fish that return to spawn in the natural environment (Abadia‐Cardosa, Anderson, Pearse, & Garza, 2013; Ford et al., 2015).

Whereas the CWT system is impaired by mass marking of hatchery fish, adipose fin clipping of all hatchery fish combined with genetic identification improves hatchery, as well as fishery, management and assessment. Genetic identification does not require lethal sampling, provides the sex of the sampled individual, and allows non‐lethal sampling and release of fish at all life stages if required. In contrast, recovery of CWTs requires lethal sampling, precluding the subsequent release of sampled individuals and determination of the sex in juvenile samples. Sampling for genetic analysis requires only a tissue sample (as little as a mucous swab or scale) for analysis. Whereas CWTs are of limited use in the study of hatchery‐wild interactions, the non‐lethal and simple tissue sampling has made genetic analysis of interactions commonplace in ecological studies (Ashton, Campbell, Anders, Powell, & Cain, 2016; Denson, Brenkert, Jenkins, & Darden, 2012; Sekino, Saitoh, Yamada, Hara, & Yamashita, 2005). Moreover, the hatchery pedigree that can be obtained using PBT enables direct estimation of inbreeding and outbreeding effects in hatchery production and estimation of genetic parameters such as heritability (Berejikian et al., 2017; Kozfkay et al., 2008).

### 4.5 Utility of PBT‐GSI for Coastwide Management

The necessity of maintaining a viable CWT system for salmon assessment was recognized under the original PST between Canada and the United States signed in 1985. Whereas CWT recoveries were key factors in the development of the FRAM for coho salmon, particularly in the base period of 1986–1992, the CWT recovery system was subsequently impaired by the release of adipose fin‐clipped salmon with no corresponding CWT. By 2004, the Pacific Salmon Commission (PSC) convened an expert panel to examine limitations of the CWT program for both Chinook salmon and coho salmon and to evaluate the capacity of alternative technologies to provide data to improve assessment of salmon. The panel noted that PBT would provide the equivalent of CWT recovery data, but that an empirical demonstration was needed to validate theoretical PBT results that suggested broad feasibility (PSC, 2005). With no large‐scale PBT applications developed in the intervening years, the PSC again commissioned in 2014 an evaluation of the feasibility and cost‐effectiveness of developing a coordinated coastwide tag recovery system using PBT, stipulating that a transition from the coastwide CWT system to a PBT system would make require that:

1. The PBT system generates at least the same information currently generated from the CWT system via run reconstruction (cohort) analyses of estimated recoveries from individual CWT release groups.
2. The PBT system would have long‐term annual operating costs no greater than or, ideally, substantially less than those of the existing CWT system.
3. The cost of a coastwide PBT system was *substantially* less than that of the existing CWT system or that PBT delivers additional or novel information, not provided by the existing CWT system, to inform management of fisheries for coho and Chinook salmon (PSC, 2015).

Satterthwaite et al. (2015) explored various scenarios under which PBT could be expanded to a coastwide application for both Chinook salmon and coho salmon.

The PBT‐GSI method of assessment for BC coho salmon meets the three criteria outlined by PSC (2015). The average annual number of Canadian‐origin CWTs recovered in BC commercial, recreational, and First Nations coho salmon fisheries during 2014–2016 was 391 tags, with 550 CWTs recovered in 2017 from 14 populations. The average annual number of CWTs recovered from escapements during 2014–2016 was 4,027 tags, with 2,636 CWTs recovered in 2017 from ten populations. Fishery sampling for PBT and GSI in 2017 provided genotypes for 8,006 individuals, 1,499 of which were assigned to population and broodyear with PBT. The genetic sampling therefore provided the required identification for assessment and fishery management for almost three times as many coho salmon harvested in BC than did CWT recovery. These PBT fishery recoveries were spread over 20 populations, with assignments to the largest hatchery comprising 21.7% of the total assignments, so it was not a case of a single hatchery population comprising the bulk of PBT assignments. In fact, PBT fishery assignments were observed for all populations genotyped in 2014. Direct escapement sampling for PBT in 2017 provided 1,692 genotypes from 13 populations, and the 6,739 broodstock individuals genotyped for 32 hatchery 2017 broodstocks bring the escapement sampling total to 8,431 individuals for 32 populations. The PBT‐GSI system of identification generated equivalent information relative to the Canadian CWT program for index populations of coho salmon in southern BC, as well providing information on harvest impacts incurred by hatchery and wild populations not included in the CWT index program.

We undertook no comprehensive cost comparison between the PBT‐GSI and CWT technologies in the current study, but a preliminary cost analysis can be made as follows. Approximately 820,000 CWTs were applied to offspring from the 2014 Canadian hatchery broodstocks and wild escapement, at an estimated cost of $169,000 (820,000 * $0.17 + $30,000; Beacham et al., 2008). Therefore, the cost per fish for the Canadian‐origin CWTs recovered in Canadian fisheries in 2017 was $169,000/550 = $307. For PBT‐GSI, the cost of genotyping the 6,061 individuals in the 2014 broodstock sampling was $121,220 (6,061 * $20; Beacham et al., 2008). Therefore, the 1,230 individuals from the central CWT recovery laboratory identified by PBT in 2017 Canadian fishery samples each cost $99 ($121,220/1,230). If the fish from the direct creel sampling and Johnstone Strait gillnet test fishery are included, the cost of genetic tagging per PBT recovery declined to $81 per individual ($121,220/1,499) compared with a cost of $307 per CWT. The PBT method of identification provided a substantial cost advantage for tagging per individual identified in 2017 fishery samples compared with that provided by the CWT program. Beacham et al. (2018) provided evidence that for Chinook salmon in BC, a PBT‐GSI assessment method was substantially cheaper than the existing CWT program. In this case, the cost of CWT application and recovery and reading from fishery and escapement samples was compared with the cost of genotyping broodstock, fishery, and escapement samples. Further details were outlined by Beacham et al. (2018).

Moran et al. (2018) noted that there is a common concern among some fishery managers and staff that “investigation of new technological approaches to provide data for salmon fishery management diverts monies that can be used to maintain the existing CWT program” (Pacific Salmon Commission Joint CWT Implementation Team, 2015). Increasingly however, the deficiencies provided by the CWT‐based FRAM model for both Chinook (Moran et al., 2018) and coho salmon are revealed by application of PBT‐GSI technologies. Additionally, many GSI projects are already routinely conducted because CWTs do not provide adequate information for fisheries management decisions and assessment (e.g., Beacham et al., 2008; Bellinger et al., 2015; Satterthwaite et al., 2014). The current dissatisfaction of Canadian managers of northern and central coastal coho salmon fisheries and calls for better assessment tools for the management of mixed‐stock and in‐river fisheries in northern BC (Price et al., 2017) also highlight the need for an improved management regime. Additional cost savings may accrue from implementation of a PBT‐GSI management system, as GSI projects currently conducted on an ancillary basis to the CWT program are merged into routine fishery sampling, avoiding duplication of effort and expense.

The strongest benefits of a PBT‐GSI management system come from the additional information that it can provide, not only for improved fishery management but also for wild population conservation and management of enhancement programs. Currently, few wild populations are marked with CWTs in BC, and they are assumed to be reliable proxies for coho salmon populations over large geographic regions that may encompass multiple CUs. For the first time, analysis of northern and central coast fisheries in this study enabled comprehensive determination of fishery impacts on central/northern river systems and their constituent CUs. Similarly, contributions to recreational fisheries from all enhancement programs of southern BC were identified for the first time. Coho salmon from un‐CWTed enhancement program populations in southern BC contributed 65% of the catch in southern BC recreational fisheries in 2017, and untagged wild populations from the mainland inlets forming the eastern border of Johnstone and Georgia straits also made substantial contributions. Moreover, stray rates among hatchery‐influenced coho salmon populations were observed to be low, with important exceptions being the Inch Creek and Norrish River populations reared at Inch Creek Hatchery, in which reciprocal straying was identified. Interestingly, a lower rate of reciprocal straying was identified between the Chehalis and Stave River populations, populations reared at different hatcheries and at distal locations in the lower Fraser drainage.

Much of the increased utility of the PBT‐GSI methodology results from the fact that complete broodstock sampling ensures close to a 100% mark rate for juveniles. This rate compares very favorably with the current marking rate of approximately 10% for CWTs at selected hatcheries. The reduced cost per PBT‐tagged fish recovered versus CWT‐tagged fish stems from the high PBT mark rate, and the ability to identify fish from all hatchery facilities enables complete catch analysis through the representative sampling of both adipose‐clipped and unclipped individuals.

### 4.6 Future developments

The current study has demonstrated the PBT‐GSI capability to identify BC‐origin coho salmon to specific Canadian hatcheries and CUs, provoking consideration of replacement of the current CWT system for coho salmon assessment in BC with a PBT‐GSI based approach. The 304‐SNP panel used in the current study to genotype the 2014 coho salmon broodstocks and their jack returns at selected hatcheries (Beacham et al., 2017), and for genotyping samples from coho salmon fisheries in 2016 and 2017 has since been upgraded. A 492‐SNP panel now exists, with additional loci originating from research conducted in a Genome Canada large‐scale applied research project, and has been used to genotype the 2016 and 2017 coho salmon broodstocks at an expanded number of hatcheries. It is anticipated that this enhanced SNP panel and the increased number of facilities at which broodstock genotyping has occurred will provide improved stock composition results relative to those of the current study when applied to coho salmon fishery samples in 2019. If Canada were to implement a GSI‐PBT method of assessment for coho salmon in place of the CWT program, then complete assessment of exploitation rates for Canadian populations would require genetic analysis of samples from American fisheries if CWTs are retained as the assessment tool for American coho salmon populations and fisheries. Should a GSI‐PBT method of analysis be deemed practical for American assessment purposes, it is conceivable that a coastwide GSI‐PBT assessment method could be implemented for coho salmon fisheries.

## 5 SUMMARY

This study has demonstrated the potential for implementation of a comprehensive PBT‐GSI methodology for management and assessment of coho salmon in British Columbia that will remedy noted deficiencies of the current CWT‐based management system. Most importantly, the genetic technology provides an immediate tool for identification of coho salmon to CU, a requirement for implementation of management of wild populations as mandated by the WSP for Pacific salmon, and a task that would be prohibitively expensive using CWTs. Moreover, the PBT‐GSI technology benefits from the mass marking of hatchery‐produced salmon, thereby facilitating improved hatchery broodstock management, monitoring of wild‐enhanced fish interactions, and the evaluation of hatchery contributions to harvest. The ability to identify readily hatchery‐produced salmon has been recognized as an imperative for managing the risks and assessing the benefits of hatchery production of salmonids at the domestic, bilateral, and international levels (Ruggerone & Irvine, 2018). In Canada, extensive coho salmon conservation and enhancement efforts conducted for two decades require comprehensive evaluation and possible modification that cannot be achieved under the current management system. The genetic methodology developed in this study provides an opportunity for conservation‐based management of Canadian coho salmon in which the economic benefit of hatchery production can be reaped without the imposition of undue and unknown risk to wild populations.

## 6 Data Archiving

Multi‐locus genotypes for all sampled jacks, as well as individuals in fishery and escapement samples, are available at DRYAD doi identified as: Data from: Comparison of coded‐wire tagging with parentage‐based tagging and genetic stock identification in a large‐scale coho salmon fisheries application in British Columbia, Canada. Journal: Evolutionary Applications. https://doi.org/10.5061/dryad.6c31bf2 Data files: 2016_Coho_Jacks_rubias. 2016‐17_Coho_Fishery_Samples_rubias, 2017_Coho_Escapement_rubias.
