# Supplementary material for: Comparison of coded‐wire tagging with parentage‐based tagging and genetic stock identification in a large‐scale coho salmon fisheries application in British Columbia, Canada
Source: Evol Appl. 2018 Oct 11;12(2):230–54. doi: 10.1111/eva.12711 (PMC6346672; doi:10.1111/eva.12711)
Supplement: Supplementary file 1 [file EVA-12-230-s001.docx]

Supplementary Table 1. Coho salmon spawning locations, sample collection years, and total number of fish sampled for 267 populations in 49 geographic areas or Conservation Units ranging from southeast Alaska to Oregon. N is the number of fish genotyped in the population.

| Region/Conservation Unit | Population | Years | Number |
| --- | --- | --- | --- |
| Southeast Alaska | Berners River | 2000 | 96 |
|  | Ford Arm Lake | 2000 | 95 |
|  | Gastineau Channel | 1997 | 90 |
|  | Hidden Falls | 1997 | 85 |
|  | Hugh Smith Lake | 2000 | 101 |
|  | Indian Creek | 1996 | 148 |
|  | Margaret Creek | 1997 | 66 |
|  | Reflection Lake | 1996 | 18 |
|  | Whitman Lake | 1997 | 90 |
|  | Karta River | 1986 | 11 |
| Alsek River | Klukshu River | 2003 | 96 |
| Lower Stikine | Scud River | 2000 | 40 |
| Lower Nass | Tseax River | 1995, 1996 | 95 |
|  | Zolzap Creek | 2001 | 96 |
| Upper Nass | Meziadin River | 1995, 1996 | 93 |
| Portland Sound-Observatory Inlet-Portland Canal | Lachmach River | 2002 | 101 |
| Skeena Estuary | Diana Creek | 2015 | 13 |
|  | Oona River | 2005 | 102 |
|  | Silver Creek | 2015 | 17 |
| Lower Skeena | Gitnadoix River | 2003 | 27 |
|  | Kasiks River | 2003, 2005 | 44 |
|  | Kitsumkalum River | 1998 | 74 |
|  | Zymacord River | 2015, 2016, 2017 | 48 |
| Middle Skeena | Bulkley River | 1998 | 96 |
|  | Morice River | 1998, 1999 | 78 |
|  | Toboggan Creek | 1998 | 95 |
|  | Kitwanga River | 2014, 2017 | 92 |
| Upper Skeena | Damshilgwit Creek | 2004 | 96 |
|  | Slamgeesh River | 2000, 2005 | 96 |
|  | Sustut River | 2003 | 52 |
| Haida Gwaii-Graham Island Lowlands | Awun | 1997 | 19 |
|  | Chown Brook | 2001 | 81 |
|  | Datlamen Creek | 2001, 2002 | 73 |
|  | Mamin River | 2001 | 25 |
|  | McClinton Creek | 2001 | 29 |
|  | Sangan River | 1997 | 96 |
|  | Tlell River | 2005 | 48 |
|  | Yakoun River | 1997 | 96 |
| Haida Gwaii-East | Copper Creek | 1995 | 74 |
|  | Deena Creek | 2001 | 47 |
|  | Honna River | 2001 | 39 |
|  | Pallant Creek | 1992, 1997 | 94 |
|  | Tasu Creek | 1998 | 22 |
| Haida Gwaii-West | Coates Creek | 1999 | 23 |
|  | Loon Lake Creek | 2001 | 32 |
|  | Mercer Creek | 2001 | 59 |
|  | Tasu Creek | 1998 | 22 |
| Northern Coastal Streams | Aaltanhash River | 2002, 2004 | 45 |
|  | Brim River | 2001, 2005 | 29 |
|  | Canoona River | 2001, 2002, 2004 | 34 |
|  | Drake Creek | 2001 | 96 |
|  | Evelyn Creek | 2001-2005 | 93 |
|  | Hartley Bay Creek | 2002, 2015 | 274 |
|  | Hugh Creek | 2001-2005 | 93 |
|  | Khutze River | 2001, 2002, 2004 | 37 |
|  | Kiltuish River | 2001-2005 | 53 |
|  | Kemano River | 2001-2002 | 11 |
|  | Kiskosh Creek | 2002, 2004, 2005 | 37 |
|  | Martin River | 2001 | 96 |
|  | Paril River | 2002, 2004 | 96 |
|  | Quartcha Creek | 2001, 2004 | 43 |
|  | Quatlena River | 2001 | 75 |
|  | Roscoe Creek | 2001, 2005 | 25 |
|  | Wahoo Creek | 2001, 2005 | 17 |
| Hecate Strait Mainland | Kitasoo Creek | 2003 | 58 |
|  | McLaughlin Bay Creek | 1998 | 96 |
|  | Quaal River | 2006 | 96 |
|  | Shaw Creek | 2001 | 51 |
|  | Tankeeah River | 2003 | 38 |
|  | Tyler Creek | 2001, 2002, 2004 | 50 |
|  | Arnoup Creek | 2001, 2002, 2004 | 21 |
| Mussel-Kynoch | Kainet Creek | 2001 | 95 |
| Douglas Channel-Kitimat Arm | Bella Bella River | 2001 | 96 |
|  | Gilttoyees Creek | 2001-2004 | 51 |
|  | Kitimat River | 1996, 1997, 2015 | 315 |
|  | L_Wedeene River | 2005 | 15 |
|  | Foch Creek | 2002, 2003 | 14 |
| Bella Coola-Dean Rivers | Atnarko River | 2000 | 21 |
|  | Hagensborg Slough | 1998 | 19 |
|  | Jenny Inlet West Creek | 2000, 2001 | 53 |
|  | Necleetsconnay River | 2000 | 30 |
|  | Salloomt River | 1996, 2000 | 91 |
|  | Snootli Creek | 1996, 1998 | 57 |
|  | Thorsen Creek | 1996, 2000 | 79 |
| Rivers Inlet | Johnston Creek | 2007 | 29 |
|  | Genesee River | 2005 | 12 |
|  | Kilbella River | 2015 | 21 |
|  | Neechanze River | 2006 | 99 |
|  | Sheemahant River | 1998 | 68 |
| Smith Inlet | Docee River | 2001, 2010 | 96 |
|  | Nekite River | 2004, 2008 | 94 |
|  | Smokehouse Creek | 2015 | 19 |
| Southern Coastal Streams-Queen Charlotte Strait-Johnstone Strait-Southern Fjords | Ahnuhati River | 2015 | 61 |
|  | Heydon Creek | 2001, 2003 | 96 |
|  | Kakweiken River | 2015 | 29 |
|  | Phillips River | 1996, 2000, 2004, 2006 | 136 |
|  | Seymour River | 2015 | 44 |
|  | Wakeman River | 2015 | 38 |
|  | Waump Creek | 2002, 2003, 2015 | 30 |
| Homathko-Klinaklini Rivers | Homathko River | 2009 | 87 |
| Georgia Strait Mainland | Myrtle Creek | 2001 | 28 |
|  | Sliammon River | 1996-1998, 2016 | 134 |
| Howe Sound-Burrard Inlet | Capilano River | 2014-2017 | 2,980 |
|  | Cheakamus River | 2012 | 13 |
|  | Chapman Creek | 1999, 2015 | 71 |
|  | Mamquam River | 2014-2016 | 184 |
|  | Salish Creek | 2011-2016 | 62 |
|  | Seymour River | 2015, 2016 | 294 |
|  | Shovelnose Creek | 2005-2007, 2012 | 101 |
|  | Tenderfoot Creek | 2014-2016 | 580 |
| East Vancouver Island-Georgia Strait | Big Qualicum River | 2014-2017 | 1,826 |
|  | Chase River | 2000 | 94 |
|  | Coal Creek | 2014, 2015 | 20 |
|  | Cowichan River | 1998 | 92 |
|  | French Creek | 2015, 2017 | 72 |
|  | Goldstream River | 2014-2016 | 511 |
|  | Nanaimo River | 1996-1999, 2016 | 181 |
|  | Puntledge River | 2014-2017 | 2,403 |
|  | Quinsam River | 2014-2017 | 2,264 |
|  | Rosewall Creek | 2014-2017 | 388 |
|  | Roy Creek | 2002, 2016, 2017 | 114 |
|  | Sooke River | 1998 | 44 |
|  | Trent River | 2015 | 48 |
|  | Village Bay Creek | 2003 | 10 |
| East Vancouver Island-Johnstone Strait-Southern Fjords | Nathan Creek | 2005 | 22 |
|  | Nimpkish River | 1996, 2004 | 49 |
|  | Sorenson Creek | 2003 | 72 |
| Nahwitti Lowland | Cluxewe River | 1996, 1997, 2017 | 145 |
|  | Glenlion River | 1996-1998 | 97 |
|  | Goodspeed River | 2004 | 30 |
|  | Keogh River | 2004, 2008 | 86 |
|  | Nahwitti River | 1998-2000 | 61 |
|  | Quatse River | 1999-2000, 2017 | 149 |
|  | Quatsese River | 1998 | 52 |
|  | Stephens Creek | 1998-2000 | 117 |
|  | Tsulquate River | 1999 | 64 |
|  | Washlawlis Creek | 1998, 2000 | 93 |
|  | Waukwaas Creek | 1996-1998, 2017 | 104 |
| West Vancouver Island | Conuma River | 2014-2017 | 536 |
|  | Kennedy River | 2000 | 95 |
|  | Robertson Creek | 2014-2017 | 1,087 |
|  | Thornton Creek | 2000 | 96 |
| Clayoquot | Cypre River | 2000, 2017 | 142 |
|  | Kootowis Creek | 2000, 2017 | 104 |
|  | Tranquil Creek | 2001 | 96 |
| Juan de Fuca-Pachena | Nitinat River | 2014-2017 | 1,997 |
|  | Pachena River | 1997 | 44 |
|  | Sarita River | 1997 | 74 |
| Lower Fraser | Alouette River | 2016 | 58 |
|  | Alouette River -south | 2001, 2005 | 108 |
|  | Chehalis River | 2014- 2017 | 1,738 |
|  | Chilliwack River | 2014-2017 | 3,540 |
|  | Chilqua Creek | 2001, 2004, 2012 | 102 |
|  | Coghlan Creek | 1998, 2005, 2008 | 31 |
|  | Coquitlam River | 2015, 2016 | 107 |
|  | Hicks Creek | 2012, 2013 | 100 |
|  | Inch Creek | 2006, 2009, 2012, 2014-2017 | 1,422 |
|  | Kanaka Creek | 2005, 2015- 2017 | 162 |
|  | Nathan Creek | 2001, 2004, 2006, 2008 | 79 |
|  | Nicomen Slough | 1996-1997 | 24 |
|  | Norrish Creek | 2001, 2014-2017 | 467 |
|  | Peach Creek | 2010 | 16 |
|  | Pitt River (upper) | 1995, 1998 | 77 |
|  | Post Creek | 2004-2006, 2009, 2011, 2012 | 54 |
|  | Salmon River | 1998, 2004, 2008, 2009, 2012, 2013 | 77 |
|  | Siddle Creek | 2001, 2004, 2011, 2012 | 87 |
|  | Silverdale Creek | 2004, 2007, 2009, 2010, 2012-2014 | 106 |
|  | Stave River | 2014-2017 | 554 |
|  | Street Creek | 2004, 2012 | 14 |
|  | West Creek | 2001 | 17 |
|  | Whonnock | 2001, 2004, 2005, 2009, 2012 | 72 |
|  | Worth Creek | 2001, 2009, 2010, 2012 | 130 |
| Lillooet | Birkenhead River | 2004 | 51 |
|  | Green River | 2004 | 11 |
|  | Poole Creek | 2003, 2004, 2008 | 70 |
|  | Salmon Creek | 2004 | 20 |
|  | Sampson Creek | 2004 | 13 |
|  | Upper_Birkenhead River | 2004 | 101 |
| Fraser Canyon | Kawkawa Creek | 2004, 2009, 2010, 2012 | 107 |
|  | Nahatlatch River | 2000, 2010 | 70 |
| Interior Fraser | Chilko River | 2008 | 95 |
|  | Gates Creek | 2001, 2010 | 82 |
|  | McKinley Creek | 1998, 2011 | 83 |
|  | Seton River | 2001, 2011 | 31 |
| Lower Thompson | Bonaparte River | 2002 | 99 |
|  | Coldwater River | 2014-2017 | 240 |
| North Thompson | Albreda River | 2005, 2006, 2008, 2010 | 60 |
|  | Avola Creek | 1998, 1999, 2001, 2002, 2009, 2010 | 67 |
|  | Barriere River | 2001, 2002, 2005-2008, 2012 | 99 |
|  | Barriere River_East | 2001-2003, 2005, 2007, 2010 | 29 |
|  | Birch_Island_Channel | 2001, 2012 | 101 |
|  | Blue River | 2001, 2002, 2004, 2010 | 53 |
|  | Cedar Creek | 2001, 2002, 2009, 2010 | 14 |
|  | Cook Creek_ | 2001, 2002, 2005, 2007, 2008 | 35 |
|  | Dunn Creek | 1997, 1998 | 60 |
|  | Fennell Creek | 2005-2011 | 46 |
|  | Louis Creek | 2001, 2007, 2012 | 104 |
|  | Lyon Creek | 2002, 2010, 2011 | 101 |
|  | Mann Creek | 1997, 2002, 2004 | 13 |
|  | Miledge Creek | 2002, 2004, 2009 | 11 |
|  | Pig_Channel | 2002, 2010, 2012 | 80 |
|  | Raft River | 2001, 2004, 2006, 2009, 2010 | 104 |
|  | Reg Christie Creek | 1998, 2000-2003, 2005 | 73 |
|  | Tumtum Creek | 1998, 2004, 2006-2010 | 18 |
| South Thompson | Bessette Creek | 1997-1999, 2001, 2002, 2013 | 131 |
|  | Cayenne Creek | 2006, 2009, 2012, 2013 | 12 |
|  | Creighton Creek | 2002, 2012, 2013 | 14 |
|  | Danforth Creek | 1997, 1998, 2001, 2004, 2008, 2011 | 40 |
|  | Duteau Creek | 1999, 2001, 2002 | 66 |
|  | Eagle River | 2015, 2016 | 176 |
|  | Harbour Creek | 2002, 2004, 2007, 2009- 2013 | 92 |
|  | Harris Creek | 2001, 2011, 2013 | 93 |
|  | Ireland Creek | 2001-2004, 2008 | 26 |
|  | Lang Channel | 1998, 2002 | 73 |
|  | McNomee Creek | 1999-2004, 2006-2008, 2011-2013 | 63 |
|  | Momich River | 1998, 1999, 2003, 2006, 2012, 2013 | 87 |
|  | Salmon River | 2008, 2010, 2014, 2015 | 138 |
|  | Senn Creek | 2001, 2010, 2011 | 81 |
|  | Shuswap River_Middle | 1999 | 96 |
|  | Sinmax Creek | 2000, 2006, 2008, 2012, 2013 | 94 |
|  | Wap Creek | 2002, 2004-2006, 2008 | 81 |
| Boundary Bay | Little_Campbell River | 2013 | 24 |
|  | Nicomekl River | 2014-2017 | 275 |
|  | Serpentine River | 2014-2017 | 204 |
| Nooksack River | Nooksack_Hatchery | 1996 | 97 |
|  | Nooksack River South Fork | 2007 | 99 |
| Skagit River | Jones Creek | 2003 | 98 |
|  | Marblemount Hatchery | 1996 | 87 |
|  | Red Cabin Creek | 2003 | 15 |
|  | Sorensen Creek | 2003 | 77 |
| Northern Puget Sound | Grizzly River | 1996 | 68 |
|  | Snohimish River | 1996 | 73 |
|  | Skykomish River | 2007 | 96 |
|  | Stillaguamish River | 2007 | 53 |
| Mid-Puget Sound | Issaquah Creek | 2007 | 158 |
|  | Minter Creek | 1996 | 75 |
| Southern Puget Sound | Nisqually River | 1996 | 49 |
|  | Puyallup River | 2007 | 100 |
|  | White River | 2007 | 100 |
| Juan de Fuca Strait | Dungeness Hatchery | 1996 | 66 |
|  | Elwha River | 1996 | 79 |
| Hood Canal | Big Beef Creek | 1998 | 24 |
|  | Dewatto River | 1996 | 63 |
|  | Quilcene River | 2007 | 80 |
| Coastal Washington | Bingham Creek Hatchery | 1996 | 95 |
|  | Clearwater Creek | 1996 | 39 |
|  | Queets River | 1996 | 79 |
|  | Quillayute River | 1996 | 41 |
|  | Shale Hatchery | 1996 | 25 |
|  | Willapa River | 1996 | 61 |
| Columbia River | Bing Creek Hatchery | 2002 | 73 |
|  | Bonneville Hatchery | 2002 | 92 |
|  | Clackamus River | 1998 | 96 |
|  | Cowlitz River Hatchery | 1996 | 73 |
|  | Eagle Creek Hatchery | 2001 | 93 |
|  | Lewis River Hatchery | 1996 | 83 |
|  | Sandy River Hatchery | 2002 | 75 |
| Oregon | Beaver Creek | 2000, 2001 | 32 |
|  | Nehalem Hatchery | 2001 | 93 |
|  | Siletz River | 2000, 2001 | 45 |
|  | Siltcoos Lake | 2000, 2001 | 23 |
|  | Siuslaw River | 2000 | 83 |
|  | Tahkenitch Lake | 2000 | 10 |
|  | Ten Mile Lake | 2000 | 23 |
|  | Trask Hatchery | 2001 | 95 |
|  | Umpqua River | 2000, 2001 | 50 |
|  | Yaquina River | 2000, 2001 | 74 |
|  | Total |  | 40,582 |

Supplementary Table 2. Percentage stock composition by geographic region or CU of 2016 recreational fishery sampling of coho salmon in the Strait of Georgia (north and south), Johnstone Strait (JST), Strait of Georgia/Strait of Juan de Fuca (GS/JDF), southwest Vancouver Island (Areas 21, 121), southwest Vancouver Island inshore and offshore, northwest Vancouver Island inshore and offshore, Brooks Peninsula test fishery (Areas 27, 127, 126), Round Island test fishery (Area 12), and Central Coast commercial freezer troll fishery (Areas 6, 7). Standard deviation is in parentheses.

| Region/Conservation Unit | Strait of Georgia-North, recreational | | | | Strait of Georgia-South, recreational | | | | JST |
| --- | --- | --- | --- | --- | --- | --- | --- | --- | --- |
|  | June | July | August | Sept | June | July | August | Sept | June |
| Sample size | 21 | 130 | 91 | 35 | 15 | 63 | 17 | 16 | 23 |
| Southeast Alaska | 0.0 (0.9) | 0.0 (0.1) | 0.0 (0.2) | 0.0 (0.5) | 0.0 (1.2) | 0.0 (0.3) | 0.0 (1.0) | 0.0 (1.6) | 0.0 (0.8) |
| Alsek River | 0.0 (0.2) | 0.0 (0.0) | 0.0 (0.1) | 0.0 (0.0) | 0.0 (0.5) | 0.0 (0.0) | 0.0 (0.4) | 0.0 (0.2) | 0.0 (0.3) |
| Lower Stikine | 0.0 (0.3) | 0.0 (0.1) | 0.0 (0.1) | 0.0 (0.1) | 0.0 (0.4) | 0.0 (0.1) | 0.0 (0.3) | 0.0 (0.3) | 0.0 (0.9) |
| Lower Nass | 0.0 (0.6) | 0.0 (0.1) | 0.0 (0.1) | 0.0 (0.2) | 0.0 (0.4) | 0.4 (0.9) | 0.0 (0.4) | 0.0 (0.5) | 0.0 (0.2) |
| Upper Nass | 0.0 (0.6) | 0.0 (0.0) | 0.0 (0.2) | 0.0 (0.2) | 0.0 (0.3) | 0.0 (0.1) | 0.0 (0.1) | 0.0 (0.4) | 0.0 (0.2) |
| Portland Sound-Observatory Inlet-Portland Canal | 0.0 (0.4) | 0.0 (0.1) | 0.0 (0.1) | 0.0 (0.2) | 0.0 (0.5) | 0.0 (0.1) | 0.0 (0.2) | 0.0 (0.1) | 0.0 (0.1) |
| Skeena Estuary | 0.0 (0.5) | 0.0 (0.1) | 0.0 (0.1) | 0.0 (0.2) | 0.0 (1.0) | 0.0 (0.2) | 0.0 (0.7) | 0.0 (0.4) | 0.0 (0.4) |
| Lower Skeena | 0.0 (0.6) | 0.0 (0.1) | 0.0 (0.1) | 0.0 (0.5) | 0.0 (0.8) | 0.0 (0.3) | 0.0 (0.6) | 0.0 (0.9) | 0.0 (0.4) |
| Middle Skeena | 0.0 (0.3) | 0.0 (0.1) | 0.0 (0.1) | 0.0 (0.5) | 0.0 (0.8) | 0.0 (0.2) | 0.0 (0.9) | 0.0 (0.3) | 0.0 (0.4) |
| Upper Skeena | 0.0 (0.3) | 0.0 (0.1) | 0.0 (0.2) | 0.0 (0.2) | 0.0 (0.7) | 0.0 (0.1) | 0.0 (0.7) | 0.0 (0.7) | 0.0 (0.4) |
| Haida Gwaii-Graham Island Lowlands | 0.0 (0.7) | 0.0 (0.2) | 0.0 (0.3) | 0.0 (0.6) | 0.0 (1.1) | 0.0 (0.2) | 0.0 (0.8) | 0.0 (1.1) | 0.0 (0.5) |
| Haida Gwaii-East | 0.0 (0.8) | 0.0 (0.1) | 0.0 (0.1) | 0.0 (0.3) | 0.0 (0.9) | 0.0 (0.2) | 0.0 (0.6) | 0.0 (0.8) | 0.0 (0.4) |
| Haida Gwaii-West | 0.0 (0.5) | 0.0 (0.1) | 0.0 (0.2) | 0.0 (0.4) | 0.0 (0.8) | 0.0 (0.1) | 0.0 (0.8) | 0.0 (0.6) | 0.0 (0.3) |
| Northern Coastal Streams | 0.0 (1.1) | 0.0 (0.2) | 0.0 (0.3) | 0.0 (0.9) | 0.0 (1.2) | 0.0 (0.4) | 0.0 (1.6) | 0.0 (1.2) | 0.0 (1.0) |
| Hecate Strait Mainland | 0.0 (0.7) | 0.0 (0.1) | 0.0 (0.2) | 0.0 (0.5) | 0.0 (1.2) | 0.0 (0.3) | 0.0 (0.9) | 0.0 (1.0) | 0.0 (0.5) |
| Mussel-Kynoch | 0.0 (0.1) | 0.0 (0.0) | 0.0 (0.0) | 0.0 (0.1) | 0.0 (0.3) | 0.0 (0.2) | 0.0 (0.2) | 0.0 (0.5) | 0.0 (0.3) |
| Douglas Channel-Kitimat Arm | 0.0 (0.4) | 0.0 (0.1) | 0.0 (0.1) | 0.1 (1.2) | 0.0 (1.0) | 0.0 (0.3) | 0.0 (0.7) | 0.0 (0.7) | 0.0 (0.5) |
| Bella Coola-Dean Rivers | 0.0 (0.6) | 0.0 (0.1) | 0.0 (0.4) | 0.5 (1.5) | 0.0 (0.7) | 0.0 (0.4) | 0.0 (1.0) | 0.4 (1.2) | 0.0 (0.7) |
| Rivers Inlet | 0.0 (0.4) | 0.0 (0.1) | 0.0 (0.2) | 0.1 (0.4) | 0.0 (1.0) | 0.0 (0.2) | 0.0 (0.5) | 0.1 (0.4) | 0.0 (0.6) |
| Smith Inlet | 0.0 (0.5) | 0.0 (0.1) | 0.0 (0.1) | 0.0 (0.2) | 0.0 (0.9) | 0.0 (0.2) | 0.0 (0.4) | 0.0 (0.4) | 0.0 (0.4) |
| Southern Coastal Streams-Queen Charlotte Strait-Johnstone Strait-Southern Fjords | 0.3 (1.4) | 0.0 (0.1) | 0.3 (0.7) | 6.5 (4.6) | 0.1 (0.9) | 4.5 (3.1) | 0.3 (1.8) | 0.0 (1.2) | 4.4 (4.1) |
| Homathko-Klinaklini Rivers | 0.0 (0.3) | 0.4 (0.7) | 7.3 (3.2) | 3.9 (4.6) | 0.0 (0.7) | 0.0 (0.1) | 0.0 (0.1) | 0.0 (0.7) | 0.0 (1.0) |
| Georgia Strait Mainland | 0.0 (0.3) | 0.0 (0.1) | 0.0 (0.1) | 0.0 (0.2) | 0.0 (0.6) | 0.0 (0.1) | 0.0 (0.9) | 0.0 (0.5) | 0.0 (0.5) |
| Howe Sound-Burrard Inlet | 5.2 (4.6) | 19.4 (3.6) | 24.2 (5.2) | 9.2 (6.5) | 41.1 (12.6) | 21.1 (5.4) | 11.8 (7.8) | 25.3 (11.7) | 14.0 (6.8) |
| East Vancouver Island-Georgia Strait | 26.4 (9.6) | 22.3 (3.7) | 19.3 (4.5) | 32.9 (8.3) | 6.2 (6.2) | 11.1 (4.6) | 5.9 (5.5) | 30.8 (11.8) | 26.4 (9.7) |
| East Vancouver Island-Johnstone Strait-Southern Fjords | 1.3 (2.9) | 0.0 (0.1) | 0.0 (0.2) | 0.1 (1.4) | 0.1 (1.1) | 0.1 (0.5) | 7.4 (6.2) | 0.0 (0.6) | 0.0 (0.4) |
| Nahwitti Lowland | 0.0 (1.0) | 0.0 (0.1) | 0.0 (0.2) | 0.0 (0.4) | 0.0 (1.0) | 0.0 (0.3) | 0.0 (0.9) | 0.0 (0.9) | 16.7 (7.7) |
| West Vancouver Island | 0.0 (0.5) | 0.0 (0.1) | 0.0 (0.2) | 0.0 (0.2) | 0.0 (0.6) | 0.1 (0.3) | 0.0 (0.6) | 0.0 (0.8) | 0.0 (0.4) |
| Clayoquot | 0.1 (0.9) | 0.0 (0.1) | 0.0 (0.2) | 0.0 (0.2) | 0.0 (0.3) | 0.1 (0.6) | 0.0 (0.4) | 0.0 (1.0) | 0.0 (0.7) |
| Juan de Fuca-Pachena | 0.0 (0.2) | 0.0 (0.1) | 0.0 (0.1) | 0.0 (0.4) | 0.0 (0.8) | 0.0 (0.2) | 0.0 (0.3) | 0.0 (0.3) | 0.0 (0.4) |
| Lower Fraser | 27.1 (9.7) | 37.4 (4.2) | 22.2 (4.6) | 22.9 (7.1) | 43.5 (12.6) | 22.7 (5.7) | 23.5 (9.6) | 29.8 (11.6) | 8.0 (6.0) |
| Lillooet | 4.8 (4.1) | 0.8 (0.7) | 0.0 (0.2) | 0.0 (0.4) | 0.0 (0.8) | 6.3 (3.1) | 0.0 (1.1) | 0.0 (0.7) | 0.0 (0.6) |
| Fraser Canyon | 0.1 (0.6) | 0.0 (0.1) | 0.1 (0.4) | 0.0 (0.2) | 0.0 (0.6) | 0.0 (0.1) | 0.0 (0.4) | 0.0 (0.2) | 0.0 (0.2) |
| Interior Fraser | 0.0 (0.5) | 0.0 (0.1) | 0.0 (0.1) | 0.0 (0.3) | 0.0 (0.4) | 3.3 (2.3) | 5.8 (5.1) | 0.0 (0.6) | 0.0 (0.8) |
| Lower Thompson | 4.7 (4.7) | 0.0 (0.0) | 0.0 (0.1) | 0.0 (0.4) | 0.0 (0.3) | 1.4 (1.6) | 0.0 (0.4) | 0.0 (0.5) | 4.3 (4.1) |
| North Thompson | 0.0 (1.2) | 0.0 (0.2) | 0.0 (0.3) | 2.9 (2.7) | 0.0 (1.3) | 0.0 (0.4) | 0.0 (1.2) | 0.0 (1.7) | 4.3 (4.1) |
| South Thompson | 4.8 (4.3) | 0.0 (0.2) | 0.0 (0.3) | 0.0 (0.7) | 0.0 (1.4) | 0.0 (0.4) | 0.0 (1.0) | 0.0 (1.0) | 0.0 (1.0) |
| Boundary Bay | 1.5 (3.4) | 3.9 (1.8) | 2.0 (1.5) | 2.8 (2.9) | 7.6 (6.7) | 8.0 (3.4) | 5.8 (5.1) | 0.0 (0.4) | 0.0 (0.8) |
| Nooksack River | 15.5 (9.3) | 10.6 (3.0) | 15.8 (4.2) | 13.1 (5.6) | 0.5 (2.5) | 15.0 (4.9) | 27.5 (11.1) | 4.8 (1.0) | 5.2 (8.5) |
| Skagit River | 0.6 (3.6) | 1.4 (1.5) | 3.8 (2.5) | 0.1 (1.9) | 0.3 (0.4) | 5.6 (3.3) | 0.0 (0.6) | 0.5 (0.7) | 0.0 (0.4) |
| Northern Puget Sound | 7.2 (6.4) | 2.2 (1.5) | 0.0 (0.3) | 0.1 (1.2) | 0.5 (1.3) | 0.3 (0.8) | 0.1 (0.9) | 7.7 (9.1) | 0.0 (0.5) |
| Mid-Puget Sound | 0.5 (1.4) | 0.0 (0.2) | 0.1 (0.2) | 4.6 (3.7) | 0.0 (0.5) | 0.0 (0.2) | 5.9 (5.1) | 0.0 (0.3) | 3.6 (3.7) |
| Southern Puget Sound | 0.0 (0.5) | 0.0 (0.1) | 2.9 (1.9) | 0.0 (0.4) | 0.0 (0.6) | 0.0 (0.3) | 0.0 (0.6) | 0.4 (2.9) | 0.0 (0.3) |
| Juan de Fuca Strait | 0.0 (0.3) | 0.0 (0.1) | 0.2 (0.7) | 0.0 (0.2) | 0.0 (0.5) | 0.0 (0.1) | 0.0 (0.6) | 0.1 (0.5) | 0.0 (0.5) |
| Hood Canal | 0.0 (0.4) | 0.8 (0.8) | 0.0 (0.1) | 0.0 (0.3) | 0.0 (0.6) | 0.0 (0.3) | 0.0 (1.0) | 0.0 (0.5) | 0.0 (0.3) |
| Coastal Washington | 0.0 (0.7) | 0.0 (0.3) | 0.8 (1.0) | 0.0 (0.3) | 0.0 (0.8) | 0.0 (0.3) | 0.0 (0.9) | 0.0 (0.9) | 6.3 (4.7) |
| Columbia River | 0.0 (0.7) | 0.8 (0.7) | 1.1 (1.1) | 0.0 (0.4) | 0.0 (1.0) | 0.0 (0.2) | 5.9 (5.7) | 0.0 (1.3) | 4.3 (4.2) |
| Oregon | 0.0 (1.0) | 0.0 (0.1) | 0.0 (0.3) | 0.0 (0.5) | 0.0 (1.4) | 0.0 (0.3) | 0.0 (1.1) | 0.0 (1.0) | 2.3 (4.4) |

Supplementary Table 2 continued

| Region/Conservation Unit | SOG/JDF | | WCVI/JDF | | SWVI 21/121 | | SWVI Inshore | | |
| --- | --- | --- | --- | --- | --- | --- | --- | --- | --- |
|  | August | Sept | August | Sept | June | July | July | August | Sept |
| Sample size | 12 | 42 | 9 | 36 | 16 | 15 | 16 | 39 | 26 |
| Southeast Alaska | 0.0 (1.4) | 0.0 (0.6) | 0.0 (1.7) | 0.0 (0.6) | 0.0 (1.6) | 0.0 (1.2) | 0.0 (1.0) | 0.1 (0.6) | 0.0 (0.5) |
| Alsek River | 0.0 (0.7) | 0.0 (0.2) | 0.0 (1.1) | 0.0 (0.1) | 0.0 (0.6) | 0.0 (0.4) | 0.0 (0.3) | 0.0 (0.2) | 0.0 (0.1) |
| Lower Stikine | 0.0 (0.5) | 0.0 (0.1) | 0.0 (0.2) | 0.0 (0.2) | 0.0 (0.3) | 0.0 (0.6) | 0.0 (0.8) | 0.0 (0.1) | 0.0 (0.0) |
| Lower Nass | 0.0 (0.5) | 0.0 (0.4) | 0.1 (0.8) | 0.0 (0.1) | 0.0 (0.6) | 0.0 (0.8) | 0.0 (0.4) | 0.0 (0.1) | 0.0 (0.2) |
| Upper Nass | 0.0 (0.6) | 0.0 (0.2) | 0.0 (0.7) | 0.0 (0.0) | 0.0 (0.1) | 0.0 (0.1) | 0.0 (0.4) | 0.0 (0.2) | 0.0 (0.1) |
| Portland Sound-Observatory Inlet-Portland Canal | 0.0 (0.1) | 0.0 (0.2) | 0.0 (1.0) | 0.0 (0.1) | 0.0 (0.4) | 0.0 (0.1) | 0.0 (0.3) | 0.0 (0.1) | 0.0 (0.1) |
| Skeena Estuary | 0.0 (1.1) | 0.0 (0.3) | 0.0 (0.9) | 0.0 (0.3) | 0.0 (0.8) | 0.0 (0.6) | 0.0 (0.6) | 0.0 (0.4) | 0.0 (0.5) |
| Lower Skeena | 0.0 (1.1) | 0.0 (0.3) | 0.0 (1.6) | 0.0 (0.3) | 0.0 (0.6) | 0.0 (0.9) | 0.0 (0.9) | 0.0 (0.4) | 0.0 (0.3) |
| Middle Skeena | 0.0 (0.7) | 0.0 (0.4) | 0.0 (1.0) | 0.0 (0.2) | 0.0 (0.7) | 0.0 (0.9) | 0.0 (1.0) | 0.0 (0.3) | 0.0 (0.3) |
| Upper Skeena | 0.0 (1.0) | 0.0 (0.1) | 0.0 (0.9) | 0.0 (0.4) | 0.0 (0.7) | 0.0 (0.3) | 0.0 (0.7) | 0.0 (0.3) | 0.0 (0.4) |
| Haida Gwaii-Graham Island Lowlands | 0.0 (1.3) | 0.0 (0.3) | 0.0 (1.9) | 0.0 (0.6) | 0.0 (1.3) | 0.0 (1.1) | 0.0 91.1) | 0.0 (0.4) | 0.0 90.6) |
| Haida Gwaii-East | 0.0 (1.4) | 0.0 (0.3) | 0.0 (1.2) | 0.0 (0.3) | 0.0 (0.6) | 0.0 (1.0) | 0.0 (0.5) | 0.0 (0.3) | 0.0 (0.4) |
| Haida Gwaii-West | 0.0 (1.0) | 0.0 (0.3) | 0.0 (1.5) | 0.0 (0.6) | 0.0 (0.5) | 0.0 (0.9) | 0.0 (0.6) | 0.0 (0.2) | 0.0 (0.3) |
| Northern Coastal Streams | 2.4 (7.2) | 0.0 (0.4) | 0.0 (2.1) | 0.0 (0.8) | 0.0 (1.5) | 0.0 (1.5) | 0.3 (1.9) | 0.2 (1.1) | 0.1 (0.8) |
| Hecate Strait Mainland | 0.0 (1.1) | 0.0 (0.4) | 0.0 (1.6) | 0.0 (0.3) | 0.0 (1.5) | 0.0 (0.7) | 0.4 (2.1) | 0.0 (0.3) | 0.9 (2.8) |
| Mussel-Kynoch | 0.0 (0.3) | 0.0 (0.0) | 0.0 (0.5) | 0.0 (0.1) | 0.0 (0.2) | 0.0 (0.6) | 0.0 (0.5) | 0.0 (0.1) | 0.0 (0.1) |
| Douglas Channel-Kitimat Arm | 0.0 (0.9) | 0.0 (0.2) | 0.0 (1.3) | 0.0 (0.3) | 0.0 (0.6) | 0.0 (0.7) | 0.0 (0.9) | 0.0 (0.5) | 0.0 (0.4) |
| Bella Coola-Dean Rivers | 0.0 (1.4) | 0.0 (0.6) | 4.1 (8.5) | 0.0 (0.4) | 0.0 (1.1) | 0.0 (0.9) | 0.9 (2.2) | 0.0 (0.6) | 0.0 (0.4) |
| Rivers Inlet | 0.0 (0.7) | 0.0 (0.3) | 0.1 (1.9) | 0.0 (0.3) | 0.0 (0.7) | 0.0 (0.8) | 4.2 (5.7) | 0.0 (0.3) | 0.5 (1.2) |
| Smith Inlet | 0.0 (0.6) | 0.0 (0.3) | 2.9 (2.3) | 0.0 (0.3) | 0.0 (0.9) | 0.0 (0.6) | 0.0 (0.3) | 0.0 (0.3) | 0.0 (0.2) |
| Southern Coastal Streams-Queen Charlotte Strait-Johnstone Strait-Southern Fjords | 0.0 (1.1) | 0.2 (0.6) | 15.7 (12.0) | 0.0 (0.4) | 1.3 (4.6) | 0.0 (1.1) | 1.0 (2.0) | 0.0 (0.4) | 1.4 (2.4) |
| Homathko-Klinaklini Rivers | 0.0 (0.1) | 0.0 (0.2) | 0.0 (1.3) | 0.0 (0.0) | 0.0 (0.3) | 0.0 (0.3) | 0.0 90.2) | 0.0 (0.3) | 0.0 (0.1) |
| Georgia Strait Mainland | 0.0 (0.5) | 0.0 (0.1) | 0.0 (0.8) | 0.0 (0.3) | 0.0 (0.5) | 0.0 (0.6) | 0.0 (0.4) | 0.0 (0.1) | 0.0 (0.3) |
| Howe Sound-Burrard Inlet | 0.0 (1.2) | 8.5 (4.7) | 10.3 (9.4) | 5.9 (4.0) | 0.0 (0.8) | 13.4 (7.9) | 3.8 (4.2) | 1.3 (2.2) | 0.0 (0.6) |
| East Vancouver Island-Georgia Strait | 14.8 (11.0) | 24.3 (6.6) | 22.7 (12.2) | 27.1 (8.4) | 0.0 (1.5) | 0.0 (1.2) | 6.2 (6.5) | 14.5 (6.3) | 23.3 (8.9) |
| East Vancouver Island-Johnstone Strait-Southern Fjords | 0.1 (0.7) | 0.0 (0.2) | 0.0 (0.80 | 0.0 (0.4) | 0.0 (0.8) | 0.0 (0.7) | 0.0 (1.2) | 0.0 (0.2) | 0.0 (0.5) |
| Nahwitti Lowland | 0.0 (1.3) | 0.0 (0.4) | 0.0 (2.3) | 0.2 (1.6) | 0.0 (1.5) | 0.0 (0.9) | 0.1 (1.4) | 0.0 (0.5) | 0.1 (0.7) |
| West Vancouver Island | 0.0 (0.8) | 0.0 (0.3) | 0.0 (1.2) | 1.6 (2.8) | 0.0 (0.9) | 0.0 (1.0) | 8.3 (6.6) | 80.2 (7.1) | 53.3 (9.8) |
| Clayoquot | 0.0 (0.8) | 0.0 (0.3) | 0.0 (1.5) | 1.4 (1.9) | 0.0 (0.7) | 0.0 (0.6) | 28.5 (11.0) | 0.9 (2.7) | 4.5 (4.0) |
| Juan de Fuca-Pachena | 0.0 (0.7) | 0.0 (0.2) | 0.0 (0.7) | 9.6 (4.9) | 0.0 (0.5) | 0.0 (0.5) | 12.9 (7.8) | 0.1 (1.3) | 0.0 (0.4) |
| Lower Fraser | 0.0 (2.2) | 14.5 (5.4) | 11.1 (9.4) | 25.2 (6.8) | 20.5 (10.0) | 13.5 (8.0) | 2.2 (5.4) | 0.0 (0.8) | 0.0 (1.2) |
| Lillooet | 8.3 (7.3) | 4.8 (3.2) | 0.0 (1.2) | 2.8 (2.7) | 0.0 (0.7) | 0.0 (1.3) | 0.0 (1.0) | 2.6 (2.5) | 0.0 (0.4) |
| Fraser Canyon | 0.0 (0.6) | 0.0 (0.1) | 0.0 (1.0) | 0.0 (0.1) | 0.0 (0.4) | 0.0 (0.7) | 0.0 (0.5) | 0.0 (0.1) | 0.0 (0.3) |
| Interior Fraser | 0.0 (1.0) | 1.0 (1.9) | 11.1 (8.7) | 0.0 (0.4) | 0.0 (0.8) | 0.0 (0.6) | 0.0 (0.8) | 0.0 (0.3) | 0.0 (0.5) |
| Lower Thompson | 8.3 (6.80 | 0.0 (0.2) | 0.0 (0.7) | 2.8 (2.6) | 0.0 (0.4) | 0.0 (0.7) | 0.0 (0.7) | 0.0 (0.1) | 0.0 (0.2) |
| North Thompson | 0.0 (2.1) | 0.0 (0.7) | 0.0 (2.2) | 0.0 (0.5) | 0.0 (1.4) | 0.0 (1.5) | 0.0 (1.4) | 0.0 (0.5) | 0.0 (1.0) |
| South Thompson | 0.0 (1.60 | 3.7 (3.1) | 0.0 (2.6) | 0.0 (0.6) | 0.0 (1.6) | 0.0 (1.4) | 0.0 (1.4) | 0.0 (0.6) | 0.0 (0.8) |
| Boundary Bay | 8.3 97.3) | 12.9 (5.3) | 0.0 (1.7) | 2.8 (2.5) | 1.0 (2.5) | 0.0 (0.3) | 0.0 (0.7) | 0.0 (0.3) | 0.0 (0.5) |
| Nooksack River | 18.0 (15.5) | 12.5 (5.5) | 0.0 (0.7) | 1.9 (4.3) | 0.0 (0.4) | 5.6 (12.4) | 0.1 (0.7) | 0.0 (0.2) | 7.8 (5.0) |
| Skagit River | 32.7 (15.4) | 1.4 (3.5) | 6.2 (8.3) | 0.7 (2.9) | 0.1 (1.0) | 0.1 (1.5) | 0.0 (0.7) | 0.0 (0.2) | 0.0 (0.7) |
| Northern Puget Sound | 6.8 (14.6) | 15.4 (7.4) | 0.1 (1.7) | 7.1 (6.3) | 30.4 (11.5) | 41.7 (14.5) | 0.2 (0.9) | 0.0 (0.3) | 0.0 (0.5) |
| Mid-Puget Sound | 0.1 (0.6) | 0.8 (2.2) | 0.5 (1.0) | 6.6 (6.7) | 0.0 (0.4) | 13.2 (10.1) | 1.1 (4.0) | 0.0 (0.2) | 0.0 (0.3) |
| Southern Puget Sound | 0.1 (0.7) | 0.0 (0.2) | 0.0 (1.3) | 0.4 (0.8) | 13.3 (8.0) | 5.9 (5.9) | 4.5 (8.0) | 0.0 (0.3) | 0.0 (0.5) |
| Juan de Fuca Strait | 0.0 (0.6) | 0.0 (0.2) | 15.1 (11.5) | 0.1 (0.1) | 0.0 (0.7) | 0.0 (0.4) | 0.1 (0.4) | 0.0 (0.1) | 0.2 (2.2) |
| Hood Canal | 0.0 (0.4) | 0.0 (0.2) | 0.0 (0.6) | 0.0 (0.3) | 20.0 (9.3) | 0.0 (0.3) | 12.2 (7.8) | 0.0 (0.1) | 0.0 (0.3) |
| Coastal Washington | 0.0 (0.9) | 0.0 (0.6) | 0.0 (2.0) | 3.4 (2.9) | 6.7 (6.0) | 0.0 (1.1) | 0.6 (2.8) | 0.0 (0.5) | 7.8 (5.6) |
| Columbia River | 0.0 (1.3) | 0.0 (0.4) | 0.0 (1.8) | 0.0 (0.6) | 6.7 (5.7) | 6.7 (6.1) | 12.5 (7.7) | 0.0 (0.4) | 0.0 (0.5) |
| Oregon | 0.0 (1.5) | 0.0 (0.5) | 0.0 (1.7) | 0.4 (2.3) | 0.0 (1.2) | 0.0 (1.2) | 0.0 (1.1) | 0.0 (0.5) | 0.0 (0.6) |

Supplementary Table 2 continued.

| Region/Conservation Unit | SWVI offshore | | NWVI inshore | | NWVI offshore | Area 27 troll test | Area 127 troll test | Area 126 troll test | Round Island test |
| --- | --- | --- | --- | --- | --- | --- | --- | --- | --- |
|  | July | August | July | August | July | July 13-30 | July 14-29 | July 14-23 | July 12-31 |
| Sample size | 19 | 12 | 247 | 189 | 12 | 146 | 100 | 23 | 32 |
| Southeast Alaska | 0.0 (0.8) | 0.0 (1.6) | 0.1 (0.4) | 0.0 (0.2) | 0.1 (1.9) | 0.0 (0.1) | 0.0 (0.1) | 0.0 (0.1) | 0.0 (0.8) |
| Alsek River | 0.0 (0.20 | 0.0 (0.3) | 0.0 (0.0) | 0.0 (0.0) | 0.0 (1.3) | 0.0 (0.1) | 0.0 (0.0) | 0.0 (0.0) | 0.0 (0.1) |
| Lower Stikine | 0.0 (0.2) | 0.0 (0.3) | 0.0 (0.0) | 0.0 (0.0) | 0.0 (0.8) | 0.0 (0.0) | 0.0 (0.0) | 0.0 (0.0) | 0.0 (0.1) |
| Lower Nass | 0.0 (0.4) | 0.0 (0.9) | 0.1 (0.3) | 0.0 (0.1) | 0.0 (0.6) | 0.0 (0.2) | 0.0 (0.0) | 0.0 (0.1) | 0.0 (0.1) |
| Upper Nass | 0.0 (0.3) | 0.0 (0.7) | 0.0 (0.0) | 0.0 (0.0) | 0.0 (0.6) | 0.0 (0.0) | 0.0 (0.1) | 0.0 (0.0) | 0.0 (0.1) |
| Portland Sound-Observatory Inlet-Portland Canal | 0.0 (0.2) | 0.0 (0.3) | 0.0 (0.0) | 0.0 (0.0) | 0.0 (0.3) | 0.0 (0.0) | 0.0 (0.0) | 0.0 (0.0) | 0.0 (0.1) |
| Skeena Estuary | 0.0 (0.5) | 0.0 (0.8) | 0.0 (0.0) | 0.0 (0.0) | 0.0 (0.8) | 0.0 (0.1) | 0.0 (0.0) | 0.0 (0.0) | 0.0 (0.2) |
| Lower Skeena | 0.0 (0.6) | 0.0 (0.9) | 0.2 (0.3) | 0.0 (0.1) | 0.0 (1.0) | 0.0 (0.2) | 0.0 (0.2) | 0.0 (0.1) | 0.0 (0.4) |
| Middle Skeena | 0.0 (0.5) | 0.0 (0.4) | 0.0 (0.0) | 0.0 (0.1) | 0.0 (0.7) | 0.0 (0.1) | 0.0 (0.0) | 0.0 (0.0) | 0.0 (0.4) |
| Upper Skeena | 0.0 (0.4) | 0.0 (0.9) | 0.0 (0.0) | 0.0 (0.1) | 0.0 (0.9) | 0.0 (0.0) | 0.0 (0.1) | 0.0 (0.0) | 0.0 (0.2) |
| Haida Gwaii-Graham Island Lowlands | 0.0 (0.7) | 0.0 (1.3) | 0.0 (0.1) | 0.0 (0.2) | 0.0 (1.8) | 0.0 (0.2) | 0.0 (0.1) | 0.0 (0.1) | 0.0 (0.5) |
| Haida Gwaii-East | 0.0 (0.8) | 0.0 (0.9) | 0.0 (0.0) | 0.0 (0.1) | 0.0 (1.5) | 0.0 (0.1) | 0.7 (0.7) | 0.0 (0.0) | 0.0 (0.3) |
| Haida Gwaii-West | 0.0 (0.5) | 0.0 (0.8) | 0.0 (0.0) | 0.0 (0.1) | 0.0 (1.0) | 0.0 (0.1) | 0.0 (0.1) | 0.0 (0.1) | 0.0 (0.3) |
| Northern Coastal Streams | 0.0 (1.1) | 0.0 (2.0) | 2.7 (2.0) | 8.0 (2.4) | 1.2 (7.1) | 3.2 (2.6) | 4.3 (2.5) | 0.1 (0.4) | 0.3 (1.1) |
| Hecate Strait Mainland | 0.0 (0.6) | 0.0 (1.2) | 1.8 91.0) | 2.3 (1.4) | 0.1 (1.3) | 0.4 (1.5) | 0.5 (1.2) | 4.3 (1.6) | 0.0 (0.3) |
| Mussel-Kynoch | 0.0 (0.1) | 0.0 (0.3) | 0.0 (0.0) | 0.0 (0.0) | 0.0 (0.5) | 0.0 (0.1) | 0.0 (0.0) | 0.0 (0.0) | 0.0 (0.2) |
| Douglas Channel-Kitimat Arm | 0.0 (0.5) | 0.0 (0.6) | 1.5 (1.4) | 0.2 (1.1) | 0.0 (1.1) | 0.1 (0.2) | 0.2 (1.2) | 1.1 (1.1) | 0.5 (2.6) |
| Bella Coola-Dean Rivers | 0.0 (0.9) | 0.1 (1.5) | 0.9 (1.0) | 0.0 (0.1) | 0.1 (2.4) | 0.9 (1.5) | 0.1 (0.3) | 0.2 (0.6) | 0.0 (0.5) |
| Rivers Inlet | 0.0 (0.5) | 0.0 (1.0) | 0.0 (0.1) | 1.7 (1.3) | 0.0 (0.9) | 0.2 (0.4) | 0.8 (0.9) | 0.1 (0.3) | 0.0 (0.6) |
| Smith Inlet | 0.0 (0.6) | 0.0 (1.0) | 0.0 (0.1) | 0.0 (0.0) | 0.3 (1.0) | 0.0 (0.1) | 0.0 (0.1) | 0.1 (0.2) | 0.4 (1.3) |
| Southern Coastal Streams-Queen Charlotte Strait-Johnstone Strait-Southern Fjords | 0.0 (0.4) | 0.1 (1.6) | 3.5 (1.5) | 3.4 (1.9) | 1.1 (2.8) | 2.9 (2.0) | 6.7 (2.4) | 1.7 (1.5) | 7.1 (5.3) |
| Homathko-Klinaklini Rivers | 0.0 (0.2) | 0.0 (0.6) | 1.6 (1.1) | 1.2 (1.0) | 0.0 (0.8) | 6.0 (2.5) | 0.6 (1.3) | 4.5 (1.7) | 0.7 (3.2) |
| Georgia Strait Mainland | 0.0 (0.5) | 0.0 (0.5) | 0.0 (0.0) | 0.0 (0.1) | 0.0 (0.4) | 0.0 (0.1) | 0.0 (0.2) | 0.0 (0.0) | 0.0 (0.2) |
| Howe Sound-Burrard Inlet | 5.3 (4.6) | 15.2 (10.0) | 1.6 (1.0) | 2.0 (1.5) | 0.1 (1.7) | 3.8 (2.2) | 4.6 (2.3) | 9.3 (2.1) | 10.9 (6.6) |
| East Vancouver Island-Georgia Strait | 2.9 (5.0) | 17.2 (10.4) | 12.5 (2.5) | 10.9 (3.2) | 12.6 (9.5) | 6.3 (3.5) | 11.5 (3.3) | 26.3 (3.0) | 44.6 (10.5) |
| East Vancouver Island-Johnstone Strait-Southern Fjords | 0.0 (0.2) | 0.0 (0.6) | 0.0 (0.2) | 0.0 (0.1) | 0.0 (0.8) | 0.1 (0.2) | 0.0 (0.1) | 0.0 (0.1) | 1.4 (4.6) |
| Nahwitti Lowland | 0.0 (1.0) | 0.0 (1.4) | 30.0 (3.3) | 34.8 (4.0) | 6.7 (8.9) | 22.4 (4.8) | 27.6 (4.0) | 3.4 (1.5) | 2.9 (3.1) |
| West Vancouver Island | 0.0 (0.5) | 16.7 (10.0) | 18.2 (2.8) | 11.5 (2.7) | 5.0 (8.6) | 14.5 (4.0) | 14.1 (3.5) | 7.6 (1.9) | 3.6 (4.9) |
| Clayoquot | 0.0 (1.0) | 0.0 (0.5) | 9.6 (2.1) | 9.6 (2.5) | 2.2 (5.6) | 10.9 (4.0) | 6.4 (2.6) | 3.4 (1.5) | 3.2 (3.1) |
| Juan de Fuca-Pachena | 5.2 (4.7) | 0.0 (0.6) | 3.3 (1.3) | 2.3 (1.8) | 0.0 (0.8) | 2.2 (1.7) | 8.3 (2.8) | 2.2 (1.0) | 0.0 (0.1) |
| Lower Fraser | 5.3 (5.2) | 0.1 (2.3) | 0.5 (0.5) | 1.9 (1.1) | 0.0 (3.1) | 2.0 (1.4) | 0.0 (0.3) | 5.1 (1.5) | 0.0 (0.7) |
| Lillooet | 10.5 (6.6) | 0.0 (1.2) | 0.8 (0.6) | 0.0 (0.1) | 0.0 (1.8) | 1.0 (0.9) | 0.0 (0.1) | 0.4 (0.4) | 0.0 (0.3) |
| Fraser Canyon | 0.0 (0.2) | 0.0 (0.5) | 0.0 (0.0) | 0.0 (0.0) | 0.0 (0.9) | 0.0 (0.0) | 0.0 (0.1) | 0.0 (0.0) | 0.0 (0.4) |
| Interior Fraser | 0.0 (0.6) | 0.0 (1.0) | 0.0 (0.1) | 0.0 (0.1) | 0.0 (1.4) | 0.0 (0.2) | 0.0 (0.1) | 0.4 (0.4) | 0.0 (0.4) |
| Lower Thompson | 0.0 (0.8) | 0.0 (0.6) | 0.0 (0.0) | 0.0 (0.0) | 0.0 (0.8) | 1.0 (1.0) | 0.0 (0.1) | 0.8 (0.6) | 0.0 (0.2) |
| North Thompson | 0.0 (1.1) | 0.0 (2.1) | 0.0 (0.1) | 0.1 (0.4) | 0.0 (2.3) | 0.0 (0.2) | 0.0 (0.2) | 0.8 (0.6) | 0.0 (0.8) |
| South Thompson | 0.0 (1.1) | 0.0 (1.6) | 0.0 (0.1) | 0.5 (0.5) | 0.0 (2.1) | 0.0 (0.2) | 0.0 (0.2) | 1.3 (0.8) | 3.1 (3.2) |
| Boundary Bay | 0.0 (0.7) | 0.0 (0.8) | 0.0 (0.0) | 0.5 (0.5) | 0.0 (1.0) | 0.0 (0.0) | 0.0 (0.1) | 0.4 (0.4) | 0.0 (0.3) |
| Nooksack River | 0.0 (0.6) | 25.8 (13.1) | 2.0 (1.1) | 2.0 (1.3) | 13.2 (15.0) | 0.1 (0.4) | 0.0 (0.1) | 0.0 (0.2) | 0.0 (0.6) |
| Skagit River | 0.1 (0.6) | 0.0 (0.7) | 0.0 (0.1) | 0.0 (0.1) | 1.8 (6.5) | 0.1 (0.1) | 2.6 (1.5) | 0.0 (0.2) | 1.4 (2.9) |
| Northern Puget Sound | 17.0 (9.5) | 0.2 (1.5) | 3.4 (1.4) | 0.8 (0.6) | 7.5 (9.9) | 6.3 (3.3) | 2.1 (1.6) | 5.7 (1.8) | 4.2 (4.9) |
| Mid-Puget Sound | 6.6 (8.5) | 7.5 (7.0) | 0.0 (0.0) | 1.6 (0.9) | 0.2 (4.6) | 5.6 (2.9) | 4.1 (2.5) | 8.4 (2.1) | 0.0 (0.5) |
| Southern Puget Sound | 8.0 (8.9) | 0.3 (2.2) | 0.0 (0.1) | 0.4 (1.0) | 0.1 (0.8) | 0.1 (0.5) | 0.0 (0.4) | 0.9 (0.8) | 0.0 (1.0) |
| Juan de Fuca Strait | 0.0 (0.3) | 0.2 (0.8) | 0.0 (0.2) | 0.0 (0.3) | 0.0 (1.8) | 0.6 (1.2) | 0.3 (1.2) | 0.3 (0.9) | 0.0 (0.3) |
| Hood Canal | 5.3 (4.8) | 0.0 (0.6) | 1.6 (0.8) | 0.5 (0.5) | 0.1 (2.6) | 0.0 (0.1) | 1.7 (1.3) | 0.7 (0.7) | 0.0 (0.3) |
| Coastal Washington | 28.5 (10.3) | 8.4 (7.0) | 2.7 (1.2) | 3.5 (1.4) | 17.5 (12.7) | 5.2 (2.2) | 1.5 (1.0) | 8.7 (1.9) | 15.4 (6.5) |
| Columbia River | 5.3 (4.5) | 8.3 (7.4) | 1.2 (0.7) | 0.5 (0.5) | 30.0 (12.7) | 4.0 (2.0) | 1.4 (1.0) | 1.7 (0.8) | 0.0 (0.4) |
| Oregon | 0.0 (1.1) | 0.0 (1.7) | 0.0 (0.1) | 0.0 (0.1) | 0.0 (2.5) | 0.0 (0.2) | 0.0 (0.3) | 0.0 (0.1) | 0.0 (0.6) |

Supplementary Table 2 concluded.

| Region/Conservation Unit | Round Island test | Area 7-7 | Area 7-7 | Area 6-9 |
| --- | --- | --- | --- | --- |
|  | Aug 1-9 | Aug 2 | Aug 16 | Sept 8 |
| Sample size | 207 | 36 | 43 | 36 |
| Southeast Alaska | 0.0 (0.1) | 0.1 (0.6) | 0.0 (0.8) | 0.0 (0.5) |
| Alsek River | 0.0 (0.0) | 0.0 (0.1) | 0.0 (0.2) | 0.0 (0.1) |
| Lower Stikine | 0.0 (0.0) | 0.0 (0.3) | 0.0 (0.1) | 0.0 (0.1) |
| Lower Nass | 0.0 (0.0) | 0.0 (0.1) | 0.0 (0.2) | 0.0 (0.2) |
| Upper Nass | 0.0 (0.0) | 0.0 (0.1) | 0.0 (0.1) | 0.0 (0.1) |
| Portland Sound-Observatory Inlet-Portland Canal | 0.0 (0.0) | 0.0 (0.1) | 0.0 (0.2) | 0.0 (0.2) |
| Skeena Estuary | 0.0 (0.0) | 0.0 (0.3) | 0.0 (0.3) | 0.0 (0.2) |
| Lower Skeena | 0.0 (0.0) | 0.1 (0.6) | 0.1 (1.5) | 2.4 (2.6) |
| Middle Skeena | 0.0 (0.1) | 0.0 (0.2) | 0.0 (0.2) | 0.0 (0.2) |
| Upper Skeena | 0.0 (0.0) | 0.0 (0.2) | 0.0 (0.2) | 0.0 (0.2) |
| Haida Gwaii-Graham Island Lowlands | 0.0 (0.1) | 0.0 (0.3) | 0.0 (0.4) | 0.0 (0.4) |
| Haida Gwaii-East | 0.0 (0.0) | 0.0 (0.3) | 0.0 (0.4) | 0.0 (0.3) |
| Haida Gwaii-West | 0.0 (0.1) | 0.0 (0.2) | 0.0 (0.3) | 0.0 (0.2) |
| Northern Coastal Streams | 0.1 (0.4) | 17.1 (6.9) | 0.0 (0.7) | 0.4 (1.3) |
| Hecate Strait Mainland | 4.7 (1.6) | 35.2 (7.8) | 27.2 (7.8) | 14.0 (5.5) |
| Mussel-Kynoch | 0.0 (0.0) | 0.0 (0.1) | 0.0 (0.2) | 4.7 (2.9) |
| Douglas Channel-Kitimat Arm | 0.9 (1.0) | 29.3 (7.2) | 23.2 (7.4) | 0.0 (0.4) |
| Bella Coola-Dean Rivers | 0.3 (0.6) | 0.2 (0.3) | 0.0 (0.5) | 12.3 (5.0) |
| Rivers Inlet | 0.2 (0.4) | 6.5 (4.8) | 0.0 (0.2) | 4.3 (3.3) |
| Smith Inlet | 0.0 (0.1) | 0.0 (0.3) | 0.0 (0.5) | 0.0 (0.3) |
| Southern Coastal Streams-Queen Charlotte Strait-Johnstone Strait-Southern Fjords | 2.1 (1.3) | 5.5 (4.1) | 15.2 (6.0) | 18.7 (6.6) |
| Homathko-Klinaklini Rivers | 4.5 (1.8) | 0.0 (0.5) | 0.0 (0.0) | 0.1 (0.8) |
| Georgia Strait Mainland | 0.0 (0.0) | 0.0 (0.2) | 0.0 (0.5) | 0.0 (0.2) |
| Howe Sound-Burrard Inlet | 9.0 (2.4) | 0.0 (0.4) | 0.0 (0.5) | 13.2 (5.7) |
| East Vancouver Island-Georgia Strait | 23.7 (3.3) | 0.0 (0.4) | 0.0 (0.7) | 8.6 (4.5) |
| East Vancouver Island-Johnstone Strait-Southern Fjords | 0.0 (0.1) | 0.0 (0.2) | 0.0 (0.1) | 0.0 (0.4) |
| Nahwitti Lowland | 4.2 (1.6) | 1.5 (2.6) | 0.0 (0.4) | 18.3 (6.1) |
| West Vancouver Island | 7.9 (2.0) | 0.1 (0.9) | 0.0 (0.3) | 2.3 (2.3) |
| Clayoquot | 3.3 (1.4) | 0.1 (0.5) | 0.0 (0.2) | 0.4 (1.2) |
| Juan de Fuca-Pachena | 2.5 (1.2) | 0.0 (0.2) | 0.0 (0.2) | 0.0 (0.4) |
| Lower Fraser | 6.0 (1.8) | 2.2 (2.3) | 34.1 (7.7) | 0.1 (0.9) |
| Lillooet | 0.5 (0.5) | 0.0 (0.3) | 0.0 (0.4) | 0.0 (0.4) |
| Fraser Canyon | 0.0 (0.1) | 0.0 (0.2) | 0.0 (0.0) | 0.0 (0.1) |
| Interior Fraser | 0.5 (0.5) | 0.0 (0.2) | 0.0 (0.3) | 0.0 (0.3) |
| Lower Thompson | 1.0 (0.7) | 0.0 (0.2) | 0.0 (0.3) | 0.0 (0.1) |
| North Thompson | 1.0 (0.7) | 0.0 (0.5) | 0.0 (0.8) | 0.0 (0.6) |
| South Thompson | 1.0 (0.7) | 0.0 (0.5) | 0.0 (0.5) | 0.0 (0.6) |
| Boundary Bay | 0.5 (0.5) | 0.0 (0.2) | 0.0 (0.2) | 0.0 (0.3) |
| Nooksack River | 0.1 (0.3) | 0.0 (0.2) | 0.0 (0.1) | 0.0 (0.3) |
| Skagit River | 0.0 (0.1) | 0.0 (0.2) | 0.0 (0.3) | 0.0 (0.3) |
| Northern Puget Sound | 5.0 (1.9) | 0.0 (0.3) | 0.0 (0.5) | 0.0 (0.2) |
| Mid-Puget Sound | 9.1 (2.3) | 0.0 (0.2) | 0.0 (0.3) | 0.0 (0.2) |
| Southern Puget Sound | 1.2 (1.3) | 0.0 (0.2) | 0.0 (0.3) | 0.2 (0.7) |
| Juan de Fuca Strait | 0.5 (0.2) | 0.0 (0.2) | 0.0 (0.2) | 0.0 (0.2) |
| Hood Canal | 0.8 (0.9) | 0.0 (0.2) | 0.0 (0.2) | 0.0 (0.2) |
| Coastal Washington | 7.6 (1.9) | 0.0 (0.4) | 0.0 (0.4) | 0.0 (0.4) |
| Columbia River | 1.9 (0.9) | 2.2 (2.2) | 0.0 (0.5) | 0.0 (0.3) |
| Oregon | 0.0 (0.1) | 0.0 (0.4) | 0.0 (0.5) | 0.0 (0.4) |

Supplementary Table 3. Percentage stock composition by geographic region or CU of 2017 northern Area F ice boat troll fishery (only unmarked coho salmon sampled), Langara recreational fishery, central coast recreational fishery (primarily Area 8), central coast commercial freezer troll fishery (Areas 6, 7, 8, and 107), and Johnstone Strait (Round Island) test fishery. Standard deviation is in parentheses. N-PBT is the number of individuals identified in the sample via PBT.

| Region/Conservation Unit | 2017 Northern ice boat troll (Area F) unmarked catch | | | | | | | | Langara sport |
| --- | --- | --- | --- | --- | --- | --- | --- | --- | --- |
|  | July 10-12 | July 13-18 | July 22-28 | Jul 25-Aug 4 | Aug 11-15 | Aug 25-30 | Sept 1-5 | Sept 8-12 | Sept 2-8 |
| Sample size | 98 | 94 | 90 | 69 | 86 | 96 | 98 | 99 | 33 |
| N-PBT | 0 | 0 | 0 | 0 | 0 | 0 | 0 | 0 | 1 |
| Southeast Alaska | 0.0 (0.2) | 12.2 (3.8) | 3.1 (2.4) | 28.8 (8.6) | 13.5 (4.3) | 5.2 (3.1) | 8.9 (3.6) | 24.6 (5.0) | 3.5 (4.0) |
| Alsek River | 0.0 (0.1) | 0.0 (0.0) | 0.0 (0.1) | 0.0 (0.2) | 0.0 (0.1) | 0.0 (0.0) | 0.0 (0.1) | 0.0 (0.0) | 0.0 (0.1) |
| Lower Stikine | 0.0 (0.0) | 0.0 (0.1) | 0.0 (0.1) | 5.1 (3.2) | 5.6 (2.8) | 0.0 (0.1) | 0.0 (0.4) | 0.0 (0.2) | 0.0 (0.1) |
| Lower Nass | 0.0 (0.2) | 0.8 (1.5) | 1.8 (1.6) | 5.4 (2.8) | 0.1 (0.3) | 9.9 (3.3) | 10.4 (3.4) | 8.5 (3.5) | 0.0 (0.5) |
| Upper Nass | 0.4 (0.7) | 0.0 (0.3) | 0.1 (0.7) | 7.1 (4.1) | 3.8 (2.4) | 0.0 (0.1) | 0.0 (0.3) | 0.0 (0.1) | 0.0 (0.2) |
| Portland Sound-Observatory Inlet-Portland Canal | 0.0 (0.1) | 1.1 (1.1) | 0.0 (0.0) | 1.5 (1.4) | 0.0 (0.0) | 0.0 (0.0) | 0.0 (0.0) | 0.0 (0.1) | 0.0 (0.1) |
| Skeena Estuary | 1.0 (1.0) | 0.0 (0.0) | 0.0 (0.2) | 0.0 (0.3) | 0.0 (0.2) | 0.0 (0.2) | 1.0 (1.4) | 0.0 (0.1) | 0.0 (0.2) |
| Lower Skeena | 1.2 (1.3) | 0.7 (1.3) | 0.1 (0.5) | 1.9 (2.5) | 1.6 (1.4) | 4.3 (2.4) | 0.1 (0.4) | 2.8 (2.0) | 9.5 (5.1) |
| Middle Skeena | 0.5 (0.8) | 2.2 (1.5) | 1.1 (1.2) | 1.4 (1.6) | 0.7 (1.0) | 1.7 (1.9) | 0.9 (1.2) | 0.0 (0.2) | 0.0 (0.4) |
| Upper Skeena | 1.1 (1.4) | 9.2 (2.9) | 6.5 (2.6) | 0.1 (0.4) | 6.5 (2.8) | 1.2 (1.9) | 2.2 (1.5) | 0.1 (0.5) | 0.0 (0.4) |
| Haida Gwaii-Graham Island Lowlands | 1.6 (1.3) | 8.5 (2.8) | 32.5 (5.1) | 8.9 (4.7) | 23.0 (4.4) | 20.4 (4.0) | 12.3 (3.3) | 10.2 (3.1) | 42.4 (8.3) |
| Haida Gwaii-East | 0.0 (0.1) | 0.0 (0.2) | 1.1 (1.1) | 3.1 (2.4) | 1.2 (1.1) | 4.3 (2.0) | 1.1 (1.0) | 6.6 (2.5) | 6.1 (3.9) |
| Haida Gwaii-West | 0.0 (0.1) | 0.0 (0.1) | 1.1 (1.2) | 0.7 (1.4) | 0.0 (0.1) | 2.9 (1.8) | 0.0 (0.1) | 1.0 (1.0) | 0.0 (0.2) |
| Northern Coastal Streams | 0.4 (0.8) | 15.5 (4.1) | 9.8 (4.2) | 4.4 (4.6) | 11.2 (3.7) | 4.1 (4.5) | 21.1 (4.8) | 0.5 (1.1) | 8.1 (2.3) |
| Hecate Strait Mainland | 4.4 (2.2) | 3.4 (2.2) | 16.7 (4.3) | 5.5 (5.9) | 13.6 (4.6) | 15.9 (4.6) | 8.0 (3.2) | 13.3 (4.0) | 15.4 (8.1) |
| Mussel-Kynoch | 0.0 (0.1) | 0.0 (0.0) | 1.1 (1.1) | 0.0 (0.1) | 0.0 (0.1) | 0.0 (0.0) | 0.0 (0.1) | 0.0 (0.1) | 0.0 (0.2) |
| Douglas Channel-Kitimat Arm | 1.1 (2.3) | 15.1 (4.3) | 12.8 (3.9) | 22.9 (6.1) | 12.0 (4.2) | 15.7 (4.5) | 13.2 (4.1) | 18.6 (4.6) | 7.5 (7.9) |
| Bella Coola-Dean Rivers | 11.0 (3.9) | 12.0 (3.8) | 8.2 (3.2) | 0.1 (0.8) | 4.1 (3.1) | 3.8 (2.4) | 7.3 (3.3) | 0.1 (0.4) | 0.0 (0.7) |
| Rivers Inlet | 1.0 (1.4) | 3.5 (1.9) | 2.7 (2.2) | 1.4 (1.5) | 0.0 (0.2) | 4.2 (2.0) | 0.6 (1.0) | 0.3 (0.5) | 1.3 (2.7) |
| Smith Inlet | 0.0 (0.1) | 0.0 (0.1) | 0.0 (0.2) | 0.0 (0.4) | 0.1 (0.5) | 0.0 (0.1) | 0.0 (0.2) | 0.0 (0.1) | 0.2 (1.2) |
| Southern Coastal Streams-Queen Charlotte Strait-Johnstone Strait-Southern Fjords | 9.7 (3.3) | 5.8 (2.8) | 0.8 (1.3) | 0.1 (0.8) | 2.7 (2.1) | 6.0 (2.9) | 0.0 (0.3) | 7.0 (3.1) | 0.0 (0.4) |
| Homathko-Klinaklini Rivers | 0.8 (0.7) | 3.1 (2.2) | 0.0 (0.1) | 0.0 (0.1) | 0.3 (0.4) | 0.1 (0.6) | 0.4 (1.1) | 0.0 (0.0) | 0.0 (0.5) |
| Georgia Strait Mainland | 0.0 (0.1) | 0.0 (0.1) | 0.0 (0.1) | 0.0 (0.2) | 0.0 (0.0) | 0.0 (0.1) | 0.0 (0.1) | 0.0 (0.1) | 0.0 (0.2) |
| Howe Sound-Burrard Inlet | 11.5 (4.0) | 0.1 (1.2) | 0.0 (0.2) | 0.0 (0.5) | 0.0 (0.2) | 0.1 (0.4) | 0.0 (0.2) | 0.0 (0.4) | 0.4 (1.1) |
| East Vancouver Island-Georgia Strait | 15.2 (4.3) | 2.7 (1.9) | 0.0 (0.3) | 0.0 (0.7) | 0.0 (0.3) | 0.1 (0.7) | 5.3 (2.9) | 0.5 (0.9) | 0.3 (1.4) |
| East Vancouver Island-Johnstone Strait-Southern Fjords | 0.3 (1.4) | 0.0 (0.1) | 0.0 (0.1) | 0.0 (0.2) | 0.0 (0.1) | 0.0 (0.1) | 0.0 (0.1) | 0.7 (1.0) | 0.0 (0.3) |
| Nahwitti Lowland | 1.8 (1.4) | 0.1 (0.8) | 0.3 (0.8) | 0.1 (0.7) | 0.0 (0.4) | 0.0 (0.3) | 0.0 (0.2) | 0.0 (0.2) | 0.0 (0.5) |
| West Vancouver Island | 6.3 (2.6) | 0.4 (1.6) | 0.0 (0.2) | 1.4 (1.5) | 0.0 (0.2) | 0.0 (0.1) | 0.3 (0.7) | 0.0 (0.2) | 3.0 (2.9) |
| Clayoquot | 0.8 (1.4) | 2.4 (1.8) | 0.0 (0.1) | 0.0 (0.3) | 0.0 (0.4) | 0.0 (0.1) | 0.0 (0.2) | 0.0 (0.2) | 0.0 (0.3) |
| Juan de Fuca-Pachena | 0.0 (0.2) | 0.1 (0.2) | 0.0 (0.1) | 0.0 (0.2) | 0.0 (0.1) | 0.0 (0.2) | 0.0 (0.1) | 0.0 (0.1) | 0.0 (0.2) |
| Lower Fraser | 5.6 (2.5) | 1.1 (1.2) | 0.0 (0.3) | 0.0 (0.9) | 0.0 (0.3) | 0.0 (0.3) | 0.0 (0.3) | 0.0 (0.3) | 2.3 (3.0) |
| Lillooet | 0.0 (0.1) | 0.0 (0.1) | 0.0 (0.2) | 0.0 (0.5) | 0.0 (0.1) | 0.0 (0.2) | 0.0 (0.2) | 0.0 (0.1) | 0.0 (0.3) |
| Fraser Canyon | 0.0 (0.1) | 0.0 (0.1) | 0.0 (0.2) | 0.0 (0.3) | 0.0 (0.1) | 0.0 (0.1) | 0.0 (0.1) | 0.0 (0.0) | 0.0 (0.2) |
| Interior Fraser | 0.0 (0.1) | 0.0 (0.1) | 0.0 (0.2) | 0.0 (0.3) | 0.0 (0.1) | 0.0 (0.1) | 0.0 (0.1) | 0.0 (0.1) | 0.0 (0.4) |
| Lower Thompson | 0.0 (0.1) | 0.0 (0.1) | 0.0 (0.1) | 0.0 (0.3) | 0.0 (0.1) | 0.0 (0.1) | 0.0 (0.1) | 0.0 (0.1) | 0.0 (0.3) |
| North Thompson | 0.0 (0.2) | 0.0 (0.3) | 0.0 (0.2) | 0.0 (0.7) | 0.0 (0.2) | 0.0 (0.3) | 0.0 (0.2) | 0.0 (0.2) | 0.0 (0.6) |
| South Thompson | 1.0 (1.1) | 0.0 (0.3) | 0.0 (0.3) | 0.0 (0.5) | 0.0 (0.2) | 0.0 (0.3) | 0.0 (0.2) | 1.0 (1.0) | 0.0 (0.5) |
| Boundary Bay | 0.0 (0.1) | 0.0 (0.1) | 0.0 (0.1) | 0.0 (0.3) | 0.0 (0.1) | 0.0 (0.1) | 0.0 (0.1) | 0.0 (0.1) | 0.0 (0.3) |
| Nooksack River | 1.5 (2.9) | 0.0 (0.1) | 0.0 (0.1) | 0.0 (0.2) | 0.0 (0.1) | 0.0 (0.1) | 0.0 (0.1) | 0.0 (0.1) | 0.0 (0.2) |
| Skagit River | 0.9 (1.5) | 0.0 (0.1) | 0.0 (0.1) | 0.0 (0.3) | 0.0 (0.1) | 0.0 (0.1) | 0.0 (0.1) | 0.0 (0.1) | 0.0 (0.4) |
| Northern Puget Sound | 9.2 (3.9) | 0.2 (0.9) | 0.0 (0.1) | 0.0 (0.3) | 0.0 (0.2) | 0.0 (0.3) | 0.0 (0.1) | 0.0 (0.1) | 0.0 (0.4) |
| Mid-Puget Sound | 1.4 (2.5) | 0.0 (0.2) | 0.0 (0.1) | 0.0 (0.3) | 0.0 (0.1) | 0.0 (0.2) | 0.0 (0.2) | 0.0 (0.0) | 0.0 (0.3) |
| Southern Puget Sound | 0.1 (0.8) | 0.0 (0.1) | 0.0 (0.1) | 0.0 (0.3) | 0.0 (0.2) | 0.0 (0.1) | 0.0 (0.2) | 0.0 (0.1) | 0.0 (0.5) |
| Juan de Fuca Strait | 0.0 (0.1) | 0.0 (0.1) | 0.0 (0.1) | 0.0 (0.2) | 0.0 (0.2) | 0.0 (0.1) | 0.0 (0.1) | 0.0 (0.0) | 0.0 (0.1) |
| Hood Canal | 2.9 (1.7) | 0.0 (0.1) | 0.0 (0.1) | 0.0 (0.3) | 0.0 (0.1) | 0.0 (0.1) | 0.0 (0.1) | 0.0 (0.2) | 0.0 (0.2) |
| Coastal Washington | 5.1 (2.2) | 0.0 (0.2) | 0.0 (0.1) | 0.0 (0.7) | 0.0 (0.2) | 0.0 (0.1) | 6.9 (2.5) | 4.0 (1.9) | 0.0 (0.4) |
| Columbia River | 2.0 (1.4) | 0.0 (0.1) | 0.0 (0.2) | 0.0 (0.6) | 0.0 (0.2) | 0.0 (0.2) | 0.0 (0.2) | 0.0 (0.2) | 0.0 (0.5) |
| Oregon | 0.0 (0.2) | 0.0 (0.2) | 0.0 (0.2) | 0.0 (0.4) | 0.0 (0.3) | 0.0 (0.3) | 0.0 (0.2) | 0.0 (0.2) | 0.0 (0.4) |

Supplementary Table 3 continued.

| Region/Conservation Unit | Central coast sport | | Central coast commercial troll | | | | | | |
| --- | --- | --- | --- | --- | --- | --- | --- | --- | --- |
|  |  | | Area 6-9 | | | Area 6-11 | A 6-17, A7-31 | Area 7-1 | Area 107-3 |
|  | July 25-31 | Aug1-Sep1 | Aug 6-12 | Aug 20-26 | Sept 10-16 | Sept 10-16 | Aug 13-19 | Aug 13-19 | Oct 8-14 |
| Sample size | 436 | 106 | 108 | 77 | 46 | 47 | 32 | 30 | 87 |
| N-PBT | 1 | 0 | 0 | 0 | 0 | 1 | 2 | 0 | 0 |
| Southeast Alaska | 0.0 (0.2) | 0.0 (0.1) | 0.1 (0.4) | 0.0 (0.3) | 0.1 (1.1) | 0.0 (0.3) | 0.0 (0.5) | 0.0 (0.9) | 0.0 (0.3) |
| Alsek River | 0.0 (0.1) | 0.0 (0.0) | 0.0 (0.0) | 0.0 (0.1) | 0.0 (0.1) | 0.0 (0.1) | 0.0 (0.2) | 0.0 (0.4) | 0.0 (0.1) |
| Lower Stikine | 0.0 (0.1) | 0.0 (0.0) | 0.0 (0.1) | 0.0 (0.1) | 0.0 (0.3) | 0.1 (1.0) | 0.0 (0.6) | 0.0 (0.4) | 0.0 (0.3) |
| Lower Nass | 0.0 (0.1) | 0.1 (0.2) | 0.0 (0.1) | 0.0 (0.1) | 0.0 (0.2) | 0.0 (0.3) | 0.0 (0.4) | 0.0 (0.3) | 0.0 (0.3) |
| Upper Nass | 0.0 (0.0) | 0.0 (0.0) | 0.0 (0.0) | 1.0 (1.4) | 0.0 90.3) | 0.0 (0.3) | 0.0 90.1) | 0.0 (0.2) | 0.0 (0.0) |
| Portland Sound-Observatory Inlet-Portland Canal | 0.0 (0.1) | 0.0 (0.0) | 0.0 (0.0) | 0.0 (0.1) | 0.0 (0.1) | 0.0 (0.0) | 0.0 (0.3) | 0.0 (0.2) | 0.0 (0.0) |
| Skeena Estuary | 0.0 (0.1) | 0.0 (0.0) | 0.0 (0.1) | 0.0 (0.2) | 0.0 (0.2) | 0.0 (0.3) | 0.0 (0.3) | 0.0 (0.4) | 0.0 (0.1) |
| Lower Skeena | 0.0 (0.1) | 0.0 (0.1) | 1.0 (1.2) | 0.0 (0.2) | 0.0 (0.2) | 0.0 (0.4) | 0.0 (0.5) | 0.0 (0.4) | 0.0 (0.2) |
| Middle Skeena | 0.9 (0.9) | 0.0 (0.0) | 0.0 (0.1) | 1.9 (1.8) | 0.0 (0.2) | 0.0 (0.3) | 0.0 (0.4) | 0.0 (0.2) | 0.0 (0.2) |
| Upper Skeena | 0.0 (0.1) | 0.0 (0.0) | 0.0 (0.1) | 1.0 91.9) | 0.0 (0.2) | 0.0 (0.2) | 0.0 (0.2) | 0.0 (0.5) | 0.0 (0.1) |
| Haida Gwaii-Graham Island Lowlands | 0.0 (0.1) | 0.0 (0.0) | 0.0 (0.1) | 0.0 90.2) | 0.0 (0.4) | 0.0 (0.4) | 0.0 (1.0) | 0.0 (0.6) | 0.0 (0.2) |
| Haida Gwaii-East | 0.0 (0.1) | 0.0 (0.0) | 0.0 (0.1) | 0.0 (0.2) | 0.0 (0.3) | 0.0 (0.2) | 0.0 90.3) | 0.0 (0.30 | 0.0 (0.1) |
| Haida Gwaii-West | 0.0 (0.1) | 0.0 (0.0) | 0.0 (0.1) | 0.0 (0.2) | 0.0 (0.2) | 0.0 (0.2) | 0.0 (0.2) | 0.0 (0.5) | 0.0 (0.2) |
| Northern Coastal Streams | 6.2 (4.4) | 9.1 (1.8) | 22.8 (4.8) | 27.5 (5.7) | 14.5 (6.5) | 23.2 (8.0) | 6.3 (7.8) | 5.7 (7.1) | 34.4 (6.4) |
| Hecate Strait Mainland | 1.1 (1.3) | 6.9 (1.4) | 11.7 (4.2) | 12.9 (4.4) | 4.7 (3.5) | 21.6 (6.9) | 5.8 (6.8) | 5.0 (4.6) | 1.5 (2.3) |
| Mussel-Kynoch | 0.0 (0.1) | 0.0 (0.0) | 0.0 (0.0) | 0.0 (0.1) | 2.2 (2.0) | 0.0 (0.1) | 0.0 (0.0) | 0.0 (0.2) | 1.1 (1.1) |
| Douglas Channel-Kitimat Arm | 4.8 (5.5) | 10.0 (1.8) | 56.5 (5.4) | 23.3 (5.8) | 65.8 (8.4) | 16.3 (6.7) | 8.5 (8.4) | 6.8 (7.6) | 3.4 (3.0) |
| Bella Coola-Dean Rivers | 32.8 (5.3) | 32.1 (2.5) | 7.8 (3.3) | 21.2 (5.4) | 3.2 (4.0) | 13.1 (5.8) | 15.8 (8.3) | 14.9 (7.9) | 36.7 (6.0) |
| Rivers Inlet | 17.3 (4.2) | 20.3 (2.1) | 0.0 (0.1) | 3.2 (2.0) | 1.0 (1.7) | 0.0 (0.2) | 0.0 (0.5) | 5.5 (4.7) | 0.6 (1.4) |
| Smith Inlet | 2.9 (2.0) | 2.5 (0.9) | 0.0 (.1) | 2.6 (1.8) | 0.0 (0.2) | 3.6 (3.0) | 0.0 (0.3) | 16.5 (6.5) | 0.5 (1.1) |
| Southern Coastal Streams-Queen Charlotte Strait-Johnstone Strait-Southern Fjords | 18.6 (4.7) | 10.8 (1.9) | 0.0 (0.5) | 0.6 (0.9) | 7.8 (4.7) | 11.0 (5.6) | 33.9 (11.4) | 18.0 (7.5) | 0.2 (0.4) |
| Homathko-Klinaklini Rivers | 7.5 (3.2) | 2.7 (1.0) | 0.0 (0.1) | 0.5 (1.6) | 0.2 (0.8) | 0.0 (0.1) | 10.9 (6.6) | 0.0 (0.6) | 1.8 (3.5) |
| Georgia Strait Mainland | 0.0 (0.1) | 0.0 (0.0) | 0.0 (0.1) | 0.0 (0.1) | 0.0 (0.2) | 0.0 (0.1) | 0.0 (0.3) | 0.0 (0.2) | 0.0 (0.1) |
| Howe Sound-Burrard Inlet | 1.0 (2.6) | 1.2 (0.6) | 0.0 (0.2) | 2.4 (1.9) | 0.4 (1.3) | 7.8 (4.1) | 0.0 (0.9) | 3.5 (5.7) | 9.7 (4.0) |
| East Vancouver Island-Georgia Strait | 2.9 (1.8) | 1.6 (0.7) | 0.0 (0.2) | 1.3 (1.4) | 0.2 (1.1) | 1.2 (2.1) | 12.6 (5.7) | 7.7 (6.3) | 7.6 (3.5) |
| East Vancouver Island-Johnstone Strait-Southern Fjords | 0.0 (0.1) | 0.0 (0.0) | 0.0 (0.10 | 0.0 (0.3) | 0.0 (0.2) | 0.1 (0.5) | 0.0 (0.1) | 0.1 (0.5) | 0.0 (0.1) |
| Nahwitti Lowland | 2.2 (1.5) | 0.0 (0.1) | 0.1 (0.5) | 0.5 (1.3) | 0.0 (0.3) | 0.6 (1.2) | 3.1 (3.1) | 0.0 (0.6) | 0.1 (0.5) |
| West Vancouver Island | 0.0 (0.3) | 0.0 (0.0) | 0.0 (0.1) | 0.0 (0.2) | 0.0 (0.3) | 0.3 (1.4) | 3.1 (2.8) | 0.9 (3.1) | 0.8 (1.1) |
| Clayoquot | 0.0 (0.1) | 1.8 (0.8) | 0.0 (0.1) | 0.0 (0.3) | 0.0 (0.2) | 0.1 (0.9) | 0.0 (0.3) | 0.4 (2.2) | 1.4 (2.0) |
| Juan de Fuca-Pachena | 0.0 (0.1) | 0.0 (0.0) | 0.0 (0.1) | 0.0 (0.1) | 0.0 (0.2) | 0.0 (0.4) | 0.0 (0.2) | 9.1 (5.3) | 0.0 (0.1) |
| Lower Fraser | 0.0 (0.3) | 0.0 (0.1) | 0.0 (0.3) | 0.0 (0.4) | 0.0 (0.5) | 0.0 (0.6) | 0.0 (1.0) | 1.0 (2.6) | 0.0 (0.4) |
| Lillooet | 0.0 (0.2) | 0.9 (0.4) | 0.0 (0.1) | 0.0 (0.2) | 0.0 (0.2) | 0.0 (0.3) | 0.0 (0.4) | 3.3 (3.3) | 0.0 (0.1) |
| Fraser Canyon | 0.0 (0.0) | 0.0 (0.0) | 0.0 (0.1) | 0.0 (0.1) | 0.0 (0.3) | 0.0 (0.2) | 0.0 (0.3) | 0.0 (0.5) | 0.0 (0.1) |
| Interior Fraser | 0.0 (0.1) | 0.0 (0.0) | 0.0 (0.1) | 0.0 (0.1) | 0.0 (0.2) | 0.0 (0.3) | 0.0 (0.5) | 0.0 (0.5) | 0.0 (0.2) |
| Lower Thompson | 0.0 (0.0) | 0.0 (0.0) | 0.0 (0.1) | 0.0 (0.1) | 0.0 (0.1) | 0.0 (0.2) | 0.0 (0.2) | 0.0 (0.3) | 0.0 (0.0) |
| North Thompson | 0.9 (1.0) | 0.0 (0.1) | 0.0 (0.3) | 0.0 (0.3) | 0.0 (0.6) | 0.0 (0.6) | 0.0 (0.9) | 0.0 (0.7) | 0.0 (0.3) |
| South Thompson | 0.0 (0.2) | 0.0 (0.0) | 0.0 (0.2) | 0.0 (0.3) | 0.0 (0.5) | 0.0 (0.5) | 0.0 (0.8) | 0.0 (0.8) | 0.0 (0.3) |
| Boundary Bay | 0.0 (0.1) | 0.0 (0.0) | 0.0 (0.1) | 0.0 (0.1) | 0.0 (0.2) | 0.0 (0.2) | 0.0 (0.3) | 0.0 (0.3) | 0.0 (0.1) |
| Nooksack River | 0.1 (0.3) | 0.1 (0.2) | 0.0 (0.1) | 0.0 (0.1) | 0.0 (0.1) | 0.3 (2.3) | 0.1 (0.3) | 0.2 (0.2) | 0.0 (0.1) |
| Skagit River | 0.0 (0.1) | 0.0 (0.0) | 0.0 (0.1) | 0.0 (0.2) | 0.0 (0.2) | 0.1 (1.3) | 0.0 (0.4) | 0.0 (0.7) | 0.0 (0.3) |
| Northern Puget Sound | 0.6 (0.9) | 0.0 (0.1) | 0.0 (0.1) | 0.0 (0.2) | 0.0 (0.2) | 0.3 (0.6) | 0.0 (0.2) | 1.3 (5.2) | 0.0 (0.2) |
| Mid-Puget Sound | 0.2 (0.7) | 0.0 (0.0) | 0.0 (0.1) | 0.0 (0.1) | 0.0 (0.1) | 0.0 (0.3) | 0.0 (0.3) | 0.0 (0.2) | 0.0 (0.1) |
| Southern Puget Sound | 0.0 (0.1) | 0.0 (0.0) | 0.0 (0.1) | 0.0 (0.2) | 0.0 (0.2) | 0.1 (0.6) | 0.0 (0.3) | 0.0 (0.3) | 0.0 (0.1) |
| Juan de Fuca Strait | 0.0 (0.1) | 0.0 (0.0) | 0.0 (0.1) | 0.0 (0.1) | 0.0 (0.1) | 0.0 (0.1) | 0.0 (0.2) | 0.1 (0.5) | 0.0 (0.0) |
| Hood Canal | 0.0 (0.1) | 0.0 (0.0) | 0.0 (0.1) | 0.0 (0.2) | 0.0 (0.2) | 0.0 (0.1) | 0.0 (0.3) | 0.0 (0.1) | 0.0 (0.0) |
| Coastal Washington | 0.0 (0.1) | 0.0 (0.1) | 0.0 (0.2) | 0.0 (0.2) | 0.0 (0.3) | 0.0 (0.3) | 0.0 (0.5) | 0.0 (0.5) | 0.0 (0.2) |
| Columbia River | 0.0 (0.1) | 0.0 (0.0) | 0.0 (0.2) | 0.0 (0.2) | 0.0 (0.4) | 0.0 (0.3) | 0.0 (0.5) | 0.0 (0.7) | 0.0 (0.2) |
| Oregon | 0.0 (0.1) | 0.0 (0.0) | 0.0 (0.2) | 0.0 (0.2) | 0.0 (0.4) | 0.0 (0.5) | 0.0 (0.6) | 0.0 (0.6) | 0.0 (0.2) |

Supplementary Table 3 concluded.

| Region/Conservation Unit | Commercial troll | Round Island test | |
| --- | --- | --- | --- |
|  | Area 8-1 |  |  |
|  | Oct 1-7 | July | August |
| Sample size | 98 | 81 | 64 |
| N-PBT | 2 | 8 | 6 |
| Southeast Alaska | 1.1 (1.1) | 0.1 (0.7) | 0.1 (0.7) |
| Alsek River | 0.0 (0.1) | 0.0 (0.1) | 0.0 (0.1) |
| Lower Stikine | 0.0 (0.0) | 0.0 (0.1) | 0.0 (0.1) |
| Lower Nass | 0.0 (0.1) | 0.0 (0.1) | 0.0 (0.3) |
| Upper Nass | 0.1 (0.6) | 0.0 (0.1) | 0.0 (0.0) |
| Portland Sound-Observatory Inlet-Portland Canal | 0.0 (0.1) | 0.0 (0.1) | 0.0 (0.1) |
| Skeena Estuary | 0.0 (0.2) | 0.0 (0.1) | 0.0 (0.2) |
| Lower Skeena | 0.6 (0.9) | 0.0 (0.2) | 0.0 (0.2) |
| Middle Skeena | 0.0 (0.1) | 0.0 (0.2) | 0.0 (0.2) |
| Upper Skeena | 0.9 (1.0) | 0.0 (0.2) | 0.0 (0.1) |
| Haida Gwaii-Graham Island Lowlands | 1.0 (1.0) | 0.0 (0.2) | 0.0 (0.3) |
| Haida Gwaii-East | 0.0 (0.1) | 0.0 (0.1) | 0.0 (0.2) |
| Haida Gwaii-West | 0.0 (0.1) | 0.0 (0.2) | 0.0 (0.2) |
| Northern Coastal Streams | 14.9 (4.9) | 4.2 (4.1) | 0.7 (1.5) |
| Hecate Strait Mainland | 8.8 (4.2) | 0.6 (1.1) | 0.0 (0.4) |
| Mussel-Kynoch | 0.0 (0.1) | 0.0 (0.0) | 0.0 (0.1) |
| Douglas Channel-Kitimat Arm | 14.4 (4.7) | 2.9 (3.5) | 0.0 (0.4) |
| Bella Coola-Dean Rivers | 32.8 (5.5) | 5.1 (2.8) | 1.5 (2.4) |
| Rivers Inlet | 1.6 (1.8) | 2.8 (1.9) | 0.2 (0.9) |
| Smith Inlet | 1.0 (1.0) | 0.0 (0.1) | 0.0 (0.4) |
| Southern Coastal Streams-Queen Charlotte Strait-Johnstone Strait-Southern Fjords | 8.9 (3.8) | 11.5 (3.8) | 13.0 (4.5) |
| Homathko-Klinaklini Rivers | 5.9 (2.9) | 11.7 (3.9) | 15.3 (5.0) |
| Georgia Strait Mainland | 0.0 (0.1) | 0.0 (0.1) | 0.0 (0.1) |
| Howe Sound-Burrard Inlet | 0.3 (0.8) | 12.8 (4.0) | 5.1 (3.9) |
| East Vancouver Island-Georgia Strait | 4.7 (2.4) | 24.6 (5.0) | 38.8 (6.5) |
| East Vancouver Island-Johnstone Strait-Southern Fjords | 0.0 (0.1) | 0.0 (0.1) | 0.1 (0.6) |
| Nahwitti Lowland | 0.4 (1.1) | 8.6 (3.4) | 10.5 (4.0) |
| West Vancouver Island | 1.1 (1.1) | 3.7 (2.1) | 2.5 (2.8) |
| Clayoquot | 0.3 (0.7) | 0.0 (0.1) | 0.3 (1.0) |
| Juan de Fuca-Pachena | 0.0 (0.1) | 0.0 (0.2) | 0.1 (0.6) |
| Lower Fraser | 0.0 (0.3) | 4.0 (2.3) | 1.7 (1.7) |
| Lillooet | 0.0 (0.1) | 0.2 (0.6) | 0.0 (0.3) |
| Fraser Canyon | 0.0 (0.1) | 0.0 (0.1) | 0.0 (0.1) |
| Interior Fraser | 0.0 (0.1) | 0.0 (0.2) | 0.0 (0.2) |
| Lower Thompson | 0.0 (0.1) | 0.0 (0.1) | 0.0 (0.1) |
| North Thompson | 0.0 (0.3) | 0.0 (0.3) | 0.0 (0.4) |
| South Thompson | 0.0 (0.3) | 0.7 (1.2) | 1.6 (1.5) |
| Boundary Bay | 0.0 (0.1) | 1.2 (1.3) | 0.0 (0.1) |
| Nooksack River | 0.0 (0.0) | 0.0 (0.5) | 0.0 (0.3) |
| Skagit River | 0.0 (0.1) | 0.0 (0.2) | 1.3 (2.1) |
| Northern Puget Sound | 0.0 (0.1) | 0.1 (0.6) | 2.2 (3.2) |
| Mid-Puget Sound | 0.0 (0.1) | 3.8 (2.2) | 0.0 (0.2) |
| Southern Puget Sound | 0.0 (0.1) | 0.1 (0.3) | 0.1 (0.7) |
| Juan de Fuca Strait | 0.0 (0.1) | 0.0 (0.1) | 0.1 (0.7) |
| Hood Canal | 0.0 (0.2) | 0.0 (0.1) | 0.0 (0.1) |
| Coastal Washington | 0.7 (0.9) | 0.0 (0.2) | 3.1 (2.2) |
| Columbia River | 0.3 (0.8) | 1.2 (1.2) | 1.6 (1.5) |
| Oregon | 0.0 (0.2) | 0.0 (0.2) | 0.0 (0.3) |

Supplementary Table 4. Percentage stock composition by geographic region or CU of 2017 adipose fin-clipped fishery samples sent to a central laboratory for potential CWT recovery. The northern Area F ice boat troll fishery sample included only clipped individuals, some of whom may have displayed a CWT, and was supplemented by direct DNA sampling of clipped individuals in fishery landings. All individuals from recreational fishery samples were presumed adipose fin clipped, and some individuals would also be marked with a CWT. Seasonal values were obtained by weighting monthly samples by catch such that not all indivduals genotyped were included in the seasonal sample. N-PBT is the number of individuals identified in the sample via PBT.

| Region/Conservation Unit | Northern Area F troll (marked) | | | | Northern sport | | | |
| --- | --- | --- | --- | --- | --- | --- | --- | --- |
|  | July | August | Sept. | Seasonal | June | July | August | Seasonal |
| Sample size | 620 | 217 | 246 | 768 | 12 | 36 | 35 | 85 |
| N-PBT | 128 | 36 | 32 | 156 | 3 | 4 | 7 | 14 |
| Southeast Alaska | 3.6 (0.8) | 10.5 (2.2) | 7.5 (1.7) | 6.0 (0.8) | 0.0 (1.8) | 12.7 (5.5) | 2.6 (4.5) | 7.9 (3.1) |
| Alsek River | 0.0 (0.0) | 0.0 (0.0) | 0.0 (0.0) | 0.0 (0.0) | 0.0 (0.6) | 0.0 (0.1) | 0.0 (0.1) | 0.0 (0.1) |
| Lower Stikine | 0.2 (0.1) | 0.0 (0.1) | 0.0 (0.0) | 0.1 (0.1) | 0.0 (0.2) | 0.0 (0.3) | 0.2 (0.9) | 0.1 (0.9) |
| Lower Nass | 2.5 (0.7) | 5.6 (1.8) | 4.2 (1.4) | 3.4 (0.6) | 0.0 (0.5) | 2.8 (2.5) | 1.0 (2.3) | 3.8 (2.1) |
| Upper Nass | 1.4 (0.5) | 0.3 (0.5) | 0.3 (0.5) | 0.8 (0.3) | 0.0 (0.1) | 0.5 (4.1) | 3.2 (4.2) | 1.6 (2.5) |
| Portland Sound-Observatory Inlet-Portland Canal | 0.0 (0.0) | 0.0 (0.0) | 0.0 (0.0) | 0.0 (0.0) | 0.0 (0.3) | 0.0 (0.2) | 0.0 (0.1) | 0.0 (0.0) |
| Skeena Estuary | 0.0 (0.0) | 0.0 (0.0) | 0.0 (0.0) | 0.0 (0.0) | 0.0 (0.7) | 0.0 (0.3) | 0.0 (0.3) | 0.0 (0.2) |
| Lower Skeena | 1.5 (0.6) | 0.2 (0.6) | 0.8 (0.8) | 1.3 (0.4) | 8.1 (7.4) | 0.0 (0.2) | 7.5 (4.5) | 1.6 (1.5) |
| Middle Skeena | 1.6 (0.6) | 5.2 (1.6) | 0.1 (0.2) | 2.0 (0.5) | 0.0 (0.4) | 0.1 (1.5) | 8.8 (5.7) | 3.4 (2.5) |
| Upper Skeena | 1.5 (0.6) | 5.1 (1.7) | 0.7 (0.7) | 2.1 (0.5) | 0.0 (0.9) | 13.3 (6.0) | 8.1 (3.1) | 8.7 (3.6) |
| Haida Gwaii-Graham Island Lowlands | 2.4 (0.6) | 2.3 (1.0) | 1.8 (0.9) | 2.2 (0.4) | 0.0 (1.4) | 5.6 (3.6) | 0.0 (0.4) | 2.3 (1.5) |
| Haida Gwaii-East | 0.7 (0.4) | 0.0 (0.1) | 0.1 (0.4) | 0.4 (0.2) | 0.0 (1.0) | 0.0 (0.3) | 0.0 (0.4) | 0.0 (0.1) |
| Haida Gwaii-West | 0.1 (0.2) | 0.5 (0.5) | 0.0 (0.2) | 0.2 (0.2) | 0.0 (0.6) | 0.0 (0.3) | 0.0 (0.3) | 0.0 (0.1) |
| Northern Coastal Streams | 1.2 (0.5) | 0.0 (0.2) | 0.8 (1.1) | 0.5 (0.4) | 0.0 (1.9) | 7.1 (5.0) | 0.4 (0.9) | 1.6 (1.9) |
| Hecate Strait Mainland | 2.0 (0.6) | 1.9 (1.0) | 2.6 (1.2) | 2.4 (0.6) | 0.0 (1.4) | 0.7 (1.6) | 9.1 (5.7) | 4.5 (3.0) |
| Mussel-Kynoch | 0.2 (0.2) | 0.0 (0.0) | 0.0 (0.1) | 0.1 (0.1) | 0.0 (0.5) | 0.0 (0.1) | 0.0 (0.3) | 0.0 (0.0) |
| Douglas Channel-Kitimat Arm | 2.5 (0.8) | 2.7 (1.3) | 2.4 (1.3) | 2.2 (0.6) | 0.0 (1.2) | 5.4 (4.3) | 2.0 (2.9) | 2.7 (2.2) |
| Bella Coola-Dean Rivers | 1.7 (0.7) | 0.2 (0.4) | 0.7 (0.7) | 1.2 (0.4) | 2.8 (5.8) | 0.0 (0.3) | 0.8 (1.8) | 1.2 (1.3) |
| Rivers Inlet | 0.2 (0.2) | 0.0 (0.1) | 0.1 (0.3) | 0.1 (0.1) | 0.0 (1.0) | 0.0 (0.5) | 0.0 (0.3) | 0.0 (0.1) |
| Smith Inlet | 0.0 (0.0) | 0.0 (0.1) | 0.0 (0.1) | 0.0 (0.0) | 0.0 (0.6) | 0.0 (0.4) | 0.0 (0.5) | 0.0 (0.1) |
| Southern Coastal Streams-Queen Charlotte Strait-Johnstone Strait-Southern Fjords | 0.8 (0.4) | 0.4 (0.6) | 0.1 (0.2) | 0.7 (0.3) | 0.0 (0.9) | 0.0 (0.5) | 0.0 (0.4) | 0.0 (0.1) |
| Homathko-Klinaklini Rivers | 0.0 (0.0) | 0.0 (0.0) | 0.1 (0.3) | 0.0 (0.0) | 3.6 (6.0) | 0.0 (0.1) | 0.0 (0.3) | 0.6 (1.0) |
| Georgia Strait Mainland | 0.0 (0.0) | 0.0 (0.1) | 0.0 (0.0) | 0.0 (0.0) | 0.0 (0.6) | 0.0 (0.3) | 0.0 (0.3) | 0.0 (0.1) |
| Howe Sound-Burrard Inlet | 3.6 (0.8) | 0.5 (0.6) | 0.5 (0.4) | 2.3 (0.5) | 3.0 (5.4) | 0.0 (0.5) | 0.0 (0.5) | 0.2 (1.0) |
| East Vancouver Island-Georgia Strait | 5.7 (1.1) | 4.1 (1.6) | 3.4 (1.5) | 5.0 (0.8) | 17.0 (11.8) | 1.3 (3.1) | 5.7 (3.8) | 6.3 (2.9) |
| East Vancouver Island-Johnstone Strait-Southern Fjords | 0.0 (0.0) | 0.0 (0.0) | 0.0 (0.1) | 0.0 (0.0) | 0.1 (0.8) | 0.0 (0.3) | 0.0 (0.2) | 0.0 (0.1) |
| Nahwitti Lowland | 0.8 (0.4) | 0.5 (0.6) | 1.5 (0.9) | 0.9 (0.3) | 0.0 (1.0) | 12.3 (5.7) | 0.0 (0.5) | 4.4 (2.3) |
| West Vancouver Island | 12. 4 (1.3) | 10.1 (2.1) | 8.9 (1.8) | 11.1 (1.0) | 8.3 (7.2) | 8.3 (4.5) | 11.4 (5.2) | 10.2 (3.1) |
| Clayoquot | 0.0 (0.0) | 0.0 (0.0) | 0.0 (0.0) | 0.0 (0.1) | 0.0 (0.8) | 0.0 (0.3) | 4.9 (4.4) | 1.2 (1.3) |
| Juan de Fuca-Pachena | 2.8 (0.7) | 2.5 (1.2) | 3.1 (1.2) | 2.8 (0.5) | 10.2 (9.1) | 3.0 (2.8) | 5.3 (4.4) | 4.1 (2.2) |
| Lower Fraser | 0.6 (0.3) | 1.4 (0.7) | 0.8 (0.6) | 0.8 (0.3) | 0.1 (2.6) | 0.0 (0.9) | 0.0 (0.7) | 0.0 (0.4) |
| Lillooet | 0.0 (0.1) | 0.0 (0.1) | 0.0 (0.1) | 0.0 (0.0) | 0.0 (0.8) | 0.0 (0.3) | 0.0 (0.3) | 0.0 (0.2) |
| Fraser Canyon | 0.0 (0.0) | 0.0 (0.0) | 0.0 (0.0) | 0.0 (0.0) | 0.0 (0.5) | 0.0 (0.1) | 0.0 (0.2) | 0.0 (0.1) |
| Interior Fraser | 0.0 (0.0) | 0.0 (0.1) | 0.0 (0.1) | 0.0 (0.0) | 0.0 (0.8) | 0.0 (0.4) | 0.0 (0.3) | 0.0 (0.2) |
| Lower Thompson | 0.0 (0.0) | 0.0 (0.1) | 0.0 (0.0) | 0.0 (0.0) | 0.0 (0.7) | 0.0 (0.2) | 0.0 (0.2) | 0.0 (0.0) |
| North Thompson | 0.0 (0.0) | 0.0 (0.1) | 0.0 (0.1) | 0.0 (0.0) | 0.0 (2.1) | 0.0 (0.8) | 0.0 (0.8) | 0.0 (0.2) |
| South Thompson | 0.0 (0.0) | 0.0 (0.3) | 0.0 (0.1) | 0.0 (0.0) | 0.0 (1.3) | 0.0 (0.5) | 0.0 (0.7) | 0.0 (0.3) |
| Boundary Bay | 0.2 (0.2) | 0.0 (0.0) | 0.0 (0.0) | 0.1 (0.1) | 0.0 (0.7) | 0.0 (0.3) | 0.0 (0.3) | 0.0 (0.1) |
| Nooksack River | 0.0 (0.1) | 0.5 (1.2) | 0.5 (0.7) | 0.3 (0.4) | 0.1 (0.5) | 0.3 (0.4) | 0.0 (0.2) | 0.0 (0.2) |
| Skagit River | 3.0 (0.9) | 2.9 (1.6) | 0.1 (0.4) | 2.3 (0.6) | 0.0 (0.4) | 0.0 (0.1) | 0.0 (0.7) | 0.0 (0.3) |
| Northern Puget Sound | 8.1 (1.4) | 4.6 (1.9) | 4.1 (1.6) | 6.1 (1.0) | 0.3 (0.8) | 0.0 (0.3) | 0.0 (0.3) | 0.0 (0.3) |
| Mid-Puget Sound | 9.8 (1.4) | 10.3 (2.3) | 6.6 (1.9) | 8.9 (1.0) | 0.1 (0.9) | 15.2 (6.4) | 2.2 (3.1) | 7.0 (3.1) |
| Southern Puget Sound | 4.2 (1.1) | 2.0 (1.8) | 0.9 (1.1) | 3.6 (0.7) | 22.5 (11.4) | 0.0 (0.4) | 1.0 (1.9) | 3.6 (2.5) |
| Juan de Fuca Strait | 4.5 (0.9) | 4.8 (1.6) | 3.5 (1.2) | 4.3 (0.7) | 0.6 (1.9) | 0.0 (0.3) | 0.0 (0.3) | 0.0 (0.1) |
| Hood Canal | 2.6 (0.7) | 1.5 (0.9) | 0.0 (0.2) | 1.8 (0.4) | 0.0 (1.1) | 0.6 (1.4) | 0.0 (0.1) | 0.1 (0.4) |
| Coastal Washington | 14.4 (1.6) | 14.0 (2.4) | 41.9 (3.2) | 20.6 (1.3) | 6.4 (6.8) | 10.7 (4.9) | 14.3 (5.7) | 15.8 (3.9) |
| Columbia River | 3.2 (0.7) | 5.1 (1.5) | 2.1 (0.9) | 3.3 (0.5) | 16.7 (9.8) | 0.0 (0.7) | 11.4 (4.9) | 6.9 (2.6) |
| Oregon | 0.0 (0.0) | 0.0 (0.1) | 0.0 (0.1) | 0.0 (0.0) | 0.1 (2.5) | 0.0 (0.6) | 0.0 (0.5) | 0.0 (0.2) |

Supplementary Table 4 continued.

| Region/ Conservation Unit | Central coast sport | | | | Johnstone Strait sport | | | | |
| --- | --- | --- | --- | --- | --- | --- | --- | --- | --- |
|  | June | July | August | Seasonal | June | July | August | Sept. | Seasonal |
| Sample size | 5 | 13 | 9 | 27 | 11 | 105 | 144 | 61 | 192 |
| N-PBT | 3 | 5 | 5 | 13 | 6 | 62 | 87 | 29 | 115 |
| Southeast Alaska | 0.0 (3.3) | 0.0 (1.5) | 0.2 (2.6) | 0.0 (1.3) | 0.0 (1.8) | 0.0 (0.2) | 0.0 (0.1) | 0.0 (0.4) | 0.0 (0.0) |
| Alsek River | 0.0 (0.9) | 0.0 (0.6) | 0.0 (0.5) | 0.0 (0.2) | 0.0 (0.4) | 0.0 (0.0) | 0.0 (0.0) | 0.0 (0.1) | 0.0 (0.0) |
| Lower Stikine | 0.0 (0.7) | 0.0 (0.4) | 0.0 (0.4) | 0.0 (0.1) | 0.0 (0.4) | 0.0 (0.0) | 0.0 (0.0) | 0.0 (0.1) | 0.0 (0.0) |
| Lower Nass | 0.0 (1.0) | 0.0 (0.9) | 0.0 (0.7) | 0.0 (0.4) | 0.0 (0.4) | 0.0 (0.1) | 0.0 (0.3) | 0.0 (0.2) | 0.0 (0.1) |
| Upper Nass | 0.0 (0.6) | 0.0 (0.5) | 0.0 (0.7) | 0.0 (0.2) | 0.0 (0.5) | 0.0 (0.0) | 0.0 (0.0) | 0.0 (0.1) | 0.0 (0.0) |
| Portland Sound-Observatory Inlet-Portland Canal | 0.0 (0.8) | 0.0 (0.3) | 0.0 (0.7) | 0.0 (0.1) | 0.0 (0.3) | 0.0 (0.1) | 0.0 (0.1) | 0.0 (0.1) | 0.0 (0.0) |
| Skeena Estuary | 0.0 (1.3) | 0.0 (0.6) | 0.1 (1.4) | 0.0 (0.9) | 0.0 (0.8) | 0.0 (0.1) | 0.0 (0.1) | 0.0 (0.1) | 0.0 (0.0) |
| Lower Skeena | 0.0 (2.1) | 0.0 (0.6) | 0.2 (2.3) | 0.0 (0.4) | 0.0 (0.8) | 0.0 (0.1) | 0.0 (0.0) | 0.1 (0.8) | 0.0 (0.1) |
| Middle Skeena | 0.0 (1.0) | 0.0 (0.8) | 0.0 (1.1) | 0.0 (0.4) | 0.0 (0.9) | 0.0 (0.1) | 0.0 (0.1) | 0.0 (0.1) | 0.0 (0.0) |
| Upper Skeena | 0.0 (2.0) | 0.0 (1.1) | 0.0 (1.3) | 0.0 (0.5) | 0.0 (0.7) | 0.0 (0.1) | 0.0 (0.1) | 0.0 (0.2) | 0.0 (0.0) |
| Haida Gwaii-Graham Island Lowlands | 0.0 (4.3) | 0.0 (1.4) | 0.3 (1.8) | 0.1 (0.8) | 0.0 (1.2) | 0.0 (0.2) | 0.0 (0.1) | 0.0 (0.2) | 0.0 (0.0) |
| Haida Gwaii-East | 0.0 (2.4) | 0.0 (0.9) | 0.0 (1.2) | 0.0 (0.2) | 0.0 (1.1) | 0.0 (0.1) | 0.0 (0.1) | 0.0 (0.2) | 0.0 (0.0) |
| Haida Gwaii-West | 0.0 (2.0) | 0.0 (0.9) | 0.0 (1.8) | 0.0 (0.4) | 0.0 (1.4) | 0.0 (0.1) | 0.0 (0.1) | 0.0 (0.1) | 0.0 (0.0) |
| Northern Coastal Streams | 0.0 (4.2) | 0.0 (1.9) | 3.8 (6.3) | 0.4 (1.5) | 0.0 (1.8) | 0.0 (0.2) | 0.0 (0.2) | 0.1 (0.5) | 0.0 (0.1) |
| Hecate Strait Mainland | 0.0 (2.5) | 0.0 (1.0) | 3.4 (7.5) | 1.4 (3.0) | 0.0 (0.8) | 0.0 (0.2) | 0.0 (0.2) | 0.0 (0.3) | 0.0 (0.1) |
| Mussel-Kynoch | 0.0 (0.5) | 0.0 (0.3) | 0.9 (2.0) | 0.3 (1.4) | 0.0 (0.3) | 0.0 (0.0) | 0.0 (0.0) | 0.0 (0.1) | 0.0 (0.0) |
| Douglas Channel-Kitimat Arm | 0.0 (2.0) | 0.0 (0.7) | 3.1 (4.8) | 0.6 (2.1) | 0.0 (1.6) | 0.0 (0.1) | 0.1 (0.4) | 1.8 (2.0) | 0.8 (0.6) |
| Bella Coola-Dean Rivers | 0.0 (3.0) | 15.3 (9.1) | 6.1(12.7) | 10.8 (5.7) | 0.0 (1.1) | 0.0 (0.2) | 1.0 (1.1) | 0.0 (0.2) | 0.2 (0.3) |
| Rivers Inlet | 0.0 (2.3) | 0.0 (0.7) | 0.0 (1.2) | 0.0 (0.7) | 0.0 (0.6) | 0.0 (0.1) | 0.0 (0.1) | 0.0 (0.2) | 0.0 (0.0) |
| Smith Inlet | 0.0 (1.3) | 0.0 (0.8) | 0.0 (1.1) | 0.0 (0.3) | 0.0 (1.2) | 0.0 (0.1) | 0.0 (0.1) | 0.0 (0.2) | 0.0 (0.0) |
| Southern Coastal Streams-Queen Charlotte Strait-Johnstone Strait-Southern Fjords | 0.0 (2.7) | 0.0 (1.7) | 3.1 (7.6) | 0.3 (1.8) | 0.0 (1.2) | 0.0 (0.2) | 0.0 (0.2) | 0.0 (0.3) | 0.0 (0.1) |
| Homathko-Klinaklini Rivers | 0.0 (0.7) | 0.0 (0.5) | 0.0 (0.3) | 0.0 (0.1) | 0.0 (0.4) | 0.0 (0.1) | 0.0 (0.2) | 0.0 (0.0) | 0.0 (0.0) |
| Georgia Strait Mainland | 0.0 (1.4) | 0.0 (0.9) | 0.0 (0.5) | 0.0 (0.2) | 0.0 (0.9) | 0.0 (0.0) | 0.0 (0.0) | 0.0 (0.1) | 0.0 (0.0) |
| Howe Sound-Burrard Inlet | 22.1 (14.8) | 23.2 10.7) | 33.6 (13.8) | 27.3 (8.2) | 0.0 (1.7) | 13.7 (3.5) | 20.2 (3.5) | 11.2 (4.7) | 16.5 (2.2) |
| East Vancouver Island-Georgia Strait | 46.5 (19.2) | 15.9 (9.6) | 22.9 (11.9) | 24.5 (8.3) | 27.7 (13.0) | 33.3 (4.7) | 31.6 (4.0) | 25.5 (5.9) | 30.6 (2.7) |
| East Vancouver Island-Johnstone Strait-Southern Fjords | 0.0 (1.6) | 0.0 (0.3) | 0.0 (0.7) | 0.0 (0.4) | 0.1 (1.2) | 0.0 (0.5) | 0.0 (0.1) | 0.0 (0.1) | 0.0 (0.0) |
| Nahwitti Lowland | 0.1 (3.1) | 6.8 (6.8) | 0.0 (1.3) | 3.4 (3.6) | 0.0 (1.5) | 2.8 (1.7) | 1.6 (1.2) | 7.0 (3.6) | 2.6 (1.0) |
| West Vancouver Island | 0.0 (1.6) | 0.0 (1.0) | 0.0 (1.3) | 0.0 (0.4) | 9.5 (9.5) | 5.7 (2.2) | 6.3 (2.1) | 1.6 (1.7) | 5.5 (1.3) |
| Clayoquot | 0.0 (1.9) | 0.0 (0.6) | 0.0 (1.7) | 0.0 (0.2) | 0.0 (0.9) | 0.0 (0.1) | 0.0 (0.1) | 0.0 (0.1) | 0.0 (0.1) |
| Juan de Fuca-Pachena | 1.0 (2.7) | 7.7 (6.2) | 0.0 (0.7) | 6.8 (4.8) | 0.0 (0.4) | 0.0 (0.1) | 0.0 (0.1) | 0.0 (0.3) | 0.0 (0.0) |
| Lower Fraser | 0.3 (5.3) | 0.0 (1.9) | 11.2 (9.1) | 4.0 (4.0) | 19.6 (11.4) | 15.1 (3.5) | 16.0 (3.2) | 24.1 (5.6) | 17.3 (2.1) |
| Lillooet | 0.0 (3.3) | 0.0 (1.0) | 0.0 (1.2) | 0.0 (0.4) | 0.0 (1.3) | 0.0 (0.1) | 0.0 (0.1) | 0.0 (0.2) | 0.0 (0.0) |
| Fraser Canyon | 0.0 (1.3) | 0.0 (1.0) | 0.0 (0.8) | 0.0 (0.1) | 0.0 (1.0) | 0.0 (0.1) | 0.0 (0.1) | 0.1 (0.6) | 0.0 (0.0) |
| Interior Fraser | 0.0 (1.9) | 0.0 (0.8) | 0.0 (1.2) | 0.0 (0.5) | 0.0 (0.7) | 0.0 (0.1) | 0.0 (0.1) | 0.0 (0.1) | 0.0 (0.0) |
| Lower Thompson | 0.0 (1.8) | 0.0 (0.4) | 0.0 (0.9) | 0.0 (0.2) | 0.0 (0.9) | 0.0 (0.1) | 0.0 (0.1) | 0.0 (0.1) | 0.0 (0.0) |
| North Thompson | 0.0 (3.4) | 0.0 (2.0) | 0.0 (1.6) | 0.0 (0.8) | 0.0 (2.3) | 0.0 (0.3) | 0.0 (0.2) | 0.0 (0.4) | 0.0 (0.1) |
| South Thompson | 0.9 (7.0) | 0.0 (1.6) | 0.0 (2.4) | 0.0 (0.7) | 0.0 (2.5) | 0.0 (0.2) | 0.0 (0.2) | 0.2 (1.6) | 0.0 (0.1) |
| Boundary Bay | 0.0 (1.7) | 0.0 (0.8) | 0.0 (0.8) | 0.0 (0.5) | 0.1 (0.8) | 0.9 (1.0) | 0.0 (0.1) | 1.6 (0.1) | 0.5 (0.3) |
| Nooksack River | 25.5 (20.2) | 0.3 (3.9) | 0.1 (1.4) | 1.1 (6.8) | 0.1 (2.9) | 0.0 (0.1) | 0.1 (0.6) | 0.0 (0.4) | 0.1 (0.1) |
| Skagit River | 0.1 (2.0) | 0.1 (0.7) | 0.9 (0.9) | 0.1 (2.4) | 4.1 (10.6) | 5.6 (2.7) | 7.5 (2.7) | 10.2 (4.4) | 7.6 (2.0) |
| Northern Puget Sound | 0.5 (3.0) | 15.3 (9.9) | 9.0 (8.9) | 11.1 (6.8) | 19.0 (14.1) | 11.4 (3.7) | 9.7 (3.3) | 0.6 (2.6) | 7.5 (2.2) |
| Mid-Puget Sound | 0.8 (1.5) | 0.0 (0.6) | 1.0 (1.8) | 0.3 (0.6) | 0.3 (4.1) | 4.4 (2.4) | 3.3 (1.7) | 0.7 (1.0) | 3.3 (1.3) |
| Southern Puget Sound | 2.1 (4.6) | 0.7 (3.4) | 0.1 (1.6) | 0.5 (1.7) | 1.2 (1.3) | 0.0 (0.1) | 0.4 (1.0) | 14.9 (5.5) | 3.6 (1.5) |
| Juan de Fuca Strait | 0.0 (1.4) | 0.0 (0.5) | 0.0 (0.8) | 0.0 (0.7) | 0.0 (0.7) | 1.0 (1.0) | 0.0 (0.1) | 0.0 (0.1) | 0.3 (0.3) |
| Hood Canal | 0.0 (0.9) | 0.0 (0.6) | 0.0 (1.0) | 0.0 (0.2) | 0.0 (0.5) | 0.0 (0.1) | 0.0 (0.3) | 0.0 (0.1) | 0.0 (0.0) |
| Coastal Washington | 0.0 (2.0) | 7.0 (6.5) | 0.0 (0.9) | 3.0 (3.5) | 0.0 (1.9) | 4.9 (2.1) | 1.4 (1.0) | 0.0 (0.2) | 2.2 (0.9) |
| Columbia River | 0.0 (2.7) | 7.7 (6.6) | 0.0 (2.5) | 3.7 (3.7) | 18.2 (10.1) | 1.0 (1.0) | 0.7 (0.7) | 0.0 (0.2) | 1.2 (0.6) |
| Oregon | 0.0 (3.0) | 0.1 (2.4) | 0.0 (2.3) | 0.0 (1.1) | 0.0 (1.8) | 0.0 (0.2) | 0.0 (0.2) | 0.0 (0.3) | 0.0 (0.0) |

Supplementary Table 4 continued.

| Region/Conservation Unit | Strait of Georgia sport | | | | | | Juan de Fuca Strait sport | | |
| --- | --- | --- | --- | --- | --- | --- | --- | --- | --- |
|  | June | July | August | Sept. | Oct. | Seasonal | June | July | August |
| Sample size | 42 | 96 | 68 | 54 | 8 | 171 | 15 | 20 | 59 |
| N-PBT | 30 | 57 | 48 | 48 | 8 | 120 | 6 | 4 | 20 |
| Southeast Alaska | 0.0 (0.4) | 0.0 (0.2) | 0.0 (0.2) | 0.0 (0.3) | 0.0 (1.9) | 0.0 (0.1) | 0.0 (1.1) | 0.0 (0.8) | 0.0 (0.3) |
| Alsek River | 0.0 (0.2) | 0.0 (0.0) | 0.0 (0.1) | 0.0 (0.1) | 0.0 (1.1) | 0.0 (0.0) | 0.0 (0.2) | 0.0 (0.1) | 0.0 (0.2) |
| Lower Stikine | 0.0 (0.1) | 0.0 (0.0) | 0.0 (0.1) | 0.0 (0.1) | 0.0 (0.7) | 0.0 (0.0) | 0.0 (0.6) | 0.0 (0.4) | 0.0 (0.1) |
| Lower Nass | 0.0 (0.2) | 0.0 (0.1) | 0.0 (0.1) | 0.0 (0.2) | 0.0 (0.6) | 0.0 (0.0) | 0.0 (1.0) | 0.0 (0.4) | 0.0 (0.1) |
| Upper Nass | 0.0 (0.1) | 0.0 (0.0) | 0.0 (0.1) | 0.0 (0.1) | 0.0 (0.7) | 0.0 (0.0) | 0.0 (0.3) | 0.0 (0.2) | 0.0 (0.1) |
| Portland Sound-Observatory Inlet-Portland Canal | 0.0 (0.2) | 0.0 (0.0) | 0.0 (0.1) | 0.0 (0.1) | 0.0 (0.6) | 0.0 (0.0) | 0.0 (0.3) | 0.0 (0.2) | 0.0 (0.1) |
| Skeena Estuary | 0.0 (0.2) | 0.0 (0.1) | 0.0 (0.1) | 0.0 (0.1) | 0.0 (1.5) | 0.0 (0.0) | 0.0 (0.4) | 0.0 (0.5) | 0.0 (0.2) |
| Lower Skeena | 0.0 (0.2) | 0.0 (0.1) | 0.0 (0.2) | 0.0 (0.2) | 0.0 (1.6) | 0.0 (0.0) | 0.0 (0.5) | 0.0 (0.7) | 0.0 (0.1) |
| Middle Skeena | 0.0 (0.1) | 0.0 (0.1) | 0.0 (0.2) | 0.0 (0.2) | 0.0 (1.2) | 0.0 (0.1) | 0.0 (0.6) | 0.0 (0.4) | 0.0 (0.1) |
| Upper Skeena | 0.0 (0.2) | 0.0 (0.1) | 0.0 (0.2) | 0.0 (0.1) | 0.0 (1.2) | 0.0 (0.0) | 0.0 (0.8) | 0.0 (0.5) | 0.0 (0.2) |
| Haida Gwaii-Graham Island Lowlands | 0.0 (0.4) | 0.0 (0.2) | 0.0 (0.3) | 0.0 (0.5) | 0.0 (2.5) | 0.0 (0.1) | 0.0 (0.8) | 0.0 (1.3) | 0.0 (0.2) |
| Haida Gwaii-East | 0.0 (0.3) | 0.0 (0.1) | 0.0 (0.1) | 0.0 (0.2) | 0.0 (1.1) | 0.0 (0.1) | 0.0 (0.7) | 0.0 (1.0) | 0.0 (0.2) |
| Haida Gwaii-West | 0.0 (0.3) | 0.0 (0.1) | 0.0 (0.2) | 0.0 (0.2) | 0.0 (1.2) | 0.0 (0.0) | 0.0 (0.7) | 0.0 (0.4) | 0.0 (0.1) |
| Northern Coastal Streams | 0.0 (0.6) | 0.0 (0.3) | 0.0 (0.3) | 0.0 (0.4) | 0.0 (2.7) | 0.0 (0.1) | 0.0 (1.6) | 0.0 (1.5) | 0.0 (0.4) |
| Hecate Strait Mainland | 0.0 (0.3) | 0.0 (0.1) | 0.0 (0.2) | 0.0 (0.3) | 0.0 (1.4) | 0.0 (0.0) | 0.0 (0.8) | 0.0 (0.6) | 0.0 (0.3) |
| Mussel-Kynoch | 0.0 (0.2) | 0.0 (0.0) | 0.0 (0.1) | 0.0 (0.1) | 0.0 (0.6) | 0.0 (0.0) | 0.0 (0.4) | 0.0 (0.2) | 0.0 (0.1) |
| Douglas Channel-Kitimat Arm | 0.0 (0.4) | 0.0 (0.1) | 0.0 (0.3) | 0.0 (0.1) | 0.0 (1.6) | 0.0 (0.0) | 0.0 (0.6) | 0.0 (0.4) | 0.0 (0.1) |
| Bella Coola-Dean Rivers | 0.0 (0.6) | 0.0 (0.2) | 0.0 (0.3) | 0.0 (0.4) | 0.0 (1.8) | 0.0 (0.1) | 0.0 (1.3) | 0.0 (0.7) | 0.0 (0.3) |
| Rivers Inlet | 0.0 (0.2) | 0.0 (0.1) | 0.0 (0.2) | 0.0 (0.4) | 0.0 (1.7) | 0.0 (0.1) | 0.0 (1.2) | 0.0 (0.6) | 0.0 (0.2) |
| Smith Inlet | 0.0 (0.2) | 0.0 (0.2) | 0.0 (0.1) | 0.0 (0.2) | 0.0 (1.4) | 0.0 (0.0) | 0.0 (0.9) | 0.0 (0.5) | 0.0 (0.2) |
| Southern Coastal Streams-Queen Charlotte Strait-Johnstone Strait-Southern Fjords | 0.0 (0.3) | 0.0 (0.4) | 0.0 (0.2) | 0.0 (0.3) | 0.0 (1.5) | 0.0 (0.0) | 0.0 (0.8) | 0.0 (0.6) | 0.0 (0.3) |
| Homathko-Klinaklini Rivers | 0.0 (0.1) | 0.0 (0.0) | 0.0 (0.0) | 0.0 (0.0) | 0.0 (0.8) | 0.0 (0.0) | 0.0 (0.4) | 0.0 (0.1) | 0.0 (0.1) |
| Georgia Strait Mainland | 0.0 (0.1) | 0.0 (0.1) | 0.0 (0.1) | 0.0 (0.2) | 0.0 (0.6) | 0.0 (0.0) | 0.0 (0.6) | 0.0 (0.2) | 0.0 (0.1) |
| Howe Sound-Burrard Inlet | 26.7 (6.6) | 28.3 (4.7) | 34.0 (5.8) | 55.2 (6.8) | 50.0 (16.2) | 36.0 (3.1) | 13.3 (8.3) | 15.7 (7.7) | 18.2 (4.9) |
| East Vancouver Island-Georgia Strait | 17.7 (6.2) | 9.3 (3.1) | 16.6 (4.7) | 11.2 (4.2) | 12.5 (10.0) | 12.3 (2.2) | 7.8 (6.6) | 5.0 (4.9) | 7.6 (3.8) |
| East Vancouver Island-Johnstone Strait-Southern Fjords | 0.0 (0.3) | 0.0 (0.2) | 0.0 (0.1) | 0.0 (0.1) | 0.0 (0.5) | 0.0 (0.0) | 0.0 (0.3) | 0.0 (0.5) | 0.0 (0.1) |
| Nahwitti Lowland | 0.0 (0.4) | 0.0 (0.2) | 0.1 (0.4) | 0.0 (0.3) | 0.0 (1.7) | 0.0 (0.1) | 0.0 (1.1) | 0.0 (1.2) | 0.0 (0.4) |
| West Vancouver Island | 0.0 (0.3) | 0.0 (0.2) | 0.0 (0.2) | 1.9 (1.8) | 0.0 (0.7) | 0.4 (0.4) | 0.0 (0.9) | 0.0 (0.3) | 1.7 (1.7) |
| Clayoquot | 0.0 (0.1) | 0.0 (0.1) | 0.0 (0.5) | 0.0 (0.2) | 0.0 (0.5) | 0.0 (0.0) | 0.0 (0.7) | 0.0 (0.5) | 0.0 (0.2) |
| Juan de Fuca-Pachena | 0.0 (0.2) | 0.0 (0.1) | 0.0 (0.1) | 0.0 (0.1) | 0.0 (0.7) | 0.0 (0.0) | 0.0 (0.6) | 0.0 (0.3) | 3.4 (2.3) |
| Lower Fraser | 27.2 (6.9) | 36.9 (4.9) | 34.1 (5.7) | 24.8 (5.5) | 37.5 (15.3) | 31.9 (2.8) | 13.7 (8.5) | 0.0 (1.5) | 6.8 (3.3) |
| Lillooet | 0.0 (0.4) | 0.0 (0.1) | 0.0 (0.2) | 0.0 (0.2) | 0.0 (1.7) | 0.0 (0.0) | 0.0 (0.8) | 0.0 (0.7) | 0.0 (0.2) |
| Fraser Canyon | 0.0 (0.2) | 0.0 (0.1) | 0.0 (0.1) | 0.0 (0.2) | 0.0 (0.8) | 0.0 (0.0) | 0.0 (0.5) | 0.0 (0.3) | 0.0 (0.1) |
| Interior Fraser | 0.0 (0.2) | 0.0 (0.1) | 0.0 (0.2) | 0.0 (0.2) | 0.0 (1.2) | 0.0 (0.0) | 0.0 (0.6) | 0.0 (0.7) | 0.0 (0.2) |
| Lower Thompson | 4.8 (3.1) | 0.0 (0.1) | 0.0 (0.1) | 0.0 (0.1) | 0.0 (1.0) | 0.7 (0.5) | 0.0 (0.4) | 0.0 (0.4) | 0.0 (0.1) |
| North Thompson | 0.0 (0.6) | 0.0 (0.3) | 0.0 (0.3) | 0.0 (0.5) | 0.0 (2.7) | 0.0 (0.1) | 0.0 (1.4) | 0.0 (1.0) | 0.0 (0.4) |
| South Thompson | 2.4 (2.4) | 0.0 (0.3) | 0.0 (0.3) | 0.0 (0.4) | 0.0 (2.1) | 0.4 (0.4) | 0.0 (1.4) | 0.0 (1.1) | 3.4 (2.4) |
| Boundary Bay | 2.7 (2.6) | 1.7 (1.3) | 0.1 (0.5) | 1.9 (1.7) | 0.0 (1.1) | 1.4 (0.7) | 6.7 (4.6) | 0.0 (0.5) | 1.7 (1.0) |
| Nooksack River | 0.6 (0.8) | 1.1 (1.9) | 0.0 (0.1) | 0.0 (0.2) | 0.0 (1.3) | 0.2 (0.1) | 1.1 (5.0) | 18.4 (9.9) | 0.0 (0.1) |
| Skagit River | 11.0 (5.2) | 8.8 (3.3) | 6.2 (3.5) | 0.0 (0.6) | 0.0 (0.7) | 6.2 (1.7) | 0.2 (1.5) | 0.2 (1.9) | 11.6 (5.0) |
| Northern Puget Sound | 0.0 (0.1) | 5.0 (3.4) | 8.9 (3.7) | 4.8 (3.8) | 0.0 (1.1) | 6.5 (2.0) | 0.4 (1.6) | 22.4 (9.8) | 23.2 (6.3) |
| Mid-Puget Sound | 0.0 (0.4) | 3.0 (3.1) | 0.0 (0.3) | 0.3 (1.9) | 0.0 (0.9) | 0.9 (1.4) | 41.2 (14.2) | 5.2 (5.3) | 15.7 (5.7) |
| Southern Puget Sound | 6.9 (4.4) | 5.8 (2.6) | 0.0 (0.1) | 0.0 (0.4) | 0.0 (1.1) | 3.1 (1.3) | 14.7 (9.2) | 22.9 (11.8) | 0.1 (0.3) |
| Juan de Fuca Strait | 0.0 (0.1) | 0.0 (0.3) | 0.0 (0.1) | 0.0 (0.1) | 0.0 (0.7) | 0.0 (0.0) | 0.4 (1.2) | 0.0 (0.4) | 0.5 (1.3) |
| Hood Canal | 0.0 (0.4) | 0.0 (0.1) | 0.0 (0.2) | 0.0 (0.1) | 0.0 (0.9) | 0.0 (0.0) | 0.2 (3.0) | 0.0 (0.3) | 0.0 (0.2) |
| Coastal Washington | 0.0 (0.4) | 0.0 (0.2) | 0.0 (0.2) | 0.0 (0.4) | 0.0 (1.3) | 0.0 (0.0) | 0.0 (1.3) | 5.0 (4.9) | 4.4 (2.7) |
| Columbia River | 0.0 (0.3) | 0.0 (0.2) | 0.0 (0.2) | 0.0 (0.3) | 0.0 (1.6) | 0.0 (0.1) | 0.0 (1.4) | 5.0 (4.8) | 1.7 (1.7) |
| Oregon | 0.0 (0.4) | 0.0 (0.2) | 0.0 (0.3) | 0.0 (0.3) | 0.0 (1.9) | 0.0 (0.1) | 0.0 (1.1) | 0.1 (1.5) | 0.0 (0.3) |

Supplementary Table 4 continued.

| Region/Conservation Unit | Juan de Fuca Strait sport | | | West coast Vancouver Island sport | | | | |
| --- | --- | --- | --- | --- | --- | --- | --- | --- |
|  | September | October | Seasonal | June | July | August | September | Seasonal |
| Sample size | 263 | 73 | 374 | 23 | 205 | 267 | 22 | 517 |
| N-PBT | 91 | 53 | 148 | 2 | 33 | 56 | 8 | 99 |
| Southeast Alaska | 0.0 (0.1) | 0.0 (0.2) | 0.0 (0.0) | 0.0 (0.6) | 0.0 (0.1) | 0.0 (0.1) | 0.0 (0.9) | 0.0 (0.0) |
| Alsek River | 0.0 (0.0) | 0.0 (0.1) | 0.0 (0.0) | 0.0 (0.5) | 0.0 (0.0) | 0.0 (0.0) | 0.0 (0.1) | 0.0 (0.0) |
| Lower Stikine | 0.0 (0.0) | 0.0 (0.1) | 0.0 (0.0) | 0.0 (0.0) | 0.0 (0.0) | 0.0 (0.0) | 0.0 (0.1) | 0.0 (0.0) |
| Lower Nass | 0.0 (0.0) | 0.0 (0.1) | 0.0 (0.0) | 0.0 (0.4) | 0.0 (0.0) | 0.0 (0.0) | 0.0 (0.2) | 0.0 (0.1) |
| Upper Nass | 0.0 (0.0) | 0.0 (0.0) | 0.0 (0.0) | 0.0 (0.2) | 0.0 (0.0) | 0.0 (0.0) | 0.0 (0.1) | 0.0 (0.0) |
| Portland Sound-Observatory Inlet-Portland Canal | 0.0 (0.0) | 0.0 (0.1) | 0.0 (0.0) | 0.0 (0.6) | 0.0 (0.0) | 0.0 (0.0) | 0.0 (0.2) | 0.0 (0.0) |
| Skeena Estuary | 0.0 (0.0) | 0.0 (0.1) | 0.0 (0.0) | 0.0 (0.6) | 0.0 (0.0) | 0.0 (0.0) | 0.0 (0.6) | 0.0 (0.0) |
| Lower Skeena | 0.0 (0.1) | 0.0 (0.1) | 0.0 (0.0) | 0.1 (0.8) | 0.0 (0.1) | 0.0 (0.0) | 0.0 (0.7) | 0.0 (0.0) |
| Middle Skeena | 0.0 (0.0) | 0.0 (0.1) | 0.0 (0.0) | 0.1 (0.4) | 0.0 (0.0) | 0.0 (0.0) | 0.0 (0.7) | 0.0 (0.0) |
| Upper Skeena | 0.0 (0.0) | 0.0 (0.2) | 0.0 (0.0) | 0.0 (0.4) | 0.0 (0.1) | 0.0 (0.1) | 0.0 (0.3) | 0.0 (0.0) |
| Haida Gwaii-Graham Island Lowlands | 0.0 (0.1) | 0.0 (0.3) | 0.0 (0.1) | 0.0 (0.8) | 0.0 (0.1) | 0.0 (0.1) | 0.0 (0.7) | 0.0 (0.0) |
| Haida Gwaii-East | 0.0 (0.0) | 0.0 (0.2) | 0.0 (0.0) | 0.0 (0.6) | 0.0 (0.1) | 0.0 (0.0) | 0.0 (0.5) | 0.0 (0.0) |
| Haida Gwaii-West | 0.0 (0.0) | 0.0 (0.2) | 0.0 (0.0) | 0.0 (0.4) | 0.0 (0.1) | 0.0 (0.0) | 0.0 (0.7) | 0.0 (0.0) |
| Northern Coastal Streams | 0.0 (0.1) | 0.0 (0.3) | 0.0 (0.1) | 0.2 (1.5) | 0.1 (0.3) | 0.0 (0.1) | 0.0 (1.2) | 0.0 (0.1) |
| Hecate Strait Mainland | 0.0 (0.1) | 0.0 (0.2) | 0.0 (0.1) | 0.0 (0.8) | 0.0 (0.1) | 0.0 (0.1) | 0.0 (0.8) | 0.0 (0.1) |
| Mussel-Kynoch | 0.0 (0.0) | 0.0 (0.2) | 0.0 (0.0) | 0.0 (0.2) | 0.0 (0.0) | 0.0 (0.0) | 0.0 (0.2) | 0.0 (0.0) |
| Douglas Channel-Kitimat Arm | 0.0 (0.1) | 0.0 (0.2) | 0.0 (0.0) | 0.0 (0.6) | 0.0 (0.1) | 0.0 (0.1) | 0.0 (0.8) | 0.0 (0.1) |
| Bella Coola-Dean Rivers | 0.0 (0.0) | 0.0 (0.2) | 0.0 (0.0) | 0.0 (0.9) | 0.5 (0.5) | 0.0 (0.0) | 0.0 (0.4) | 0.2 (0.2) |
| Rivers Inlet | 0.0 (0.1) | 0.0 (0.2) | 0.0 (0.0) | 0.0 (0.9) | 0.0 (0.2) | 0.0 (0.0) | 0.0 (0.6) | 0.0 (0.0) |
| Smith Inlet | 0.0 (0.1) | 0.0 (0.1) | 0.0 (0.0) | 0.0 (0.6) | 0.0 (0.1) | 0.0 (0.0) | 0.0 (0.3) | 0.0 (0.0) |
| Southern Coastal Streams-Queen Charlotte Strait-Johnstone Strait-Southern Fjords | 0.0 (0.1) | 0.0 (0.2) | 0.0 (0.0) | 0.0 (0.6) | 0.6 (0.7) | 0.0 (0.1) | 0.0 (0.5) | 0.2 (0.2) |
| Homathko-Klinaklini Rivers | 0.0 (0.1) | 0.0 (0.1) | 0.0 (0.1) | 0.0 (0.8) | 0.9 (0.7) | 0.0 (0.0) | 0.0 (0.2) | 0.4 (0.3) |
| Georgia Strait Mainland | 0.0 (0.0) | 0.0 (0.1) | 0.0 (0.0) | 0.0 (0.2) | 0.0 (0.0) | 0.0 (0.0) | 0.0 (0.5) | 0.0 (0.0) |
| Howe Sound-Burrard Inlet | 6.8 (1.6) | 11.0 (3.6) | 9.4 (1.5) | 0.1 (1.7) | 3.0 (1.3) | 2.1 (0.7) | 0.0 (1.2) | 2.3 (0.7) |
| East Vancouver Island-Georgia Strait | 5.9 (1.5) | 10.7 (3.8) | 7.7 (1.5) | 1.5 (2.8) | 10.5 (2.5) | 4.6 (1.6) | 11.4 (8.3) | 6.8 (1.3) |
| East Vancouver Island-Johnstone Strait-Southern Fjords | 0.0 (0.0) | 0.0 (0.2) | 0.0 (0.0) | 0.0 (0.5) | 0.0 (0.1) | 0.0 (0.0) | 0.0 (0.6) | 0.0 (0.0) |
| Nahwitti Lowland | 0.0 (0.1) | 0.0 (0.2) | 0.0 (0.0) | 13.4 (7.0) | 4.0 (1.7) | 2.8 (1.1) | 0.0 (0.9) | 3.6 (0.9) |
| West Vancouver Island | 0.0 (0.1) | 0.0 (0.1) | 0.2 (0.3) | 4.5 (4.2) | 6.7 (1.7) | 10.4 (1.9) | 17.9 (8.1) | 9.3 (1.3) |
| Clayoquot | 0.0 (0.0) | 0.0 (0.2) | 0.0 (0.0) | 0.0 (1.2) | 0.8 (1.3) | 0.0 (0.0) | 0.0 (0.3) | 0.2 (0.5) |
| Juan de Fuca-Pachena | 0.8 (0.5) | 0.0 (0.1) | 0.9 (0.5) | 0.0 (0.3) | 2.0 (1.1) | 4.2 (1.3) | 4.5 (3.4) | 3.1 (0.8) |
| Lower Fraser | 19.6 (2.4) | 67.5 (5.5) | 24.9 (2.1) | 10.0 (6.5) | 5.9 (1.6) | 6.6 (1.5) | 9.1 (6.0) | 6.7 (1.2) |
| Lillooet | 0.0 (0.1) | 0.0 (0.2) | 0.0 (0.0) | 0.0 (0.7) | 0.0 (0.1) | 0.0 (0.1) | 0.0 (0.4) | 0.1 (0.1) |
| Fraser Canyon | 0.0 (0.0) | 0.0 (0.2) | 0.0 (0.0) | 0.0 (0.5) | 0.0 (0.0) | 0.0 (0.0) | 0.0 (0.7) | 0.0 (0.0) |
| Interior Fraser | 0.0 (0.0) | 0.0 (0.2) | 0.0 (0.0) | 0.0 (0.6) | 0.0 (0.1) | 0.0 (0.0) | 0.0 (0.5) | 0.0 (0.0) |
| Lower Thompson | 1.9 (0.8) | 0.0 (0.1) | 1.2 (0.5) | 0.0 (0.4) | 0.0 (0.0) | 0.4 (0.4) | 0.0 (0.4) | 0.2 (0.2) |
| North Thompson | 0.0 (0.1) | 0.0 (0.2) | 0.0 (0.1) | 0.0 (1.0) | 0.0 (0.1) | 0.0 (0.1) | 0.0 (1.1) | 0.0 (0.1) |
| South Thompson | 0.8 (0.6) | 0.0 (0.4) | 0.9 (0.5) | 0.0 (1.1) | 0.0 (0.1) | 0.0 (0.1) | 0.0 (1.0) | 0.0 (0.0) |
| Boundary Bay | 3.4 (1.1) | 1.4 (1.3) | 2.7 (0.7) | 0.0 (0.5) | 0.5 (0.5) | 0.4 (0.0) | 0.0 (0.6) | 0.4 (0.2) |
| Nooksack River | 1.2 (1.8) | 0.5 (1.1) | 2.7 (1.1) | 5.0 (9.4) | 0.1 (0.1) | 0.4 (1.0) | 0.0 (0.5) | 1.0 (1.2) |
| Skagit River | 5.3 (1.7) | 0.2 (1.0) | 4.4 (1.2) | 4.2 (6.1) | 5.8 (1.9) | 6.5 (2.0) | 0.1 (0.7) | 5.6 (1.3) |
| Northern Puget Sound | 23.0 (3.3) | 0.5 (1.1) | 16.9 (2.3) | 38.3 (11.9) | 10.4 (3.1) | 9.5 (2.5) | 18.0 (7.7) | 10.9 (1.9) |
| Mid-Puget Sound | 12.7 (2.6) | 0.0 (0.1) | 11.1 (1.8) | 0.0 (0.6) | 14.6 (2.9) | 19.7 (2.7) | 10.9 (6.7) | 15.4 (1.8) |
| Southern Puget Sound | 13.2 (2.7) | 0.0 (0.5) | 10.4 (1.8) | 0.0 (0.8) | 11.0 (3.1) | 13.7 (2.5) | 0.0 (0.6) | 12.4 (1.8) |
| Juan de Fuca Strait | 1.8 (0.8) | 6.9 (3.0) | 2.9 (0.9) | 9.5 (7.0) | 4.7 (1.5) | 0.6 (0.9) | 0.1 (0.5) | 3.2 (0.9) |
| Hood Canal | 0.0 (0.1) | 0.0 (0.1) | 0.0 (0.0) | 0.6 (1.7) | 0.0 (0.1) | 2.8 (1.1) | 0.0 (0.3) | 1.4 (0.6) |
| Coastal Washington | 1.3 (0.7) | 0.0 (0.3) | 1.6 (0.6) | 7.8 (5.9) | 12.4 (2.4) | 12.3 (2.1) | 5.2 (4.7) | 11.5 (1.5) |
| Columbia River | 2.3 (0.9) | 1.4 (1.3) | 2.1 (0.7) | 4.4 (3.8) | 5.3 (1.6) | 3.0 (1.0) | 22.7 (8.2) | 4.8 (0.9) |
| Oregon | 0.0 (0.1) | 0.0 (0.2) | 0.0 (0.0) | 0.0 (0.7) | 0.0 (0.2) | 0.0 (0.0) | 0.0 (0.7) | 0.0 (0.1) |

Supplementary Table 4 concluded.

| Region/Conservation Unit | Barkley Sound and Alberni Inlet sport | | | | |
| --- | --- | --- | --- | --- | --- |
|  | June | July | August | September | Seasonal |
| Sample size | 10 | 11 | 78 | 113 | 213 |
| N-PBT | 6 | 6 | 66 | 105 | 184 |
| Southeast Alaska | 0.1 (2.3) | 0.0 (1.6) | 0.0 (0.2) | 0.0 (0.2) | 0.0 (0.1) |
| Alsek River | 0.0 (0.5) | 0.0 (0.8) | 0.0 (0.0) | 0.0 (0.0) | 0.0 (0.0) |
| Lower Stikine | 0.0 (0.4) | 0.0 (0.7) | 0.0 (0.1) | 0.0 (0.0) | 0.0 (0.0) |
| Lower Nass | 0.0 (1.1) | 0.4 (0.9) | 0.0 (0.1) | 0.0 (0.0) | 0.0 (0.0) |
| Upper Nass | 0.0 (0.9) | 0.1 (0.3) | 0.0 (0.1) | 0.0 (0.1) | 0.0 (0.1) |
| Portland Sound-Observatory Inlet-Portland Canal | 0.0 (0.5) | 0.0 (0.7) | 0.0 (0.1) | 0.0 (0.1) | 0.0 (0.0) |
| Skeena Estuary | 0.0 (1.0) | 0.0 (0.7) | 0.0 (0.1) | 0.0 (0.1) | 0.0 (0.0) |
| Lower Skeena | 0.0 (1.0) | 7.5 (7.7) | 0.0 (0.4) | 0.0 (0.1) | 0.0 (0.2) |
| Middle Skeena | 0.0 (1.6) | 0.0 (0.8) | 0.0 (0.3) | 0.0 (0.1) | 0.0 (0.1) |
| Upper Skeena | 0.0 (0.9) | 0.1 (1.4) | 0.0 (0.1) | 0.0 (0.1) | 0.0 (0.0) |
| Haida Gwaii-Graham Island Lowlands | 0.0 (1.0) | 0.0 (1.4) | 0.0 (0.2) | 0.0 (0.1) | 0.0 (0.1) |
| Haida Gwaii-East | 0.0 (1.3) | 0.0 (0.8) | 0.0 (0.2) | 0.0 (0.1) | 0.0 (0.1) |
| Haida Gwaii-West | 0.0 (1.3) | 0.0 (0.7) | 0.0 (0.2) | 0.0 (0.1) | 0.0 (0.0) |
| Northern Coastal Streams | 0.0 (2.0) | 0.4 (2.0) | 0.0 (0.4) | 0.0 (0.2) | 0.0 (0.2) |
| Hecate Strait Mainland | 0.0 (1.4) | 0.0 (1.9) | 0.0 (0.2) | 0.0 (0.1) | 0.0 (0.1) |
| Mussel-Kynoch | 0.0 (0.5) | 0.0 (0.4) | 0.0 (0.1) | 0.0 (0.0) | 0.0 (0.0) |
| Douglas Channel-Kitimat Arm | 0.0 (1.3) | 0.0 (1.3) | 0.0 (0.2) | 0.0 (0.1) | 0.0 (0.0) |
| Bella Coola-Dean Rivers | 0.0 (1.6) | 0.0 (1.3) | 0.0 (0.2) | 0.0 (0.2) | 0.0 (0.1) |
| Rivers Inlet | 0.0 (1.2) | 0.2 (1.9) | 0.0 (0.1) | 0.0 (0.1) | 0.0 (0.1) |
| Smith Inlet | 0.0 (0.6) | 0.0 (1.0) | 0.0 (0.1) | 0.0 (0.1) | 0.0 (0.1) |
| Southern Coastal Streams-Queen Charlotte Strait-Johnstone Strait-Southern Fjords | 0.0 (1.2) | 0.0 (1.9) | 0.0 (0.2) | 0.0 (0.1) | 0.0 (0.0) |
| Homathko-Klinaklini Rivers | 0.0 (1.1) | 0.0 (0.4) | 0.0 (0.0) | 0.0 (0.0) | 0.0 90.0) |
| Georgia Strait Mainland | 0.0 (1.6) | 0.0 (0.7) | 0.0 (0.1) | 0.0 (0.1) | 0.0 (0.0) |
| Howe Sound-Burrard Inlet | 11.7 (10.0) | 0.1 (2.0) | 0.0 (0.2) | 0.9 (0.9) | 0.9 (0.7) |
| East Vancouver Island-Georgia Strait | 0.1 (2.4) | 0.0 (1.4) | 0.1 (0.6) | 0.9 (0.9) | 0.5 (0.5) |
| East Vancouver Island-Johnstone Strait-Southern Fjords | 0.0 (0.6) | 0.0 (0.7) | 0.0 (0.5) | 0.0 (0.1) | 0.0 (0.1) |
| Nahwitti Lowland | 0.6 (3.1) | 0.1 (1.2) | 0.0 (0.3) | 0.0 (0.1) | 0.0 (0.1) |
| West Vancouver Island | 60.0 (16.0) | 54.7 (14.2) | 89.6 (3.6) | 95.6 (2.0) | 90.9 (2.1) |
| Clayoquot | 9.3 (10.5) | 0.0 (1.1) | 0.0 (0.1) | 0.8 (0.8) | 0.0 (0.1) |
| Juan de Fuca-Pachena | 0.0 (0.9) | 0.2 (1.4) | 2.6 (1.8) | 0.0 (0.2) | 1.2 (0.8) |
| Lower Fraser | 2.1 (7.9) | 0.0 (2.4) | 1.2 (1.3) | 1.8 (1.2) | 1.1 (0.8) |
| Lillooet | 7.9 (7.6) | 0.0 (1.1) | 0.0 (0.2) | 0.0 (0.1) | 0.3 (0.5) |
| Fraser Canyon | 0.0 (0.4) | 0.0 (0.3) | 0.0 (0.1) | 0.0 (0.1) | 0.0 (0.1) |
| Interior Fraser | 0.0 (1.2) | 0.0 (1.1) | 0.0 (0.1) | 0.0 (0.2) | 0.0 (0.1) |
| Lower Thompson | 0.0 (0.8) | 0.0 (0.3) | 0.0 (0.1) | 0.0 (0.1) | 0.0 (0.0) |
| North Thompson | 0.0 (2.1) | 0.0 (1.7) | 0.0 (0.3) | 0.0 (0.2) | 0.0 (0.1) |
| South Thompson | 0.0 (1.9) | 0.0 (1.9) | 0.0 (0.3) | 0.0 (0.3) | 0.0 (0.1) |
| Boundary Bay | 0.0 (0.9) | 0.1 (1.5) | 0.0 (0.2) | 0.0 (0.1) | 0.0 (0.1) |
| Nooksack River | 0.0 (1.2) | 0.3 (4.9) | 0.0 (0.2) | 0.0 (0.1) | 0.0 (0.0) |
| Skagit River | 7.5 (7.9) | 2.1 (3.0) | 0.5 (1.1) | 0.0 (0.1) | 0.2 (0.6) |
| Northern Puget Sound | 0.5 (2.5) | 15.3 (14.1) | 1.4 (1.9) | 0.0 (0.1) | 2.7 (1.3) |
| Mid-Puget Sound | 0.0 (1.8) | 7.0 (10.5) | 0.2 (0.7) | 0.0 (0.1) | 0.1 (0.3) |
| Southern Puget Sound | 0.0 (1.1) | 2.2 (6.2) | 0.0 (0.2) | 0.0 (0.1) | 0.0 (0.1) |
| Juan de Fuca Strait | 0.0 (0.3) | 0.1 (0.6) | 1.7 (1.6) | 0.0 (0.0) | 0.5 (0.5) |
| Hood Canal | 0.0 (0.6) | 0.0 (0.8) | 0.0 (0.1) | 0.0 (0.1) | 0.0 (0.1) |
| Coastal Washington | 0.0 (1.5) | 0.0 (1.8) | 1.3 (1.4) | 0.0 (0.1) | 0.5 (0.5) |
| Columbia River | 0.0 (1.7) | 9.1 (8.2) | 1.3 (1.3) | 0.0 (0.2) | 0.9 (0.7) |
| Oregon | 0.0 (1.6) | 0.0 (1.4) | 0.0 (0.2) | 0.0 (0.1) | 0.0 (0.2) |

Supplementary Table 5. Estimates of stock composition (%) by geographic region or CU for 2017 recreational fisheries in southern BC where direct sampling of creel surveys occurred and in which adipose fin clipped and unclipped individual coho salmon were retained (clipped + unclipped) compared with estimates derived from heads of clipped individuals (clipped) voluntarily sent to a central processing laboratory for potential CWT recovery. N-PBT is the number of individuals identified in the sample via PBT.

| Region/Conservation Unit | Johnstone Strait sport | | | | | | | Strait of Georgia sport | |
| --- | --- | --- | --- | --- | --- | --- | --- | --- | --- |
|  | June | | July | | August | | | June | |
|  | Clipped + unclipped | Clipped | Clipped + unclipped | Clipped | Clipped + unclipped | | Clipped | Clipped + unclipped | Clipped |
| Sample size | 49 | 11 | 204 | 105 | 62 | 144 | | 26 | 42 |
| % clipped | 29 | 100 | 15 | 100 | 18 | 100 | | 77 | 100 |
| N-PBT | 6 | 6 | 16 | 62 | 7 | 87 | | 10 | 30 |
| Southeast Alaska | 0.0 (0.5) | 0.0 (1.8) | 0.0 (0.1) | 0.0 (0.2) | 0.0 (0.3) | | 0.0 (0.1) | 0.0 (0.6) | 0.0 (0.4) |
| Alsek River | 0.0 (0.2) | 0.0 (0.4) | 0.0 (0.0) | 0.0 (0.0) | 0.0 (0.1) | | 0.0 (0.0) | 0.0 (0.1) | 0.0 (0.2) |
| Lower Stikine | 0.0 (0.1) | 0.0 (0.4) | 0.0 (0.0) | 0.0 (0.0) | 0.0 (0.1) | | 0.0 (0.0) | 0.0 (0.3) | 0.0 (0.1) |
| Lower Nass | 0.0 (0.2) | 0.0 (0.4) | 0.0 (0.1) | 0.0 (0.1) | 0.9 (1.5) | | 0.0 (0.3) | 0.0 (0.4) | 0.0 (0.2) |
| Upper Nass | 0.0 (0.1) | 0.0 (0.5) | 0.0 (0.0) | 0.0 (0.0) | 0.0 (0.1) | | 0.0 (0.0) | 0.0 (0.2) | 0.0 (0.1) |
| Portland Sound-Observatory Inlet-Portland Canal | 0.0 (0.2) | 0.0 (0.3) | 0.0 (0.0) | 0.0 (0.1) | 0.0 (0.1) | | 0.0 (0.1) | 0.0 (0.3) | 0.0 (0.2) |
| Skeena Estuary | 0.0 (0.2) | 0.0 (0.8) | 0.0 (0.1) | 0.0 (0.1) | 0.0 (0.2) | | 0.0 (0.1) | 0.0 (0.4) | 0.0 (0.2) |
| Lower Skeena | 0.0 (0.2) | 0.0 (0.8) | 0.0 (0.1) | 0.0 (0.1) | 0.0 (0.2) | | 0.0 (0.0) | 0.0 (0.5) | 0.0 (0.2) |
| Middle Skeena | 0.0 (0.2) | 0.0 (0.9) | 0.0 (0.1) | 0.0 (0.1) | 0.0 (0.2) | | 0.0 (0.1) | 0.0 (0.5) | 0.0 (0.1) |
| Upper Skeena | 0.0 (0.3) | 0.0 (0.7) | 0.0 (0.0) | 0.0 (0.1) | 0.0 (0.2) | | 0.0 (0.1) | 0.0 (0.4) | 0.0 (0.2) |
| Haida Gwaii-Graham Island Lowlands | 0.0 (0.4) | 0.0 (1.2) | 0.0 (0.1) | 0.0 (0.2) | 0.0 (0.3) | | 0.0 (0.1) | 0.0 (0.7) | 0.0 (0.4) |
| Haida Gwaii-East | 0.0 (0.2) | 0.0 (1.1) | 0.0 (0.1) | 0.0 (0.1) | 0.0 (0.2) | | 0.0 (0.1) | 0.0 (0.5) | 0.0 (0.3) |
| Haida Gwaii-West | 0.0 (0.3) | 0.0 (1.4) | 0.0 (0.1) | 0.0 (0.1) | 0.0 (0.2) | | 0.0 (0.1) | 0.0 (0.4) | 0.0 (0.3) |
| Northern Coastal Streams | 0.0 (0.6) | 0.0 (1.8) | 0.8 (1.2) | 0.0 (0.2) | 0.6 (1.9) | | 0.0 (0.2) | 0.0 (1.0) | 0.0 (0.6) |
| Hecate Strait Mainland | 0.0 (0.5) | 0.0 (0.8) | 1.0 (1.6) | 0.0 (0.2) | 0.0 (0.2) | | 0.0 (0.2) | 0.0 (0.7) | 0.0 (0.3) |
| Mussel-Kynoch | 0.0 (0.1) | 0.0 (0.3) | 0.0 (0.0) | 0.0 (0.0) | 0.0 (0.1) | | 0.0 (0.0) | 0.0 (0.2) | 0.0 (0.2) |
| Douglas Channel-Kitimat Arm | 0.0 (0.3) | 0.0 (1.6) | 1.7 (1.9) | 0.0 (0.1) | 2.3 (3.6) | | 0.1 (0.4) | 0.0 (0.5) | 0.0 (0.4) |
| Bella Coola-Dean Rivers | 2.5 (2.4) | 0.0 (1.1) | 6.4 (2.2) | 0.0 (0.2) | 5.6 (4.1) | | 1.0 (1.1) | 0.9 (2.6) | 0.0 (0.6) |
| Rivers Inlet | 0.0 (0.4) | 0.0 (0.6) | 1.1 (1.0) | 0.0 (0.1) | 0.0 (0.2) | | 0.0 (0.1) | 0.2 (1.2) | 0.0 (0.2) |
| Smith Inlet | 0.1 (0.3) | 0.0 (1.2) | 0.1 (0.4) | 0.0 (0.1) | 0.0 (0.2) | | 0.0 (0.1) | 0.0 (0.4) | 0.0 (0.2) |
| Southern Coastal Streams-Queen Charlotte Strait-Johnstone Strait-Southern Fjords | 17.5 (5.6) | 0.0 (1.2) | 19.0 (3.2) | 0.0 (0.2) | 27.5 (6.2) | | 0.0 (0.2) | 2.7 (3.5) | 0.0 (0.3) |
| Homathko-Klinaklini Rivers | 0.0 (0.2) | 0.0 (0.4) | 11.6 (2.7) | 0.0 (0.1) | 24.1 (5.8) | | 0.0 (0.2) | 0.0 (0.3) | 0.0 (0.1) |
| Georgia Strait Mainland | 0.0 (0.2) | 0.0 (0.9) | 0.0 (0.0) | 0.0 (0.0) | 0.0 (0.2) | | 0.0 (0.0) | 0.0 (0.4) | 0.0 (0.1) |
| Howe Sound-Burrard Inlet | 25.5 (6.7) | 0.0 (1.7) | 11.1 (3.0) | 13.7 (3.5) | 8.9 (3.6) | | 20.2 (3.5) | 25.5 (8.5) | 26.7 (6.6) |
| East Vancouver Island-Georgia Strait | 31.0 (7.7) | 27.7 (13.0) | 20.5 (3.3) | 33.3 (4.7) | 14.5 (4.6) | | 31.6 (4.0) | 10.4 (7.7) | 17.7 (6.2) |
| East Vancouver Island-Johnstone Strait-Southern Fjords | 0.1 (0.7) | 0.1 (1.2) | 0.0 (0.1) | 0.0 (0.5) | 0.0 (0.5) | | 0.0 (0.1) | 0.2 (1.2) | 0.0 (0.3) |
| Nahwitti Lowland | 3.5 (3.0) | 0.0 (1.5) | 7.3 (2.1) | 2.8 (1.7) | 1.6 (1.6) | | 1.6 (1.2) | 0.1 (1.0) | 0.0 (0.4) |
| West Vancouver Island | 0.0 (0.3) | 9.5 (9.5) | 4.7 (1.7) | 5.7 (2.2) | 1.6 (1.6) | | 6.3 (2.1) | 0.0 (0.5) | 0.0 (0.3) |
| Clayoquot | 3.5 (3.5) | 0.0 (0.9) | 0.5 (0.9) | 0.0 (0.1) | 0.0 (0.2) | | 0.0 (0.1) | 0.0 (0.4) | 0.0 (0.1) |
| Juan de Fuca-Pachena | 0.0 (0.1) | 0.0 (0.4) | 0.0 (0.2) | 0.0 (0.1) | 0.0 (0.2) | | 0.0 (0.1) | 0.0 (0.3) | 0.0 (0.2) |
| Lower Fraser | 4.6 (3.3) | 19.6 (11.4) | 1.6 (0.9) | 15.1 (3.5) | 3.2 (2.2) | | 16.0 (3.2) | 26.0 (9.0) | 27.2 (6.9) |
| Lillooet | 4.1 (2.7) | 0.0 (1.3) | 1.5 (0.8) | 0.0 (0.1) | 0.0 (0.2) | | 0.0 (0.1) | 0.0 (0.6) | 0.0 (0.4) |
| Fraser Canyon | 0.0 (0.2) | 0.0 (1.0) | 0.0 (0.0) | 0.0 (0.1) | 0.0 (0.2) | | 0.0 (0.1) | 0.0 (0.3) | 0.0 (0.2) |
| Interior Fraser | 0.0 (0.3) | 0.0 (0.7) | 0.0 (0.1) | 0.0 (0.1) | 0.0 (0.2) | | 0.0 (0.1) | 0.0 (0.5) | 0.0 (0.2) |
| Lower Thompson | 0.0 (0.3) | 0.0 (0.9) | 0.5 (0.5) | 0.0 (0.1) | 0.0 (0.1) | | 0.0 (0.1) | 0.0 (0.3) | 4.8 (3.1) |
| North Thompson | 0.0 (0.4) | 0.0 (2.3) | 0.0 (0.1) | 0.0 (0.3) | 0.0 (0.4) | | 0.0 (0.2) | 3.8 (3.6) | 0.0 (0.6) |
| South Thompson | 0.0 (0.5) | 0.0 (2.5) | 0.5 (0.5) | 0.0 (0.2) | 0.0 (0.3) | | 0.0 (0.2) | 0.1 (1.3) | 2.4 (2.4) |
| Boundary Bay | 0.0 (0.2) | 0.1 (0.8) | 0.5 (0.8) | 0.9 (1.0) | 0.0 (0.2) | | 0.0 (0.1) | 5.7 (3.1) | 2.7 (2.6) |
| Nooksack River | 0.1 (1.2) | 0.1 (2.9) | 0.1 (0.3) | 0.0 (0.1) | 2.5 (3.7) | | 0.1 (0.6) | 0.0 (0.5) | 0.6 (0.8) |
| Skagit River | 4.4 (4.2) | 4.1 (10.6) | 3.8 (2.1) | 5.6 (2.7) | 2.6 (3.6) | | 7.5 (2.7) | 9.9 (7.4) | 11.0 (5.2) |
| Northern Puget Sound | 0.6 (2.5) | 19.0 (14.1) | 0.1 (0.5) | 11.4 (3.7) | 0.0 (0.3) | | 9.7 (3.3) | 14.2 (9.5) | 0.0 (0.1) |
| Mid-Puget Sound | 0.1 (0.8) | 0.3 (4.1) | 2.2 (1.1) | 4.4 (2.4) | 2.3 (2.3) | | 3.3 (1.7) | 0.3 (1.2) | 0.0 (0.4) |
| Southern Puget Sound | 0.0 (0.3) | 1.2 (1.3) | 0.7 (0.8) | 0.0 (0.1) | 0.2 (0.8) | | 0.4 (1.0) | 0.0 (0.4) | 6.9 (4.4) |
| Juan de Fuca Strait | 0.0 (0.4) | 0.0 (0.7) | 0.0 (0.0) | 1.0 (1.0) | 0.0 (0.1) | | 0.0 (0.1) | 0.0 (0.3) | 0.0 (0.1) |
| Hood Canal | 0.2 (1.1) | 0.0 (0.5) | 0.0 (0.0) | 0.0 (0.1) | 0.0 (0.2) | | 0.0 (0.3) | 0.0 (0.3) | 0.0 (0.4) |
| Coastal Washington | 0.0 (0.3) | 0.0 (1.9) | 1.6 (0.9) | 4.9 (2.1) | 1.6 (1.5) | | 1.4 (1.0) | 0.0 (0.7) | 0.0 (0.4) |
| Columbia River | 2.0 (2.0) | 18.2 (10.1) | 0.0 (0.1) | 1.0 (1.0) | 0.0 (0.3) | | 0.7 (0.7) | 0.0 (0.7) | 0.0 (0.3) |
| Oregon | 0.0 (0.4) | 0.0 (1.8) | 1.0 (0.8) | 0.0 (0.2) | 0.0 (0.3) | | 0.0 (0.2) | 0.0 (0.8) | 0.0 (0.4) |

Supplementary Table 5 continued.

| Region/ Conservation Unit | Strait of Georgia sport | | | | | | | |
| --- | --- | --- | --- | --- | --- | --- | --- | --- |
|  | July | | August | | September | | October | |
|  | Clipped + unclipped | Clipped | Clipped + unclipped | Clipped | Clipped + unclipped | Clipped | Clipped + unclipped | Clipped |
| Sample size | 135 | 96 | 106 | 68 | 97 | 54 | 19 | 8 |
| % clipped | 88 | 100 | 52 | 100 | 49 | 100 | 68 | 100 |
| N-PBT | 79 | 57 | 30 | 48 | 38 | 48 | 11 | 8 |
| Southeast Alaska | 0.0 (0.2) | 0.0 (0.2) | 0.0 (0.2) | 0.0 (0.2) | 0.0 (0.1) | 0.0 (0.3) | 0.0 (1.2) | 0.0 (1.9) |
| Alsek River | 0.0 (0.0) | 0.0 (0.0) | 0.0 (0.1) | 0.0 (0.1) | 0.0 (0.0) | 0.0 (0.1) | 0.0 (0.4) | 0.0 (1.1) |
| Lower Stikine | 0.0 (0.0) | 0.0 (0.0) | 0.0 (0.1) | 0.0 (0.1) | 0.0 (0.0) | 0.0 (0.1) | 0.0 (0.4) | 0.0 (0.7) |
| Lower Nass | 0.0 (0.1) | 0.0 (0.1) | 0.0 (0.1) | 0.0 (0.1) | 0.0 (0.0) | 0.0 (0.2) | 0.0 (0.4) | 0.0 (0.6) |
| Upper Nass | 0.0 (0.0) | 0.0 (0.0) | 0.0 (0.0) | 0.0 (0.1) | 0.0 (0.1) | 0.0 (0.1) | 0.0 (0.4) | 0.0 (0.7) |
| Portland Sound-Observatory Inlet-Portland Canal | 0.0 (0.0) | 0.0 (0.0) | 0.0 (0.1) | 0.0 (0.1) | 0.0 (0.0) | 0.0 (0.1) | 0.0 (0.3) | 0.0 (0.6) |
| Skeena Estuary | 0.0 (0.1) | 0.0 (0.1) | 0.0 (0.1) | 0.0 (0.1) | 0.0 (0.1) | 0.0 (0.1) | 0.0 (0.6) | 0.0 (1.5) |
| Lower Skeena | 0.0 (0.1) | 0.0 (0.1) | 0.0 (0.1) | 0.0 (0.2) | 0.0 (0.1) | 0.0 (0.2) | 0.0 (0.8) | 0.0 (1.6) |
| Middle Skeena | 0.0 (0.1) | 0.0 (0.1) | 0.0 (0.1) | 0.0 (0.2) | 0.0 (0.1) | 0.0 (0.2) | 0.0 (0.5) | 0.0 (1.2) |
| Upper Skeena | 0.0 (0.1) | 0.0 (0.1) | 0.0 (0.1) | 0.0 (0.2) | 0.0 (0.1) | 0.0 (0.1) | 0.0 (0.5) | 0.0 (1.2) |
| Haida Gwaii-Graham Island Lowlands | 0.0 (0.1) | 0.0 (0.2) | 0.0 (0.2) | 0.0 (0.3) | 0.0 (0.1) | 0.0 (0.5) | 0.0 (1.0) | 0.0 (2.5) |
| Haida Gwaii-East | 0.0 (0.1) | 0.0 (0.1) | 0.0 (0.1) | 0.0 (0.1) | 0.0 (0.1) | 0.0 (0.2) | 0.0 (0.6) | 0.0 (1.1) |
| Haida Gwaii-West | 0.0 (0.1) | 0.0 (0.1) | 0.0 (0.1) | 0.0 (0.2) | 0.0 (0.1) | 0.0 (0.2) | 0.0 (0.8) | 0.0 (1.2) |
| Northern Coastal Streams | 0.0 (0.2) | 0.0 (0.3) | 0.0 (0.2) | 0.0 (0.3) | 0.0 (0.2) | 0.0 (0.4) | 0.0 (1.2) | 0.0 (2.7) |
| Hecate Strait Mainland | 0.0 (0.1) | 0.0 (0.1) | 0.0 (0.1) | 0.0 (0.2) | 0.0 (0.1) | 0.0 (0.3) | 0.0 (0.7) | 0.0 (1.4) |
| Mussel-Kynoch | 0.0 (0.0) | 0.0 (0.0) | 0.0 (0.0) | 0.0 (0.1) | 0.0 (0.1) | 0.0 (0.1) | 0.0 (0.3) | 0.0 (0.6) |
| Douglas Channel-Kitimat Arm | 0.6 (0.7) | 0.0 (0.1) | 0.0 (0.1) | 0.0 (0.3) | 0.0 (0.1) | 0.0 (0.1) | 0.0 (0.6) | 0.0 (1.6) |
| Bella Coola-Dean Rivers | 0.1 (0.4) | 0.0 (0.2) | 1.6 (1.4) | 0.0 (0.3) | 0.1 (0.4) | 0.0 (0.4) | 0.0 (0.8) | 0.0 (1.8) |
| Rivers Inlet | 0.0 (0.1) | 0.0 (0.1) | 0.0 (0.1) | 0.0 (0.2) | 0.1 (0.5) | 0.0 (0.4) | 0.0 (0.6) | 0.0 (1.7) |
| Smith Inlet | 0.0 (0.1) | 0.0 (0.2) | 0.0 (0.1) | 0.0 (0.1) | 0.0 (0.1) | 0.0 (0.2) | 0.0 (0.5) | 0.0 (1.4) |
| Southern Coastal Streams-Queen Charlotte Strait-Johnstone Strait-Southern Fjords | 0.0 (0.1) | 0.0 (0.4) | 0.0 (0.2) | 0.0 (0.2) | 0.4 (0.9) | 0.0 (0.3) | 0.0 (0.7) | 0.0 (1.5) |
| Homathko-Klinaklini Rivers | 0.0 (0.0) | 0.0 (0.0) | 17.1 (4.0) | 0.0 (0.0) | 0.4 (1.0) | 0.0 (0.0) | 0.0 (0.2) | 0.0 (0.8) |
| Georgia Strait Mainland | 0.0 (0.1) | 0.0 (0.1) | 0.0 (0.2) | 0.0 (0.1) | 0.0 (0.1) | 0.0 (0.2) | 0.0 (0.4) | 0.0 (0.6) |
| Howe Sound-Burrard Inlet | 21.6 (3.7) | 28.3 (4.7) | 25.2 (4.9) | 34.0 (5.8) | 33.6 (4.8) | 55.2 (6.8) | 39.1 (10.8) | 50.0 (16.2) |
| East Vancouver Island-Georgia Strait | 21.0 (3.8) | 9.3 (3.1) | 12.1 (3.5) | 16.6 (4.7) | 16.3 (4.3) | 11.2 (4.2) | 22.3 (9.3) | 12.5 (10.0) |
| East Vancouver Island-Johnstone Strait-Southern Fjords | 0.7 (1.1) | 0.0 (0.2) | 0.1 (0.5) | 0.0 (0.1) | 1.4 (1.7) | 0.0 (0.1) | 1.1 (5.6) | 0.0 (0.5) |
| Nahwitti Lowland | 0.0 (0.1) | 0.0 (0.2) | 0.0 (0.2) | 0.1 (0.4) | 0.0 (0.1) | 0.0 (0.3) | 0.0 (0.9) | 0.0 (1.7) |
| West Vancouver Island | 0.0 (0.1) | 0.0 (0.2) | 0.0 (0.1) | 0.0 (0.2) | 0.0 (0.1) | 1.9 (1.8) | 0.0 (0.7) | 0.0 (0.7) |
| Clayoquot | 0.0 (0.1) | 0.0 (0.1) | 0.0 (0.1) | 0.0 (0.5) | 0.0 (0.1) | 0.0 (0.2) | 0.0 (0.7) | 0.0 (0.5) |
| Juan de Fuca-Pachena | 0.0 (0.1) | 0.0 (0.1) | 0.0 (0.1) | 0.0 (0.1) | 0.0 (0.1) | 0.0 (0.1) | 0.0 (0.4) | 0.0 (0.7) |
| Lower Fraser | 39.7 (4.4) | 36.9 (4.9) | 24.3 (4.4) | 34.1 (5.7) | 32.4 (4.8) | 24.8 (5.5) | 24.1 (9.9) | 37.5 (15.3) |
| Lillooet | 0.0 (0.1) | 0.0 (0.1) | 1.0 (1.0) | 0.0 (0.2) | 2.1 (1.4) | 0.0 (0.2) | 5.6 (5.1) | 0.0 (1.7) |
| Fraser Canyon | 0.0 (0.1) | 0.0 (0.1) | 0.0 (0.0) | 0.0 (0.1) | 0.5 (1.1) | 0.0 (0.2) | 0.0 (0.5) | 0.0 (0.8) |
| Interior Fraser | 0.0 (0.1) | 0.0 (0.1) | 0.0 (0.1) | 0.0 (0.2) | 1.9 (1.6) | 0.0 (0.2) | 0.0 (0.6) | 0.0 (1.2) |
| Lower Thompson | 0.0 (0.1) | 0.0 (0.1) | 0.0 (0.1) | 0.0 (0.1) | 1.2 (1.1) | 0.0 (0.1) | 0.0 (0.5) | 0.0 (1.0) |
| North Thompson | 0.0 (0.2) | 0.0 (0.3) | 2.0 (1.4) | 0.0 (0.3) | 1.7 (1.6) | 0.0 (0.5) | 0.0 (1.3) | 0.0 (2.7) |
| South Thompson | 0.0 (0.2) | 0.0 (0.3) | 0.0 (0.4) | 0.0 (0.3) | 0.0 (0.2) | 0.0 (0.4) | 0.0 (1.3) | 0.0 (2.1) |
| Boundary Bay | 0.8 (0.8) | 1.7 (1.3) | 0.0 (0.3) | 0.1 (0.5) | 2.4 (1.7) | 1.9 (1.7) | 0.0 (0.6) | 0.0 (1.1) |
| Nooksack River | 0.0 (0.5) | 1.1 (1.9) | 0.3 (0.8) | 0.0 (0.1) | 0.0 (0.2) | 0.0 (0.2) | 6.2 (11.0) | 0.0 (1.3) |
| Skagit River | 10.8 (3.2) | 8.8 (3.3) | 8.2 (3.9) | 6.2 (3.5) | 3.6 (2.2) | 0.0 (0.6) | 1.3 (5.4) | 0.0 (0.7) |
| Northern Puget Sound | 0.8 (1.6) | 5.0 (3.4) | 0.7 (1.7) | 8.9 (3.7) | 0.1 (0.4) | 4.8 (3.8) | 0.2 (2.1) | 0.0 (1.1) |
| Mid-Puget Sound | 0.0 (0.2) | 3.0 (3.1) | 0.1 (0.4) | 0.0 (0.3) | 0.0 (0.3) | 0.3 (1.9) | 0.2 (2.4) | 0.0 (0.9) |
| Southern Puget Sound | 3.8 (2.0) | 5.8 (2.6) | 5.7 (2.7) | 0.0 (0.1) | 1.8 (2.1) | 0.0 (0.4) | 0.0 (0.5) | 0.0 (1.1) |
| Juan de Fuca Strait | 0.0 (0.1) | 0.0 (0.3) | 0.0 (0.1) | 0.0 (0.1) | 0.0 (0.1) | 0.0 (0.1) | 0.0 (0.5) | 0.0 (0.7) |
| Hood Canal | 0.0 (0.1) | 0.0 (0.1) | 0.0 (0.1) | 0.0 (0.2) | 0.0 (0.1) | 0.0 (0.1) | 0.0 (0.6) | 0.0 (0.9) |
| Coastal Washington | 0.0 (0.1) | 0.0 (0.2) | 1.5 (1.4) | 0.0 (0.2) | 0.0 (0.2) | 0.0 (0.4) | 0.0 (0.8) | 0.0 (1.3) |
| Columbia River | 0.0 (0.1) | 0.0 (0.2) | 0.0 (0.2) | 0.0 (0.2) | 0.0 (0.2) | 0.0 (0.3) | 0.0 (0.9) | 0.0 (1.6) |
| Oregon | 0.0 (0.1) | 0.0 (0.2) | 0.0 (0.2) | 0.0 (0.3) | 0.0 (0.2) | 0.0 (0.3) | 0.0 (0.9) | 0.0 (1.9) |

Supplementary Table 5 continued.

| Region/Conservation Unit | Juan de Fuca Strait sport | | | | | | | |
| --- | --- | --- | --- | --- | --- | --- | --- | --- |
|  | June | | August | | September | | October | |
|  | Clipped + unclipped | Clipped | Clipped + unclipped | Clipped | Clipped + unclipped | Clipped | Clipped + unclipped | Clipped |
| Sample size | 11 | 15 | 15 | 59 | 70 | 263 | 73 | 73 |
| % clipped | 100 | 100 | 93 | 100 | 89 | 100 | 37 | 100 |
| N-PBT | 1 | 6 | 3 | 20 | 15 | 91 | 24 | 53 |
| Southeast Alaska | 0.0 (1.6) | 0.0 (1.1) | 0.0 (1.2) | 0.0 (0.3) | 0.0 (0.3) | 0.0 (0.1) | 0.0 (0.2) | 0.0 (0.2) |
| Alsek River | 0.0 (0.5) | 0.0 (0.2) | 0.0 (0.4) | 0.0 (0.2) | 0.0 (0.1) | 0.0 (0.0) | 0.0 (0.1) | 0.0 (0.1) |
| Lower Stikine | 0.0 (0.5) | 0.0 (0.6) | 0.0 (0.4) | 0.0 (0.1) | 0.0 (0.1) | 0.0 (0.0) | 0.0 (0.1) | 0.0 (0.1) |
| Lower Nass | 0.0 (0.6) | 0.0 (1.0) | 0.0 (0.4) | 0.0 (0.1) | 0.0 (0.1) | 0.0 (0.0) | 0.0 (0.1) | 0.0 (0.1) |
| Upper Nass | 0.0 (0.4) | 0.0 (0.3) | 0.0 (0.5) | 0.0 (0.1) | 0.0 (0.1) | 0.0 (0.0) | 0.0 (0.1) | 0.0 (0.0) |
| Portland Sound-Observatory Inlet-Portland Canal | 0.0 (0.5) | 0.0 (0.3) | 0.0 (0.3) | 0.0 (0.1) | 0.0 (0.1) | 0.0 (0.0) | 0.0 (0.1) | 0.0 (0.1) |
| Skeena Estuary | 0.0 (0.8) | 0.0 (0.4) | 0.0 (0.7) | 0.0 (0.2) | 0.0 (0.2) | 0.0 (0.0) | 0.0 (0.1) | 0.0 (0.1) |
| Lower Skeena | 0.0 (1.0) | 0.0 (0.5) | 0.0 (0.7) | 0.0 (0.1) | 0.0 (0.1) | 0.0 (0.1) | 0.0 (0.2) | 0.0 (0.1) |
| Middle Skeena | 0.0 (1.0) | 0.0 (0.6) | 0.0 (0.9) | 0.0 (0.1) | 0.0 (0.2) | 0.0 (0.0) | 0.0 (0.2) | 0.0 (0.1) |
| Upper Skeena | 0.0 (0.9) | 0.0 (0.8) | 0.0 (0.7) | 0.0 (0.2) | 0.0 (0.1) | 0.0 (0.0) | 0.0 (0.1) | 0.0 (0.2) |
| Haida Gwaii-Graham Island Lowlands | 0.0 (1.4) | 0.0 (0.8) | 0.0 (1.1) | 0.0 (0.2) | 0.0 (0.3) | 0.0 (0.1) | 0.0 (0.2) | 0.0 (0.3) |
| Haida Gwaii-East | 0.0 (1.1) | 0.0 (0.7) | 0.0 (0.7) | 0.0 (0.2) | 0.0 (0.2) | 0.0 (0.0) | 0.0 (0.2) | 0.0 (0.2) |
| Haida Gwaii-West | 0.0 (1.3) | 0.0 (0.7) | 0.0 (0.8) | 0.0 (0.1) | 0.0 (0.2) | 0.0 (0.0) | 0.0 (0.2) | 0.0 (0.2) |
| Northern Coastal Streams | 0.0 (2.1) | 0.0 (1.6) | 0.0 (1.7) | 0.0 (0.4) | 0.1 (0.5) | 0.0 (0.1) | 0.0 (0.4) | 0.0 (0.3) |
| Hecate Strait Mainland | 0.0 (1.3) | 0.0 (0.8) | 0.0 (0.9) | 0.0 (0.3) | 0.0 (0.3) | 0.0 (0.1) | 0.0 (0.2) | 0.0 (0.2) |
| Mussel-Kynoch | 0.0 (0.4) | 0.0 (0.4) | 0.0 (0.4) | 0.0 (0.1) | 0.0 (0.1) | 0.0 (0.0) | 0.0 (0.1) | 0.0 (0.2) |
| Douglas Channel-Kitimat Arm | 0.0 (0.9) | 0.0 (0.6) | 0.0 (1.0) | 0.0 (0.1) | 0.0 (0.3) | 0.0 (0.1) | 0.0 (0.2) | 0.0 (0.2) |
| Bella Coola-Dean Rivers | 0.0 (1.3) | 0.0 (1.3) | 0.0 (1.2) | 0.0 (0.3) | 0.0 (0.3) | 0.0 (0.0) | 0.0 (0.3) | 0.0 (0.2) |
| Rivers Inlet | 0.0 (1.0) | 0.0 (1.2) | 0.0 (0.7) | 0.0 (0.2) | 0.0 (0.2) | 0.0 (0.1) | 0.0 (0.2) | 0.0 (0.2) |
| Smith Inlet | 0.0 (1.0) | 0.0 (0.9) | 0.0 (0.7) | 0.0 (0.2) | 0.0 (0.2) | 0.0 (0.1) | 0.0 (0.2) | 0.0 (0.1) |
| Southern Coastal Streams-Queen Charlotte Strait-Johnstone Strait-Southern Fjords | 0.0 (1.0) | 0.0 (0.8) | 0.0 (0.9) | 0.0 (0.3) | 1.3 (1.5) | 0.0 (0.1) | 0.0 (0.2) | 0.0 (0.2) |
| Homathko-Klinaklini Rivers | 0.0 (0.6) | 0.0 (0.4) | 0.0 (0.3) | 0.0 (0.1) | 0.0 (0.1) | 0.0 (0.1) | 0.0 (0.1) | 0.0 (0.1) |
| Georgia Strait Mainland | 0.0 (0.7) | 0.0 (0.6) | 0.0 (0.4) | 0.0 (0.1) | 0.0 (0.1) | 0.0 (0.0) | 0.0 (0.1) | 0.0 (0.1) |
| Howe Sound-Burrard Inlet | 10.4 (8.8) | 13.3 (8.3) | 18.6 (10.2) | 18.2 (4.9) | 1.5 (1.5) | 6.8 (1.6) | 9.7 (3.6) | 11.0 (3.6) |
| East Vancouver Island-Georgia Strait | 0.3 (3.0) | 7.8 (6.6) | 3.2 (6.8) | 7.6 (3.8) | 5.0 (2.7) | 5.9 (1.5) | 32.9 (5.5) | 10.7 (3.8) |
| East Vancouver Island-Johnstone Strait-Southern Fjords | 0.0 (0.8) | 0.0 (0.3) | 0.0 (0.6) | 0.0 (0.1) | 0.1 (0.5) | 0.0 (0.0) | 0.0 (0.3) | 0.0 (0.2) |
| Nahwitti Lowland | 0.0 (1.3) | 0.0 (1.1) | 0.0 (1.2) | 0.0 (0.4) | 0.0 (0.3) | 0.0 (0.1) | 0.0 (0.2) | 0.0 (0.2) |
| West Vancouver Island | 0.0 (1.0) | 0.0 (0.9) | 0.0 (0.8) | 1.7 (1.7) | 1.4 (1.4) | 0.0 (0.1) | 0.0 (0.2) | 0.0 (0.1) |
| Clayoquot | 0.0 (0.8) | 0.0 (0.7) | 0.1 (1.4) | 0.0 (0.2) | 0.0 (0.2) | 0.0 (0.0) | 0.0 (0.1) | 0.0 (0.2) |
| Juan de Fuca-Pachena | 0.0 (1.1) | 0.0 (0.6) | 0.0 (0.5) | 3.4 (2.3) | 0.0 (0.1) | 0.8 (0.5) | 1.5 (2.0) | 0.0 (0.1) |
| Lower Fraser | 9.1 (8.0) | 13.7 (8.5) | 6.7 (6.1) | 6.8 (3.3) | 16.8 (4.5) | 19.6 (2.4) | 37.5 (5.7) | 67.5 (5.5) |
| Lillooet | 0.0 (1.0) | 0.0 (0.8) | 0.0 (1.1) | 0.0 (0.2) | 0.0 (0.2) | 0.0 (0.1) | 5.3 (2.6) | 0.0 (0.2) |
| Fraser Canyon | 0.0 (0.8) | 0.0 (0.5) | 0.0 (0.5) | 0.0 (0.1) | 0.0 (0.1) | 0.0 (0.0) | 0.0 (0.1) | 0.0 (0.2) |
| Interior Fraser | 0.0 (1.0) | 0.0 (0.6) | 0.0 (0.9) | 0.0 (0.2) | 0.0 (0.2) | 0.0 (0.0) | 0.0 (0.2) | 0.0 (0.2) |
| Lower Thompson | 0.0 (0.7) | 0.0 (0.4) | 0.0 (0.5) | 0.0 (0.1) | 0.0 (0.1) | 1.9 (0.8) | 0.0 (0.1) | 0.0 (0.1) |
| North Thompson | 0.0 (2.0) | 0.0 (1.4) | 0.0 (1.5) | 0.0 (0.4) | 0.0 (0.4) | 0.0 (0.1) | 0.0 (0.4) | 0.0 (0.2) |
| South Thompson | 0.0 (2.0) | 0.0 (1.4) | 0.0 (1.3) | 3.4 (2.4) | 1.4 (1.5) | 0.8 (0.6) | 0.0 (0.3) | 0.0 (0.4) |
| Boundary Bay | 0.0 (0.8) | 6.7 (4.6) | 0.0 (0.6) | 1.7 (1.0) | 1.4 (1.4) | 3.4 (1.1) | 1.4 (1.4) | 1.4 (1.3) |
| Nooksack River | 3.7 (8.7) | 1.1 (5.0) | 0.2 (2.0) | 0.0 (0.1) | 0.1 (0.6) | 1.2 (1.8) | 0.0 (0.2) | 0.5 (1.1) |
| Skagit River | 16.3 (14.6) | 0.2 (1.5) | 0.4 (1.9) | 11.6 (5.0) | 6.4 (3.9) | 5.3 (1.7) | 4.1 (2.7) | 0.2 (1.0) |
| Northern Puget Sound | 9.0 (15.4) | 0.4 (1.6) | 35.8 (17.5) | 23.2 (6.3) | 24.0 (6.0) | 23.0 (3.3) | 3.8 (2.6) | 0.5 (1.1) |
| Mid-Puget Sound | 32.4 (14.0) | 41.2 (14.2) | 24.1 (13.7) | 15.7 (5.7) | 18.5 (5.3) | 12.7 (2.6) | 0.0 (0.2) | 0.0 (0.1) |
| Southern Puget Sound | 7.0 (8.1) | 14.7 (9.2) | 4.1 (8.8) | 0.1 (0.3) | 13.3 (4.5) | 13.2 (2.7) | 3.7 (2.3) | 0.0 (0.5) |
| Juan de Fuca Strait | 11.4 (9.5) | 0.4 (1.2) | 0.0 (0.6) | 0.5 (1.3) | 5.0 (2.7) | 1.8 (0.8) | 0.0 (0.1) | 6.9 (3.0) |
| Hood Canal | 0.0 (0.8) | 0.2 (3.0) | 0.0 (0.5) | 0.0 (0.2) | 0.2 (0.8) | 0.0 (0.1) | 0.0 (0.1) | 0.0 (0.1) |
| Coastal Washington | 0.3 (1.9) | 0.0 (1.3) | 0.1 (1.4) | 4.4 (2.7) | 0.6 (1.3) | 1.3 (0.7) | 0.0 (0.3) | 0.0 (0.3) |
| Columbia River | 0.0 (1.4) | 0.0 (1.4) | 6.7 (5.9) | 1.7 (1.7) | 2.9 (2.0) | 2.3 (0.9) | 0.0 (0.3) | 1.4 (1.3) |
| Oregon | 0.0 (1.5) | 0.0 (1.1) | 0.0 (1.2) | 0.0 (0.3) | 0.0 (0.3) | 0.0 (0.1) | 0.0 (0.3) | 0.0 (0.2) |

Supplementary Table 5 concluded.

| Region/Conservation Unit | West coast Vancouver Island sport | | | | | | | |
| --- | --- | --- | --- | --- | --- | --- | --- | --- |
|  | June | | July | | August | | September | |
|  | Clipped + unclipped | Clipped | Clipped+ unclipped | Clipped | Clipped + unclipped | Clipped | Clipped + unclipped | Clipped |
| Sample size | 118 | 23 | 253 | 205 | 181 | 267 | 24 | 22 |
| % clipped | 14 | 100 | 17 | 100 | 18 | 100 | 8 | 100 |
| N-PBT | 1 | 2 | 7 | 33 | 8 | 56 | 0 | 8 |
| Southeast Alaska | 0.9 (0.9) | 0.0 (0.6) | 2.4 (1.1) | 0.0 (0.1) | 0.6 (0.9) | 0.0 (0.1) | 0.0 (1.0) | 0.0 (0.9) |
| Alsek River | 0.0 (0.0) | 0.0 (0.5) | 0.0 (0.0) | 0.0 (0.0) | 0.0 (0.0) | 0.0 (0.0) | 0.0 (0.2) | 0.0 (0.1) |
| Lower Stikine | 0.0 (0.1) | 0.0 (0.0) | 0.0 (0.1) | 0.0 (0.0) | 0.0 (0.0) | 0.0 (0.0) | 0.0 (0.3) | 0.0 (0.1) |
| Lower Nass | 0.0 (0.1) | 0.0 (0.4) | 0.0 (0.0) | 0.0 (0.0) | 0.0 (0.1) | 0.0 (0.0) | 0.0 (0.4) | 0.0 (0.2) |
| Upper Nass | 0.0 (0.0) | 0.0 (0.2) | 0.0 (0.0) | 0.0 (0.0) | 0.0 (0.0) | 0.0 (0.0) | 0.0 (0.2) | 0.0 (0.1) |
| Portland Sound-Observatory Inlet-Portland Canal | 0.0 (0.1) | 0.0 (0.6) | 0.0 (0.0) | 0.0 (0.0) | 0.0 (0.0) | 0.0 (0.0) | 0.0 (0.2) | 0.0 (0.2) |
| Skeena Estuary | 0.0 (0.1) | 0.0 (0.6) | 0.0 (0.1) | 0.0 (0.0) | 0.0 (0.1) | 0.0 (0.0) | 0.0 (0.5) | 0.0 (0.6) |
| Lower Skeena | 0.0 (0.1) | 0.1 (0.8) | 0.0 (0.1) | 0.0 (0.1) | 0.0 (0.1) | 0.0 (0.0) | 0.0 (0.5) | 0.0 (0.7) |
| Middle Skeena | 0.0 (0.1) | 0.1 (0.4) | 0.0 (0.1) | 0.0 (0.0) | 0.0 (0.1) | 0.0 (0.0) | 0.0 (0.5) | 0.0 (0.7) |
| Upper Skeena | 0.0 (0.1) | 0.0 (0.4) | 0.0 (0.0) | 0.0 (0.1) | 0.0 (0.1) | 0.0 (0.1) | 0.0 (0.5) | 0.0 (0.3) |
| Haida Gwaii-Graham Island Lowlands | 0.0 (0.1) | 0.0 (0.8) | 0.0 (0.1) | 0.0 (0.1) | 0.0 (0.1) | 0.0 (0.1) | 0.0 (0.8) | 0.0 (0.7) |
| Haida Gwaii-East | 0.0 (0.1) | 0.0 (0.6) | 0.0 (0.1) | 0.0 (0.1) | 0.0 (0.1) | 0.0 (0.0) | 0.0 (0.5) | 0.0 (0.5) |
| Haida Gwaii-West | 0.0 (0.1) | 0.0 (0.4) | 0.0 (0.1) | 0.0 (0.1) | 0.0 (0.1) | 0.0 (0.0) | 0.0 (0.4) | 0.0 (0.7) |
| Northern Coastal Streams | 4.9 (2.8) | 0.2 (1.5) | 0.3 (0.7) | 0.1 (0.3) | 3.9 (2.1) | 0.0 (0.1) | 0.0 (0.9) | 0.0 (1.2) |
| Hecate Strait Mainland | 0.4 (1.8) | 0.0 (0.8) | 3.5 (1.3) | 0.0 (0.1) | 3.6 (2.0) | 0.0 (0.1) | 0.0 (0.7) | 0.0 (0.8) |
| Mussel-Kynoch | 0.0 (0.1) | 0.0 (0.2) | 0.0 (0.0) | 0.0 (0.0) | 0.0 (0.0) | 0.0 (0.0) | 0.0 (0.2) | 0.0 (0.2) |
| Douglas Channel-Kitimat Arm | 0.0 (0.2) | 0.0 (0.6) | 0.0 (0.1) | 0.0 (0.1) | 0.1 (0.5) | 0.0 (0.1) | 0.0 (0.6) | 0.0 (0.8) |
| Bella Coola-Dean Rivers | 0.0 (0.2) | 0.0 (0.9) | 3.2 (1.4) | 0.5 (0.5) | 1.2 (1.3) | 0.0 (0.0) | 0.0 (0.7) | 0.0 (0.4) |
| Rivers Inlet | 2.4 (1.8) | 0.0 (0.9) | 2.7 (1.2) | 0.0 (0.2) | 0.0 (0.3) | 0.0 (0.0) | 0.0 (0.9) | 0.0 (0.6) |
| Smith Inlet | 0.0 (0.2) | 0.0 (0.6) | 1.3 (0.8) | 0.0 (0.1) | 0.0 (0.1) | 0.0 (0.0) | 0.1 (1.1) | 0.0 (0.3) |
| Southern Coastal Streams-Queen Charlotte Strait-Johnstone Strait-Southern Fjords | 6.0 (2.8) | 0.0 (0.6) | 6.5 (1.8) | 0.6 (0.7) | 4.1 (1.8) | 0.0 (0.1) | 0.1 (1.0) | 0.0 (0.5) |
| Homathko-Klinaklini Rivers | 0.1 (0.4) | 0.0 (0.8) | 4.4 (1.5) | 0.9 (0.7) | 0.0 (0.3) | 0.0 (0.0) | 0.0 (0.4) | 0.0 (0.2) |
| Georgia Strait Mainland | 0.0 (0.1) | 0.0 (0.2) | 0.0 (0.0) | 0.0 (0.0) | 0.0 (0.0) | 0.0 (0.0) | 0.0 (0.3) | 0.0 (0.5) |
| Howe Sound-Burrard Inlet | 4.6 (2.1) | 0.1 (1.7) | 5.2 (1.6) | 3.0 (1.3) | 0.9 (0.9) | 2.1 (0.7) | 0.1 (1.2) | 0.0 (1.2) |
| East Vancouver Island-Georgia Strait | 17.5 (4.4) | 1.5 (2.8) | 12.9 (2.7) | 10.5 (2.5) | 8.9 (2.7) | 4.6 (1.6) | 0.1 (1.2) | 11.4 (8.3) |
| East Vancouver Island-Johnstone Strait-Southern Fjords | 0.0 (0.2) | 0.0 (0.5) | 0.0 (0.0) | 0.0 (0.1) | 0.0 (0.1) | 0.0 (0.0) | 0.0 (0.4) | 0.0 (0.6) |
| Nahwitti Lowland | 34.6 (5.2) | 13.4 (7.0) | 17.1 (2.9) | 4.0 (1.7) | 14.5 (3.3) | 2.8 (1.1) | 5.8 (5.8) | 0.0 (0.9) |
| West Vancouver Island | 13.8 (3.9) | 4.5 (4.2) | 11.8 (2.3) | 6.7 (1.7) | 27.9 (3.6) | 10.4 (1.9) | 81.2 (8.4) | 17.9 (8.1) |
| Clayoquot | 2.2 (2.8) | 0.0 (1.2) | 7.4 (1.9) | 0.8 (1.3) | 11.8 (3.1) | 0.0 (0.0) | 0.5 (2.3) | 0.0 (0.3) |
| Juan de Fuca-Pachena | 0.3 (1.2) | 0.0 (0.3) | 2.6 (1.3) | 2.0 (1.1) | 0.8 (1.2) | 4.2 (1.3) | 0.0 (0.6) | 4.5 (3.4) |
| Lower Fraser | 0.4 (1.1) | 10.0 (6.5) | 1.6 (1.4) | 5.9 (1.6) | 1.0 (0.8) | 6.6 (1.5) | 0.0 (1.3) | 9.1 (6.0) |
| Lillooet | 2.2 (1.5) | 0.0 (0.7) | 2.6 (1.4) | 0.0 (0.1) | 1.6 (1.0) | 0.0 (0.1) | 0.0 (0.5) | 0.0 (0.4) |
| Fraser Canyon | 0.0 (0.1) | 0.0 (0.5) | 0.3 (0.4) | 0.0 (0.0) | 0.0 (0.1) | 0.0 (0.0) | 0.0 (0.4) | 0.0 (0.7) |
| Interior Fraser | 0.0 (0.1) | 0.0 (0.6) | 0.0 (0.0) | 0.0 (0.1) | 0.0 (0.1) | 0.0 (0.0) | 0.0 (0.4) | 0.0 (0.5) |
| Lower Thompson | 0.0 (0.1) | 0.0 (0.4) | 0.0 (0.0) | 0.0 (0.0) | 0.0 (0.0) | 0.4 (0.4) | 0.0 (0.3) | 0.0 (0.4) |
| North Thompson | 0.0 (0.2) | 0.0 (1.0) | 0.5 (0.5) | 0.0 (0.1) | 0.6 (0.6) | 0.0 (0.1) | 0.0 (1.0) | 0.0 (1.1) |
| South Thompson | 0.0 (0.2) | 0.0 (1.1) | 0.0 (0.1) | 0.0 (0.1) | 0.0 (0.1) | 0.0 (0.1) | 0.0 (1.0) | 0.0 (1.0) |
| Boundary Bay | 0.0 (0.1) | 0.0 (0.5) | 0.0 (0.1) | 0.5 (0.5) | 0.0 (0.1) | 0.4 (0.0) | 0.0 (0.4) | 0.0 (0.6) |
| Nooksack River | 0.1 (0.7) | 5.0 (9.4) | 0.0 (0.1) | 0.1 (0.1) | 0.2 (1.1) | 0.4 (1.0) | 0.1 (0.7) | 0.0 (0.5) |
| Skagit River | 0.0 (0.1) | 4.2 (6.1) | 0.0 (0.1) | 5.8 (1.9) | 0.1 (0.6) | 6.5 (2.0) | 0.1 (1.3) | 0.1 (0.7) |
| Northern Puget Sound | 0.0 (0.2) | 38.3 (11.9) | 3.4 (1.6) | 10.4 (3.1) | 6.3 (2.2) | 9.5 (2.5) | 9.9 (8.1) | 18.0 (7.7) |
| Mid-Puget Sound | 4.2 (2.1) | 0.0 (0.6) | 4.7 (1.5) | 14.6 (2.9) | 3.6 (1.8) | 19.7 (2.7) | 0.2 (0.9) | 10.9 (6.7) |
| Southern Puget Sound | 1.5 (1.3) | 0.0 (0.8) | 0.0 (0.2) | 11.0 (3.1) | 2.0 (1.2) | 13.7 (2.5) | 1.8 (4.2) | 0.0 (0.6) |
| Juan de Fuca Strait | 0.1 (0.4) | 9.5 (7.0) | 0.0 (0.1) | 4.7 (1.5) | 0.0 (0.1) | 0.6 (0.9) | 0.0 (0.7) | 0.1 (0.5) |
| Hood Canal | 0.0 (0.3) | 0.6 (1.7) | 0.0 (0.0) | 0.0 (0.1) | 0.1 (0.4) | 2.8 (1.1) | 0.0 (0.3) | 0.0 (0.3) |
| Coastal Washington | 2.8 (1.6) | 7.8 (5.9) | 5.3 (1.5) | 12.4 (2.4) | 6.3 (1.9) | 12.3 (2.1) | 0.0 (0.7) | 5.2 (4.7) |
| Columbia River | 1.0 (0.9) | 4.4 (3.8) | 0.4 (0.4) | 5.3 (1.6) | 0.0 (0.1) | 3.0 (1.0) | 0.0 (0.8) | 22.7 (8.2) |
| Oregon | 0.0 (0.2) | 0.0 (0.7) | 0.0 (0.1) | 0.0 (0.2) | 0.0 (0.1) | 0.0 (0.0) | 0.0 (0.8) | 0.0 (0.7) |

Supplementary Table 6. Estimated percentage population-specific seasonal stock compositions for adipose fin-clipped coho salmon in eight fisheries in BC, with individuals from the listed populations potentially able to be identified via PBT. Seasonal values were obtained by weighting monthly samples by catch such that not all indivduals genotyped were included in the seasonal sample. N-PBT is the number of individuals identified in the seasonal sample via PBT. WCVI is west coast of Vancouver Island.

|  | Northern troll (marked) | Northern sport | Central coast sport | Johnstone Strait sport | Strait of Georgia sport | Juan de Fuca Strait sport | WCVI sport | Barkley Sound sport |
| --- | --- | --- | --- | --- | --- | --- | --- | --- |
| Sample size | 768 | 85 | 27 | 192 | 171 | 374 | 517 | 213 |
| N-PBT | 156 | 14 | 13 | 115 | 120 | 148 | 99 | 184 |
| Quinsam | 1.0 (0.3) | 1.1 (1.3) | 24.5 (8.2) | 17.8 (2.2) | 1.9 (0.9) | 1.5 (0.6) | 0.5 (0.3) | 0.5 (0.5) |
| Puntledge | 0.0 (0.2) | 2.3 (2.7) | 0.0 (0.2) | 0.7 (0.7) | 0.0 (0.0) | 0.0 (0.0) | 0.3 (0.4) | 0.0 (0.0) |
| Big Qualicum | 0.9 (0.5) | 1.6 (1.5) | 0.0 (0.3) | 10.8 (2.1) | 9.3 (2.1) | 4.3 (1.2) | 2.4 (0.9) | 0.0 (0.1) |
| Rosewall | 0.2 (0.1) | 0.0 (0.1) | 0.0 (0.1) | 0.3 (0.1) | 1.1 (0.1) | 0.0 (0.0) | 0.4 (0.5) | 0.0 (0.0) |
| Goldstream | 2.7 (0.6) | 1.2 (0.6) | 0.0 (0.2) | 0.9 (1.0) | 0.0 (0.0) | 1.2 (1.1) | 3.2 (1.0) | 0.0 (0.0) |
| Conuma | 0.4 (0.2) | 0.0 (0.0) | 0.0 (0.2) | 0.9 (0.6) | 0.0 (0.0) | 0.0 (0.1) | 2.0 (0.7) | 4.0 (1.4) |
| Robertson | 10.7 (0.9) | 10.2 (3.1) | 0.0 (0.1) | 4.6 (1.1) | 0.4 (0.4) | 0.2 (0.2) | 7.2 (1.2) | 86.9 (2.4) |
| Nitinat | 2.8 (0.5) | 4.1 (2.2) | 6.8 (4.8) | 0.0 (0.0) | 0.0 (0.0) | 0.9 (0.5) | 3.1 (0.8) | 1.2 (0.8) |
| Capilano | 1.4 (0.4) | 0.0 (0.1) | 3.9 (3.6) | 11.0 (1.9) | 29.7 (2.9) | 6.0 (1.2) | 2.0 (0.7) | 0.6 (0.6) |
| Tenderfoot | 0.3 (0.2) | 0.2 (0.3) | 15.6 (7.9) | 4.6 (1.5) | 5.2 (1.6) | 2.4 (0.9) | 0.2 (0.1) | 0.0 (0.1) |
| Mamquam | 0.4 (0.3) | 0.0 (0.3) | 7.5 (1.8) | 0.9 (0.2) | 1.1 (0.3) | 1.0 (0.2) | 0.0 (0.0) | 0.3 (0.5) |
| Chehalis | 0.0 (0.0) | 0.0 (0.1) | 0.0 (0.1) | 3.4 (1.1) | 4.4 (1.4) | 4.6 (1.1) | 1.2 (0.3) | 0.0 (0.1) |
| Chilliwack | 0.4 (0.2) | 0.0 (0.1) | 4.0 (3.8) | 7.8 (1.6) | 14.4 (2.2) | 13.0 (1.7) | 2.9 (0.8) | 0.0 (0.0) |
| Inch | 0.1 (0.0) | 0.0 (0.0) | 0.0 (0.1) | 1.2 (0.2) | 3.0 (0.2) | 3.5 (0.6) | 0.6 (0.2) | 0.0 (0.0) |
| Norrish | 0.0 (0.0) | 0.0 (0.0) | 0.0 (0.6) | 2.4 (1.0) | 7.5 (1.8) | 1.8 (1.0) | 0.8 (0.6) | 0.1 (0.1) |
| Stave | 0.2 (0.2) | 0.0 (0.1) | 0.0 (0.3) | 1.8 (0.9) | 2.2 (1.3) | 1.5 (0.8) | 0.4 (0.6) | 0.8 (0.8) |
| Coldwater | 0.0 (0.0) | 0.0 (0.0) | 0.0 (0.1) | 0.0 (0.0) | 0.7 (0.5) | 1.2 (0.5) | 0.2 (0.2) | 0.0 (0.0) |
| Salmon | 0.0 (0.0) | 0.0 (0.1) | 0.0 (0.3) | 0.0 (0.0) | 0.4 (0.4) | 0.9 (0.3) | 0.0 (0.0) | 0.0 (0.0) |
| Nicomekl | 0.0 (0.0) | 0.0 (0.1) | 0.0 (0.3) | 0.2 (0.3) | 0.0 (0.1) | 1.5 (0.8) | 0.0 (0.0) | 0.0 (0.0) |
| Serpentine | 0.1 (0.1) | 0.0 (0.1) | 0.0 (0.3) | 0.3 (0.1) | 1.3 (0.7) | 1.2 (0.6) | 0.4 (0.2) | 0.0 (0.0) |
